# PermDroid a framework developed using proposed feature selection approach and machine learning techniques for Android malware detection

**DOI:** 10.1038/s41598-024-60982-y

**Published:** 2024-05-10

**Authors:** Arvind Mahindru, Himani Arora, Abhinav Kumar, Sachin Kumar Gupta, Shubham Mahajan, Seifedine Kadry, Jungeun Kim

**Affiliations:** 1 Department of Computer Science and applications, D.A.V. University, Sarmastpur, Jalandhar, 144012 India; 2 Department of Mathematics, Guru Nanak Dev University, Amritsar, India; 3 Department of Nuclear and Renewable Energy, Ural Federal University Named after the First President of Russia Boris Yeltsin, Ekaterinburg, Russia 620002; 4 Department of Electronics and Communication Engineering, Central University of Jammu, Jammu, 181143 UT of J&K India; 5 School of Electronics and Communication Engineering, Shri Mata Vaishno Devi University, Katra, 182320 UT of J&K India; 6 Department of Applied Data Science, Noroff University College, Kristiansand, Norway; 7 Artificial Intelligence Research Center (AIRC), Ajman University, Ajman, 346, United Arab Emirates; 8 MEU Research Unit, Middle East University, Amman 11831, Jordan; 9 Applied Science Research Center, Applied Science Private University, Amman, Jordan; 10 Department of Software, Department of Computer Science and Engineering, Kongju National University, Cheonan, 31080 Korea

**Keywords:** Android apps, API calls, Neural network, Deep learning, Feature selection, Intrusion detection, Permissions model, Engineering, Electrical and electronic engineering

## Abstract

The challenge of developing an Android malware detection framework that can identify malware in real-world apps is difficult for academicians and researchers. The vulnerability lies in the permission model of Android. Therefore, it has attracted the attention of various researchers to develop an Android malware detection model using permission or a set of permissions. Academicians and researchers have used all extracted features in previous studies, resulting in overburdening while creating malware detection models. But, the effectiveness of the machine learning model depends on the relevant features, which help in reducing the value of misclassification errors and have excellent discriminative power. A feature selection framework is proposed in this research paper that helps in selecting the relevant features. In the first stage of the proposed framework, *t*-test, and univariate logistic regression are implemented on our collected feature data set to classify their capacity for detecting malware. Multivariate linear regression stepwise forward selection and correlation analysis are implemented in the second stage to evaluate the correctness of the features selected in the first stage. Furthermore, the resulting features are used as input in the development of malware detection models using three ensemble methods and a neural network with six different machine-learning algorithms. The developed models’ performance is compared using two performance parameters: F-measure and Accuracy. The experiment is performed by using half a million different Android apps. The empirical findings reveal that malware detection model developed using features selected by implementing proposed feature selection framework achieved higher detection rate as compared to the model developed using all extracted features data set. Further, when compared to previously developed frameworks or methodologies, the experimental results indicates that model developed in this study achieved an accuracy of 98.8%.

## Introduction

Now-a-days, smartphones can do the same work as the computer has been doing. By the end of 2023, there will be around 6.64 billion smartphone users worldwide (https://www.bankmycell.com/blog/how-many-phones-are-in-the-world). According to the report (https://www.statista.com/statistics/272307/market-share-forecast-for-smartphone-operating-systems/) at the end of 2023, Android operating systems captured 86.2% of the total segment. The main reason for its popularity is that its code is written in open source which attracts developers to develop Android apps on a daily basis. In addition to that it provides many valuable services such as process management, security configuration, and many more. The free apps that are provided in its official store are the second factor in its popularity. By the end of March 2023 data (https://www.appbrain.com/stats/number-of-android-apps), Android will have 2.6 billion apps in Google play store.

Nonetheless, the fame of the Android operating system has led to enormous security challenges. On the daily basis, cyber-criminals invent new malware apps and inject them into the Google Play store (https://play.google.com/store?hl=en) and third-party app stores. By using these malware-infected apps cyber-criminals steal sensitive information from the user’s phone and use that information for their own benefits. Google has developed the Google Bouncer (https://krebsonsecurity.com/tag/google-bouncer/) and Google Play Protect (https://www.android.com/play-protect/) for Android to deal with this unwanted malware, but both have failed to find out malware-infected apps^1–3^. According to the report published by Kaspersky Security Network, 6,463,414 mobile malware had been detected at the end of 2022 (https://securelist.com/it-threat-evolution-in-q1-2022-mobile-statistics/106589/). Malware acts as a serious problem for the Android platform because it spreads through these apps. The challenging issue from the defender’s perspective is how to detect malware and enhance its performance. A traditional signature-based detection approach detects only the known malware whose definition is already known to it. Signature-based detection approaches are unable to detect unknown malware due to the limited amount of signatures present in its database. Hence, the solution is to develop a machine learning-based approach that dynamically learns the behavior of malware and helps humans in defending against malware attacks and enhancing mobile security.

Researchers and academicians have proposed different methods for analyzing and detecting malware from Android. Some of them have been proposed by using static analysis, for example, ANASTASIA^4^, DREBIN^5^, Droiddetector^6^ and DroidDet^7^. On the other side, some researchers have proposed with the help of dynamic analysis, for example, IntelliDroid^8^, DroidScribe^9^, StormDroid^10^ and MamaDroid^11^. But, the main constraints of these approaches are present in its implementation and time consumption because these models are developed with a number of features. On the other side, academicians and researchers^3,12–19^ also proposed malware detection frameworks that are developed by using relevant features. But, they have restrictions too. They implemented only already proposed feature selection techniques in their work.

So, in this research paper, to overcome the hindrances a feature selection framework is proposed. This helps in the evaluation of appropriate feature sets with the goal of removing redundant features and enhances the effectiveness of the machine-learning trained model. Further, by selecting a significant features a framework named *PermDroid* is developed. The proposed framework is based on the principle of artificial neural network with six different machine learning techniques, i.e., Gradient descent with momentum (GDM), Gradient descent method with adaptive learning rate (GDA), Levenberg Marquardt (LM), Quasi-Newton (NM), Gradient descent (GD), and Deep Neural Network (DNN). These machine learning algorithms are considered on the basis of their performance in the literature^20^. In addition to this, three different ensemble techniques with three dissimilar combination rules are proposed in this research work to develop an effective malware detection framework. F-measure and Accuracy have been considered as performance parameters to evaluate the performance. From the literature review^21–23^, it is noticed that a number of authors have concentrated on bettering the functioning of the malware detection models. However, their study had a key flaw, they only used a small amount of data to develop and test the model. In order to address this issue, this study report takes into account 500,000 unique Android apps from various categories.

**Figure 1:**
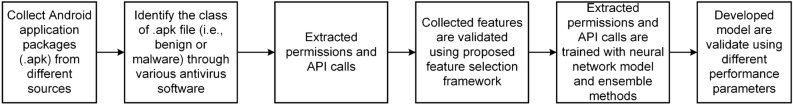
Steps are followed in developing Android malware detection framework.

The method for developing a reliable malware detection model is represented in Fig. 1. The initial collection of Android application packages (.apk) comes from a variety of promised repositories (mentioned in “Creation of experimental data set and extraction of features” section). Anti-virus software is used to identify the class of .apk files at the next level (mentioned in “Creation of experimental data set and extraction of features” section). Then, features (such as API calls and permissions) are retrieved from the .apk file using various techniques described in the literature (mentioned in subsection 3.4). Additionally, a feature selection framework is applied to evaluate the extracted features (discussed in “Proposed feature selection validation method” section). Then, a model is developed using an artificial neural network using six different machine-learning techniques and three different ensemble models, employing the selected feature sets as input. Finally, F-measure and Accuracy are taken into consideration while evaluating the developed models. The following are the novel and distinctive contributions of this paper:

- In this study, to develop efficient malware detection model half a million unique apps have been collected from different resources. Further, unique features are extracted by performing dynamic analysis in this study.
- The methodology presented in this paper, is based on feature selection methodologies, which contributes in determining the significant features that are utilized to develop malware detection models.
- In this study, we proposed three different ensemble techniques that are based on the principle of a heterogeneous approach.
- Six different machine learning algorithms that are based on the principle of Artificial Neural Network (ANN) are trained by using relevant features.
- When compared to previously developed frameworks and different anti-virus software in the market, the proposed Android malware detection framework can detect malware-infected apps in less time.
- A cost-benefit analysis shows that the proposed Android malware detection framework is more effective in identifying malware-infected apps from the real world.

The remaining sections of this research paper are arranged as follows: “Related work” section presents the literature survey on Android malware detection as well as the creation of research questions. “Research methodology” section gives an overview of the research methodology used to create the Android malware detection framework. Different machine learning and ensemble techniques are addressed in “Machine learning technique” section. The proposed feature selection validation technique is discussed in “Proposed feature selection validation method” section. The experimental results are presented in “Experimental setup and results” section. Threats to validity are presented in “Threats to validity” section. Conclusion and the future scope are discussed in “Conclusion and future work” section.

## Related work

The exploitation of the vulnerability is common these days to acquire higher privilege on Android platforms. Since 2008, cybercriminals have started targeting Android devices. An exploit app, from the perspective of Android security, can assist cyber-criminals in bypassing security mechanisms and gaining more access to users’ devices. Cybercriminals may exploit user data by selling their personal information for monetary gain if they took advantage of these privileges. The detection process, which has been used by researchers in the past and is based on Artificial Neural Networks (ANN) and feature selection techniques, is addressed in this subsection.

Androguard (https://code.google.com/archive/p/androguard/) is a static analysis tool that detects malware on Android devices using the signature concept. Only malware that is already known to be present and whose definition is in the Androguard database is identified. It cannot, however, identify unidentified malware. Andromaly^23^, is developed on a dynamic analysis tool that uses a machine learning technique. It monitored CPU utilization, data transfer, the number of effective processes, and battery usage in real-time. The test was carried out on a few different types of simulated malware samples, but not on the applications that are present in the real-world. By using the semantics of the code in the form of code graphs collected from Android apps, Badhani et al.^24^ developed malware detection methodology. Faruki et al.^21^ introduced AndroSimilar, which is based on the principles of generated signatures that are developed from the extracted features, which are used to develop malware detection model.

Aurasium^25^ takes control of an app’s execution by examining arbitrary security rules in real-time. It repackages Android apps with security policy codes and informs users of any privacy breaches. Aurasium has the problem of not being able to detect malicious behavior if an app’s signature changes. They performed dynamic analysis of Android apps and considered call-centric as a feature. The authors tested their method on over 2900 Android malware samples and found that it is effective at detecting malware activity. A web-based malware evaluation method has been proposed by Andrubis^26^, it operates on the premise that users can submit apps via a web service, and after examining their activity, it returns information on whether the app is benign or malicious. Ikram et al.^27^ suggested an approach named as DaDiDroid based on weighted directed graphs of API calls to detect malware-infected apps. The experiment was carried out with 43,262 benign and 20,431 malware-infected apps, achieving a 91% accuracy rate. Shen et al.^28^ developed an Android malware detection technique based on the information flow analysis principle. They implement N-gram analysis to determine common and unique behavioral patterns present in the complex flow. The experiment was carried out on 8,598 different Android apps with an accuracy of 82.0 percent. Yang et al.^29^ proposed an approach named EnMobile that is based on the principle of entity characterization of the behavior of the Android app. The experiment was carried out on 6,614 different Android apps, and the empirical results show that their proposed approach outperformed four state-of-the-art approaches, namely Drebin, Apposcopy, AppContext, and MUDFLOW, in terms of recall and precision.

**Table 1:** Some existing Android malware detection frameworks.

Frameworks	Detection method	Monitoring type	Analysis type
TaintDroid (2010)^30^	Dynamic	Program traces	Expert
Paranoid Android (2010)^31^	Dynamic	Program traces	Expert
AASandbox (2010)^32^	Dynamic	System and library calls	Clustering
Schmidt et al. (2011)^33^	Static and dynamic	System calls	Clustering
Crowdroid (2011)^34^	Dynamic	System calls	Clustering
Andromaly (2012)^23^	Dynamic	Behavioural monitoring	Machine learning
Aurasium (2012)^25^	Dynamic	Behavioural	Repackaging
Woodpecker (2012)^35^	Static	Permissions	Dependency graphs
RiskRanker (2012)^36^	Static	Instructions, permissions	Dependency graphs
SmartDroid (2012)^37^	Static and dynamic	Program traces	Dependency graphs
MADAM (2012)^38^	Dynamic	Kernel-level and	Machine learning
DroidScope (2012)^39^	Dynamic	Kernel-level and	Expert
AppGuard (2012)^40^	Dynamic	Program traces	Expert
TstructDroid (2013)^41^	Dynamic	Process control block	Machine learning
AppsPlayground (2013)^42^	Dynamic	System calls	Expert
AppProfiler (2013)^43^	Static and dynamic	Program traces and	Expert
Andrubis (2014)^26^	Static and dynamic	Dalvik and system level	Expert
Androguard (2015)^44^	Static	Disassemble and	Control flow graphs
CopperDroid (2015)^45^	Dynamic	System call	Hierarchical
DroidDetector (2016)^6^	Static and dynamic	Permissions, sensitive APIs and dynamic behaviors	Machine learning
MAMADROID (2016)^11^	Static	API calls	Machine learning
DroidSieve (2017)^46^	Static	Intents permissions, meta information and native code	Machine learning
PIndroid (2017)^47^	Dynamic	Permissions and intents	Machine learning
MOCDroid (2017)^48^	Static and dynamic	Behavior	Machine learning
DroidDet (2018)^7^	Static	Permissions, monitoring system events,	Machine learning
		sensitive APIs, and Permission-rate	
MalDozer (2018)^49^	Dynamic	Third-party calls	Machine learning
SeqDroid (2019)^50^	Static	Package names	Machine learning
DL-Droid (2020)^51^	Static and dynamic	Log files	Machine learning
MLDroid (2020)^3^	Dynamic	Rating of an app and,	Machine learning
		permissions	

CrowDroid^34^, which is built using a behavior-based malware detection method, comprises of two components: a remote server and a crowdsourcing app that must both be installed on users’ mobile devices. CrowDroid uses a crowdsourcing app to send behavioral data to a remote server in the form of a log file. Further, they implemented 2-mean clustering approach to identify that the app belongs to malicious or benign class. But, the crowDroid app constantly depletes the device’s resources. Yuan et al.^52^ proposed a machine learning approach named Droid-Sec that used 200 extracted static and dynamic features for developing the Android malware detection model. The empirical result suggests that the model built by using the deep learning technique achieved a 96% accuracy rate. TaintDroid^30^ tracks privacy-sensitive data leakage in Android apps from third-party developers. Every time any sensitive data leaves the smartphone, TaintDroid records the label of the data, the app that linked with the data, as well as the data’s destination address.

Zhang et al.^53^ proposed a malware detection technique based on the weighted contextual API dependency graph principle. An experiment was performed on 13500 benign samples and 2200 malware samples and achieved an acceptable false-positive rate of 5.15% for a vetting purpose.

AndroTaint^54^ works on the principle of dynamic analysis. The features extracted were used to classify the Android app as dangerous, harmful, benign, or aggressive using a novel unsupervised and supervised anomaly detection method. Researchers have used numerous classification methods in the past, like Random forest^55^, J48^55^, Simple logistic^55^, Naïve Bayes^55^, Support Vector Machine^56,57^, K-star^55^, Decision tree^23^, Logistic regression^23^ and k-means^23^ to identify Android malware with a better percentage of accuracy. DroidDetector^6^, Droid-Sec^52^, and Deep4MalDroid^58^ work on the convention of deep learning for identifying Android malware. Table 1 summarizes some of the existing malware detection frameworks for Android.

### The artificial neural network (ANN) technique is used to identify malware on Android devices

Nix and Zhang^59^ developed a deep learning algorithm by using a convolution neural network (CNN) and used API calls as a feature. They utilized the principle of Long Short-Term Memory (LSTM) and joined knowledge from its sequences. McLaughlin et al.^60^, implemented deep learning by using CNN and considered raw opcode as a feature to identify malware from real-world Android apps. Recently, researchers^6,58^ used network parameters to identify malware-infected apps. Nauman et al.^61^, implemented connected, recurrent, and convolutional neural networks, and they also implemented DBN (Deep Belief Networks) to identify malware-infected apps from Android. Xiao et al.^62^, presented a technique that was based on the back-propagation of the neural networks on Markov chains and considered the system calls as a feature. They consider the system call sequence as a homogenous stationary Markov chain and employed a neural network to detect malware-infected apps. Martinelli et al.^63^, implemented a deep learning algorithm using CNN and consider the system call as a feature. They performed an experiment on a collection of 7100 real-world Android apps and identify that 3000 apps belong to distinct malware families. Xiao et al.^64^, suggested an approach that depends on the principle of LSTM (Long Short-Term Memory) and considers the system call sequence as a feature. They trained two LSTM models by the system call sequences for both the benign and malware apps and then compute the similarity score. Dimjas̈evic et al.^65^, evaluate several techniques for detecting malware apps at the repository level. The techniques worked on the tracking of system calls at the time the app is running in a sandbox environment. They performed an experiment on 12,000 apps and able to identify 96% malware-infected apps.

### Using feature selection approaches, to detect Android malware

Table 2 shows the literature review for malware detection done by implementing feature selection techniques. Mas’ud et al.^66^ proposed a functional solution to detect malware from the smartphone and can address the limitation of the environment of the mobile device. They implemented chi-square and information gain as feature selection techniques to select the best features from the extracted dataset. Further, with the help of selected best features, they employed K-Nearest Neighbour (KNN), Naïve Bayes (NB), Decision Tree (J48), Random Forest (RF), and Multi-Layer Perceptron (MLP) techniques to identify malware-infected apps. Mahindru and Sangal^3^ developed a framework that works on the basis of feature selection approaches and used distinct semi-supervised, unsupervised, supervised, and ensemble techniques parallelly and identify 98.8% malware-infected apps. Yerima et al.^67^ suggested an effective technique to detect malware from smartphones. They implemented mutual information as a feature selection approach to select the best features from the collected code and app characteristics that indicate the malicious activities of the app. To detect malware apps, from the wild, they trained selected features by using Bayesian classification and achieved an accuracy of 92.1%. Mahindru and Sangal^15^ suggested a framework named as “PerbDroid” that is build by considering feature selection approaches and deep learning as a machine classifier. 2,00,000 Android apps in total were subjected to tests, with a detection rate of 97.8%. Andromaly^23^ worked on the principle of the Host-based Malware Detection System that monitors features related to memory, hardware, and power events. After selecting the best features by implementing feature selection techniques, they employed distinct classification algorithms such as decision tree (J48), K-Means, Bayesian network, Histogram or Logistic Regression, Naïve Bayes (NB) to detect malware-infected apps. Authors^14^ suggested a malware detection model based on semi-supervised machine learning approaches. They examined the proposed method on over 200,000 Android apps and found it to be 97.8% accurate. Narudin et al.^68^ proposed a malware detection approach by considering network traffic as a feature. Further, they applied random forest, multi-layer perceptron, K-Nearest Neighbor (KNN), J48, and Bayes network machine learning classifiers out of which the K-Nearest Neighbor classifier attained an 84.57% true-positive rate for detection of the latest Android malware. Wang et al.^69^ employed three different feature ranking techniques, i.e., *t*-test, mutual information, and correlation coefficient on 3,10,926, benign, and 4,868 malware apps using permission and detect 74.03% unknown malware. Previous researchers implement feature ranking approaches to select significant sets of features only. Authors^13^ developed a framework named as “DeepDroid” based on deep learning algorithm. They use six different feature ranking algorithms on the extracted features dataset to select significant features. The tests involved 20,000 malware-infected apps and 100,000 benign ones. The detection rate of a framework proposed using Principal component analysis (PCA) was 94%. Researchers and Academicians^70–73^ also implemented features selection techniques in the literature in different fields to select significant features for developing the models.

### Research questions

To identify malware-infected apps and considering the gaps that are present in the literature following research questions are addressed in this research work:

- *RQ1* Does the filtering approach helps to identify that whether an app is a benign or malware-infected (first phase of the proposed feature selection framework)? To determine the statistical significance among malicious and benign apps, the *t*-test is used. After, determining significant features, a binary ULR investigation is applied to select more appropriate features. For analysis, all the thirty different feature data sets are assigned (shown in Table 5) as null hypotheses.
- *RQ2* Do already existing and presented work’s sets of features show an immense correlation with each other? To answer this question, both positive and negative correlations are examined to analyze the sets of features, which help in improving the detection rate.
- *RQ3* Can the identified features assist in determining whether the app is malware-infected or not? The primary objective of this question is to use the feature selection framework validation approach to determine the appropriate features. In this paper, four stages (i.e., ULR, t-test, Correlation analysis, and multivariate linear regression stepwise forward selection) are implemented to identify the appropriate features, that helps in identifying whether an app contains malicious behavior or not.
- *RQ4* Which classification algorithm among the implemented machine learning algorithms is most appropriate for identifying malware-infected apps? To answer to this question the efficiency of various machine learning approaches are evaluated. In this study, three different ensemble approaches and six different machine learning algorithms based on neural networks are considered.
- *RQ5* Is the feature collected (such as an app’s rating, API calls, permissions, and the number of people who have downloaded the app) sufficient for identifying a malicious app or not? This question helps in determining whether or not considering features can detect malware-infected apps in the real world. To answer this question, the performance of our suggested model is compared with previously published frameworks as well as several anti-virus scanners in the market.

## Research methodology

Based on the research questions mentioned above, the methodology that is used in this research paper is mentioned in the following subsections. In order to improve the detection rate for malware, the obtained data set has been normalized, and dependent and independent variables have been selected.

### Independent variables

**Figure 2:**
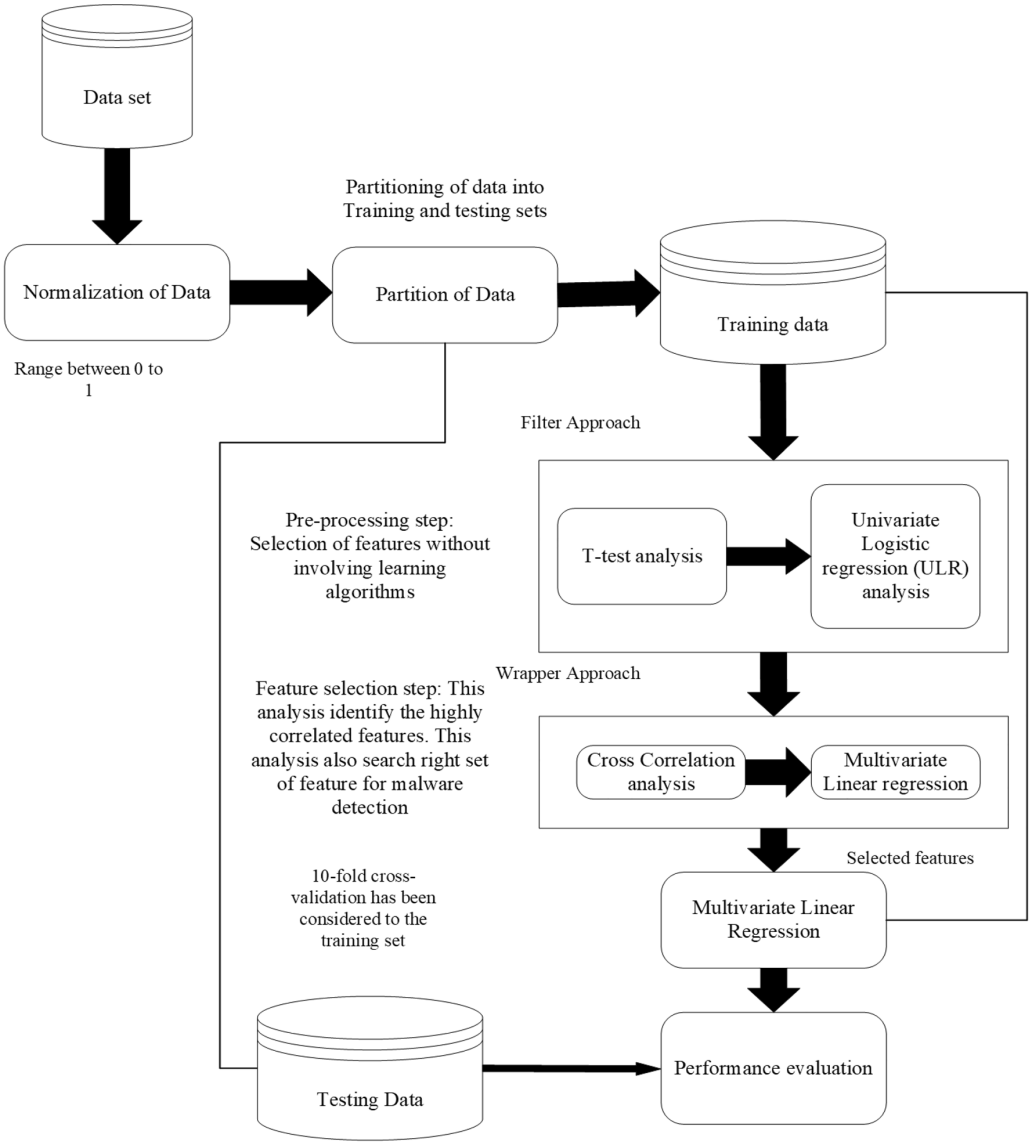
Proposed framework for feature selection and its validation.

In this study, the model is developed by applying the proposed feature selection approach, which helps in the detection of malware-infected apps. Additionally, as shown in Fig. 2, five different strategies to select the best features are used. The best features are selected from other accessible features created on intermediate explore models at each level.

### Dependent variables

The focus of this research is to find a link between Android apps and the features (such as app rating, API calls, permission, and the number of users who have downloaded an app) retrieved from the collected data set. The malware app characteristics are separated from the benign app features in the dependent variable of Android apps.

### Creation of experimental data set and extraction of features

In this research paper, 70,000 .apk files from Google play store (https://play.google.com/store?hl=en), and more than 3 lacs .apk files from third-party app store i.e., Softonic (https://en.softonic.com/android), Android Authority (https://www.androidauthority.com/apps/), CNET (https://download.cnet.com/android/) belong to

**Table 2:** In the literature, there are feature selection methods and machine learning algorithms that have been implemented.

Author/approach	The technique for selecting features was used	Machine learning algorithm used
ANASTASIA^4^	Randomized tree group	Decision tree (J48), Support vector machine (SVM),
	(i.e., Extra trees-classifier)	Naïve Bayes (NB), Logistic regression,
		K-Nearest neighbours, random forest(RF),
		Deep learning, and Adaboost
Andromaly^23^	Chi-square, Fisher score and Information gain	k-Means, Naïve Bayes (NB),
		Bayesian network, decision tree (J48)
		Histogram or logistic regression
Mas’ ud et al.^66^	Information gain and Chi-square test	Naïve Bayes (NB), K-nearest Neighbour (KNN),
		Decision Tree (J48), Multi-layer perceptron (MLP),
		and random forest (RF)
Allix et al.^74^	Information gain	Support vector machine (SVM), C4.5,
		RIPPER, and Random forest
Yerima et al.^67^	Mutual information	Bayesian classification
MKLDroid^75^	Chi-squared	Kernel methods
Azmoodeh et al.^76^	Information gain	Deep Eigenspace learning approach
Chen et al.^77^	Using manual pruning while gaining information	Random forest (RF), support vector machine (SVM), )
		and K-nearest neighbor (KNN)
Narudin et al.^68^	ClassifierSubsetEval	Random forest, Multi-layer perceptron,
		J48, K-Nearest neighbours, and Bayes network
Yerima et al.^78^	Information gain	Bayesian classifier

**Figure 3:**
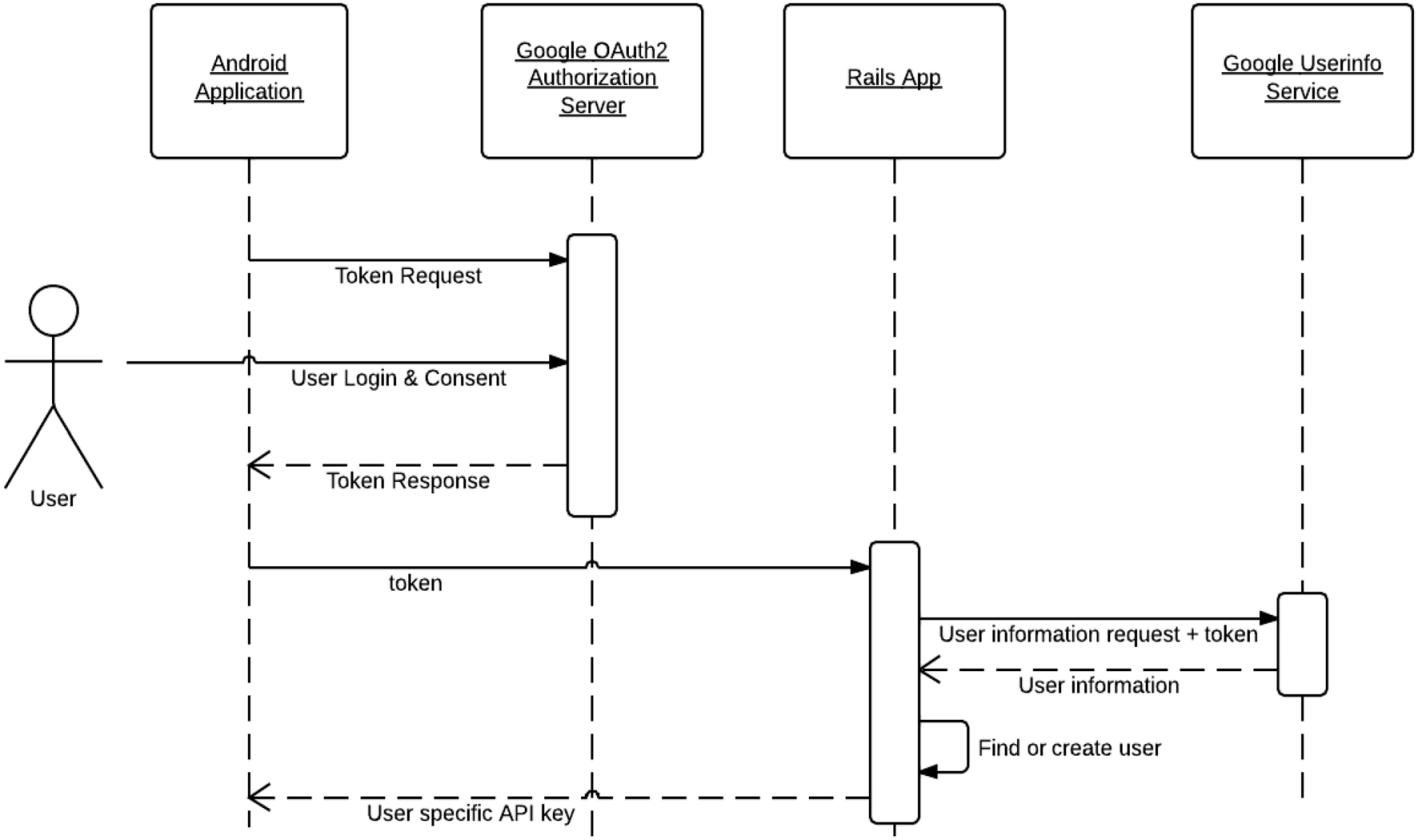
Sequence diagram showing reservation using Android app.

**Table 3:** Number of apps consider in our study.

ID	Category of Android app	Downloaded from Google Play store	Downloaded from Third-party app store	Benign apps	Malware families										Malware apps
					AD	BA	HT	RA	TR	TB	TC	TD	TS	TSY	
D1	Arcade & Action(AA)	2291	11856	14147	5500	290	210	23	5580	35	30	20	29	20	11737
D2	Books & Reference(BR)	2235	12513	14748	40	100	100	40	100	0	10	20	7	10	427
D3	Brain & Puzzle(BP)	1928	12522	14450	300	5500	300	230	5050	23	22	23	40	13	11501
D4	Business(BU)	1308	12162	13470	2220	30	92	10	22	12	19	0	0	3	2408
D5	Cards & Casino(CC)	886	12106	12992	100	100	230	220	221	190	23	11	5	0	1100
D6	Casual(CA)	2010	12535	14545	33	0	33	33	120	12	89	0	0	23	343
D7	Comics(CO)	667	1254	1921	30	29	90	100	12	13	19	20	33	53	399
D8	Communication(COM)	1414	11106	12520	22	33	89	90	100	47	20	0	0	2	403
D9	Education(ED)	1744	12210	13954	100	200	30	90	200	300	190	20	12	222	1364
D10	Entertainment(EN)	4222	12340	16562	100	5000	0	5000	0	200	200	200	0	0	10750
D11	Finance(FI)	999	12198	13197	20	23	33	122	20	20	0	0	10	5	253
D12	Health & Fitness(HF)	1551	11509	13060	0	0	0	0	90	0	89	60	0	4	243
D13	Libraries & Demo(LD)	655	12134	12789	10	10	10	10	10	20	30	0	7	30	137
D14	Lifestyle(LS)	2650	1735	4385	20	100	300	90	100	500	20	90	80	119	1419
D15	Media & Video(MV)	1019	11123	12142	20	20	20	90	10	10	10	20	0	27	227
D16	Medical(ME)	768	1462	2230	10	0	0	0	40	2	0	0	0	90	142
D17	Music & Audio(MA)	1621	11187	12808	20	20	20	20	20	40	40	40	20	39	279
D18	News & Magazines(NM)	1164	11115	12279	20	0	0	0	0	0	0	45	100	50	215
D19	Personalization(PE)	4334	11616	15950	0	100	20	50	50	50	45	100	200	16	631
D20	Photography(PH)	1133	11189	12322	10	10	20	30	40	10	10	10	10	7	157
D21	Productivity(PR)	1850	1377	3227	230	20	10	0	0	12	100	30	120	66	588
D22	Racing(RA)	766	1184	1950	20	0	0	0	0	0	0	50	0	0	70
D23	Shopping(SH)	1873	17134	14007	10	10	10	10	40	40	90	100	800	91	1201
D24	Social(SO)	1159	12171	13330	20	15	15	12	10	90	300	300	10	637	835
D25	Sports(SP)	1689	12447	14136	200	0	5000	5000	700	0	0	30	0	315	11245
D26	Sports Games(SG)	889	12155	13044	20	300	300	20	29	321	22	34	34	63	1143
D27	Tools(TO)	3346	10715	4061	2001	230	200	100	109	100	122	23	19	0	2904
D28	Transportation(TR)	796	11203	11999	0	227	20	23	34	5000	0	23	11	0	5338
D29	Travel & Local(TL)	2180	1582	3762	20	21	21	42	32	24	34	100	100	119	513
D30	Weather(WR)	853	12160	13013	200	200	200	3000	0	100	100	300	0	8	4108

**Table 4:** Formulation of feature data set.

Set number	Description	Set number	Description
S 1	SYNCHRONIZATION _DATA	S 2	CONTACT_INFORMATION
S 3	PHONE_STATE and PHONE_CONNECTION	S 4	AUDIO and VIDEO
S 5	SYSTEM_SETTINGS	S 6	BROWSER_INFORMATION
S 7	BUNDLE	S 8	LOG_FILE
S 9	LOCATION_INFORMATION	S 10	WIDGET
S 11	CALENDAR_INFORMATION	S 12	ACCOUNT_SETTINGS
S 13	DATABASE_INFORMATION	S 14	IMAGE
S 15	UNIQUE_IDENTIFIER	S 16	FILE_INFORMATION
S 17	SMS_MMS	S 18	READ
S 19	ACCESS_ACTION	S 20	READ_AND_WRITE
S 21	YOUR_ACCOUNTS	S 22	STORAGE_FILE
S 23	SERVICES_THAT_COST_YOU_MONEY	S 24	PHONE_CALLS
S 25	SYSTEM_TOOLS	S 26	NETWORK_INFORMATION
			and BLUETOOTH_INFORMATION
S 27	HARDWARE_CONTROLS	S 28	Default group
S 29	API calls	S 30	Rating and number of user downlaods

**Table 5:** Null hypothesis.

Hypothesis	Description	Hypothesis	Description
H 1	Set of features S1 does not detect malware-infected apps	H 2	Set of features S2 does not detect malware-infected apps
H 3	Set of features S3 does not detect malware-infected apps	H 4	Set of features S4 does not detect malware-infected apps
H 5	Set of features S5 does not detect malware-infected apps	H 6	Set of features S6 does not detect malware-infected apps
H 7	Set of features S7 does not detect malware-infected apps	H 8	Set of features S8 does not detect malware-infected apps
H 9	Set of features S9 does not detect malware-infected apps	H 10	Set of features S10 does not detect malware-infected apps
H 11	Set of features S11 does not detect malware-infected apps	H 12	Set of features S12 does not detect malware-infected apps
H 13	Set of features S13 does not detect malware-infected apps	H 14	Set of features S14 does not detect malware-infected apps
H 15	Set of features S15 does not detect malware-infected apps	H 16	Set of features S16 does not detect malware-infected apps
H 17	Set of features S17 does not detect malware-infected apps	H 18	Set of features S18 does not detect malware-infected apps
H 19	Set of features S19 does not detect malware-infected apps	H 20	Set of features S20 does not detect malware-infected apps
H 21	Set of features S21 does not detect malware-infected apps	H 22	Set of features S22 does not detect malware-infected apps
H 23	Set of features S23 does not detect malware-infected apps	H 24	Set of features S24 does not detect malware-infected apps
H 25	Set of features S25 does not detect malware-infected apps	H 26	Set of features S26 does not detect malware-infected apps
H 27	Set of features S27 does not detect malware-infected apps	H 28	Set of features S28 does not detect malware-infected apps
H 29	Set of features S29 does not detect malware-infected apps	H 30	Set of features S30 does not detect malware-infected apps

benign group and 70,000 malware-infected Android apps from^79–81^ and Sanddroid (http://sanddroid.xjtu.edu.cn:8080/) belongs to malicious group are collected to develop an effective malware detection framework. As seen in Table 3, the .apk files we collected fall under thirty different categories. Collected malware-infected apps belong to ten different malware categories: AD (Adware), BA (Backdoor), HT (Hacker Tool), RA (Ransom), TR (Trojan), TB (Trojan-Banker), TC (Trojan-Clicker), TD (Trojan-Dropper), TS (Trojan-SMS) and TSY (Trojan-Spy). Classes are identified by using two distinct scanners i.e., VirusTotal (https://www.virustotal.com/gui/) and Microsoft Windows Defender (https://windows-defender.en.softonic.com/download) and on the basis of its behavior defined in the study^82^.

To formulate an efficient malware detection framework, we extract 310 API calls and 1419 unique permissions (https://github.com/ArvindMahindru66/Computer-and-security-dataset), by implementing the procedure mentioned in the literature^3,13,15,83^ . If an app requests the permission and API call during installation or runtime, we mark it as “1”; otherwise, we mark it as “0”. The following are some of the features of a certain app that have been extracted:

0,1,1,1,1,0,0,0,0,0,1,1,1,1,1,1,1,1,1,1,1,1,1,1,1,1,

0,0,0,0,0,0,0,0,0,0,1,1,1,1,1,1,1,1,1,1,1,1,1,0,0,0,

1,1,1,1,1,1,1,1,1,1,1,1,1,1,1,1,1,1,1,1,1,1,1,1,1,1,

1,1,1,1,1,1,1,1,1,1,0,0,0,0,0,0,0,0,0,0,0,0,0,0,0,0, and so on.

After extracting API calls and permissions from the collected data set from .apk files, it is divided into thirty different features data sets (Mahindru, Arvind (2024), “Android Benign and Malware Dataset”, Mendeley Data, V1, doi: 10.17632/rvjptkrc34.1). Table 4 illustrates the creation of various feature data sets as well as their explanations. These extracted features are divided into different sets on the basis of its behavior to which it belongs^3,13,15,83^. The main reasons to divide these extracted features into thirty different feature data sets are: to select significant features by using the proposed feature selection framework and to remove the complexity.

Figure 3 demonstrate the sequence diagram of an Android app by showing the example of an railway reservation app. How the process is started and how it is interact with other APIs and the permissions that are running in the background (Table 5).

## Machine learning technique

**Figure 4:**
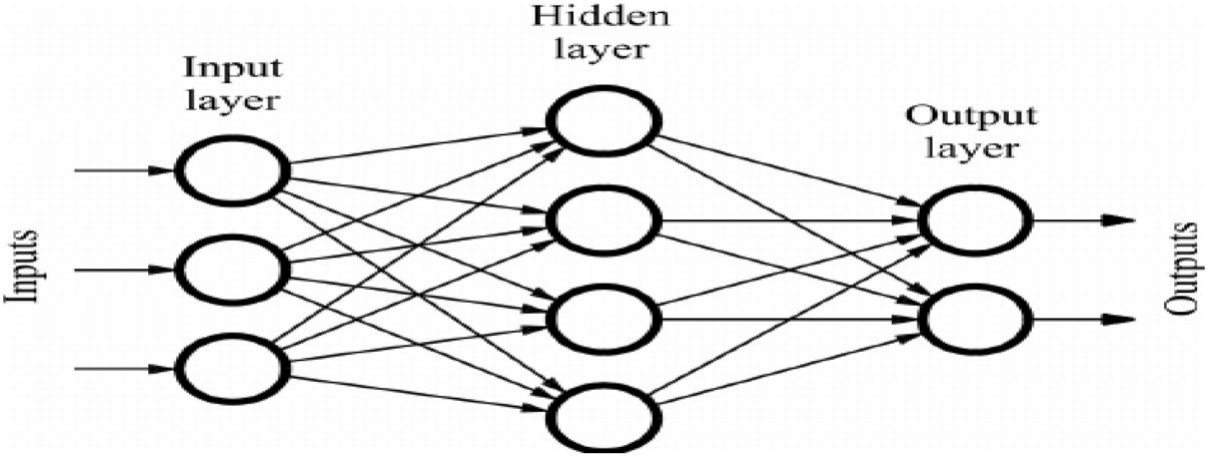
Artificial neural network.

ANN stands for artificial neural networks, and it is a computing system based on biological neural networks. These are able to perform certain tasks by utilizing certain examples, without using task-specific rules. Researchers are implementing ANN to solve different problems in malware detection, pattern recognition, classification, optimization, and associative memory^84^. In this paper, ANN is implemented to create a malware detection model. The structure of the ANN model is shown in Fig. 4. ANN contains input nodes, hidden nodes, and output nodes.

The input layer employs a linear stimulation function, while the hidden and output layers employ squashed-S or sigmoidal functions. ANN can be presented as:

1 $$\begin{aligned} O^{'}=f(A, B), \end{aligned}$$

where *B* is the input vector, *A* is the weight vector and $O^{'}$ denotes the desired output vector. In order to minimize the mean square error (MSE), the value of *A* is updated in each step. Mean square error can be calculated from the equation below:

2 $$\begin{aligned} \text{ Mean } \text{ square } \text{ error }=\frac{1}{n}\sum _{i=1}^{n}(O^{'i}-O_i)^2. \end{aligned}$$

Here, *O* is the actual output value, and $O^{'}$ is the desired output value. Various methods were proposed by researchers^20,84^ to train the neural network. In this research work, six different kinds of machine learning algorithms (namely, Gradient Descent approach, Quasi-Newton approach, Gradient Descent with Momentum approach, Levenber-Marquardt approach, Gradient Descent with Adaptive learning rate approach, and Deep neural network) are considered to develop malware detection model. These models are effective in the field of software fault prediction^20^, intrusion detection and desktop malware predictions^85^ too.

### Gradient descent with momentum approach

This approach accelerates the rate of convergence dramatically^20,84^. To obtain new weights, this approach combines the fraction diversity^20,84,86^. *X* is the updated weighed vector defined as:

3 $$\begin{aligned} X_{k+1}=A*X_k-\alpha \frac{\partial }{\partial X}(E_k), \end{aligned}$$

where *A* denotes the momentum parameter value, $X_k$ is the current weight vector and $X_{k+1}$ is the update value of the weight vector and $(E_k)$, used to identify the lower value in error space. Here, $X_{k+1}$ relys on both the weight and the gradient. To determine the optimal value of *A* we implemented the cross-validation technique.

### Gradient descent approach

This approach updates the weights to reduce the output error^20,84,86^. In Gradient descent (GD) approach, to identify the lower value in error space $(E_k)$, the $1^{st}$ - order derivative of the total error function is computed by considering, the following equation:

4 $$\begin{aligned} G=\frac{\partial }{\partial X}(E_k)=\frac{\partial }{\partial X}\Bigl (\frac{1}{2}(O^{'}_k-O_k)^2\Bigr ). \end{aligned}$$

Redundancy weight vector *X* is modified by employing gradient vector *G*^20,84,86^. The up-dation of *X* is done through the following formula

5 $$\begin{aligned} O_{n+1}=-\alpha G_n=-\alpha \frac{\partial }{\partial O}(E_n), \end{aligned}$$

where $G_n$ is the gradient vector, $O_{x+1}$ is the revised weight vector and $\alpha$ is the gaining constant. To calculate the optimum value of $\alpha$, we implement cross-validation approach.

### Gradient descent method with adaptive learning rate approach

In the GD approach, during training, the learning rate $(\alpha )$ remains stable. This approach is based on the concept that is quite perceptive to the approximation value of the learning rate. At the time of training, if the value of the learning rate is too high, the build model can be highly unstable and oscillate its value^20^. On the reverse of this, if the training value is too small, the procedure may take a long way to converge. Practically, it is not easy to find out the optimal value of $\alpha$ before training. Actually, during the training process, the value of $\alpha$ changes^20^. In each iteration, if the performance decline along with the required aim, the $\alpha$ value is added by 1.05,  and in reverse of this, if the performance increase by more than the factor of 1.04,  then the $\alpha$ value is incremented by 0.7^20^.

### Levenberg Marquardt (LM) approach

The foundation of LM is an iterative technique that helps in locating the multivariate function’s minimal value. At the time of training, this value can be calculated as the sum of squares of real-valued with non-linera functions which helps in modifying the weights^20,87^. This method is quite stable and fast because it combines the Gauss Newton and the steepest descent approach. The iterative process for the same is given by

6 $$\begin{aligned} X_{k+1}=X_k-(J_k^TJ_k+\mu I)^{-1}J_ke_k \end{aligned}$$

where $X_{k+1}$ is the updated weight, $X_k$ is the current weight, *I* is the identity matrix, $\mu >0$ is named as combination coefficient and *J* is the Jacobian matrix. For a small value of $\mu ,$ it becomes Gauss-Newton approach and for large, $\mu ,$ it acts as GD approach. Representation of Jacobian matrix is : $J=$ $$\begin{bmatrix} \frac{\partial E_{1,1}}{\partial X_1}& \frac{\partial E_{1,1}}{\partial X_2}&\cdots &\frac{\partial E_{1,1}}{\partial X_N}\\ \frac{\partial E_{1,2}}{\partial X_1}& \frac{\partial E_{1,2}}{\partial X_2}&\cdots &\frac{\partial E_{1,2}}{\partial X_N}\\ \vdots &\vdots &\vdots &\vdots \\ \frac{\partial E_{P,M}}{\partial X_1}& \frac{\partial E_{P,M}}{\partial X_2}&\cdots &\frac{\partial E_{P,M}}{\partial X_N}\\ \end{bmatrix}$$ where *P*, *N* and *M* is the input patterns, weights and the output patterns.

### Quasi-Newton approach

In order to compute the total error function, this approach requires the evaluation of the second order derivatives for each component of the gradient vector^20,84^. The iterative scheme for the Weight vector *X* is given as:

7 $$\begin{aligned} X_{k+1}=X_k-H_k^{-1}\frac{\partial }{\partial X}(E_k), \end{aligned}$$

where $X_k$ and $X_{k+1}$ are the current and updated weight vectors, accordingly. *H* is the Hessian matrix given by $H=$ $$\begin{bmatrix} \frac{\partial ^2E}{\partial X_1^2}& \frac{\partial ^2E}{\partial X_1X_2}&\cdots &\frac{\partial ^2E}{\partial X_1X_N}\\ \frac{\partial ^2E}{\partial X_1X_2}& \frac{\partial ^2E}{\partial X_2^2}&\cdots &\frac{\partial ^2E}{\partial X_2X_N}\\ \vdots &\vdots &\vdots &\vdots \\ \frac{\partial ^2E}{\partial X_1X_N}& \frac{\partial ^2E}{\partial X_2X_N}&\cdots &\frac{\partial ^2E}{\partial X_N^2} \end{bmatrix}$$

### Deep learning neural network (DNN) approach

**Figure 5:**
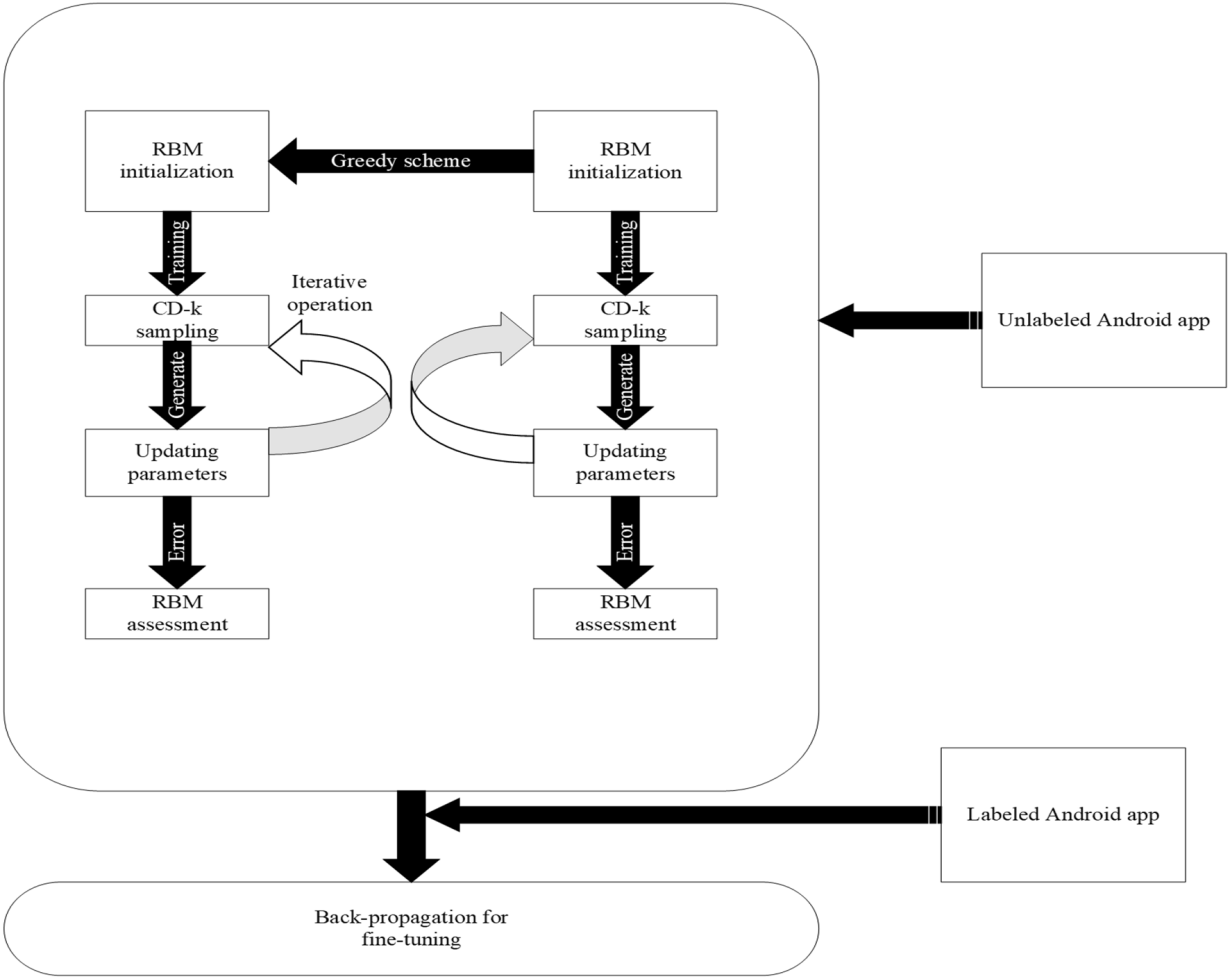
Deep learning neural network (DNN) method constructed with DBN.

Convolutional Neural Networks (CNN) and Deep Belief Networks (DBN) are two deep architectures^88^ that can be combined to create DNN. In this article, the DBN architecture to build our deep learning approach is implemented. The architecture of the deep learning method is demonstrated in Fig. 5. The procedure is separated into two stages: supervised back-propagation and unsupervised pre-training. Restricted Boltzmann Machines (RBM) with a deep neural network is used to train the model with 100 epoches in the early stages of development. An iterative method is implemented to construct the model with unlabeled Android apps in the training step. Pre-trained DBN is fine-tuned with labeled Android apps in a supervised manner during the back-propagation step. In both stages of the training process, a model developed using deep learning methods uses an Android app.

### Ensembles of classification models

In this study, three different ensemble models to detect malware from Android apps is also proposed. During development of the model, the outputs of all the classification models have been considered where the base machine learning algorithm allocated several priority levels and output is calculated by applying some combination rules. Ensemble approaches are divided into two types:

- Homogenous ensemble approach: In this approach, all classification models, are of the same kinds, but the difference is in generating the training set.
- Heterogenous ensemble approach: Here, all base classification approaches are of distinct types.

On the basis of combination rules, ensemble approaches are divided into two distinct categories:

- Linear ensemble approach: While developing the model, with a linear ensemble approach an arbitrator combines the results that come from the base learners, i.e., selection of classification approach, average weighted, etc.
- Nonlinear ensemble approach: While developing the model, with the nonlinear ensemble approach, it fed the result of the base classifier, which is a nonlinear malware detection model for example Decision tree (DT), Neural network (NN), etc.

In this work, a heterogenous ensemble approach having three distinct combination rules is adapted. The ensemble techniques are detailed in Table 6.

**Table 6:** Classification models ensembles.

Ensemble method	Base learners	Rules for combination
Heterogenous	NN with six distinct training algorithm such as DNN, NM, GDX, GD, GDX, and LM	Linear (best in training)
Heterogenous	NN with six distinct training algorithm such as DNN, NM, GDX, GD, GDX, and LM	Linear (majority voting)
Heterogenous	NN with six distinct training algorithm such as DNN, NM, GDX, GD, GDX, and LM	Non-Linear (DTF)

#### BTE (best training ensemble) approach

The BTE technique is based on the observation that each classifier performs differently when the data set is partitioned^20^. Among the applied classifier, the best model is selected to train data set that are founded on the principles of certain performance parameters. In this research paper, accuracy is considered as a performance parameter. Algorithm 1 given below is considered to calculate the ensemble output $E_{result}$.

**Algorithm 1:**
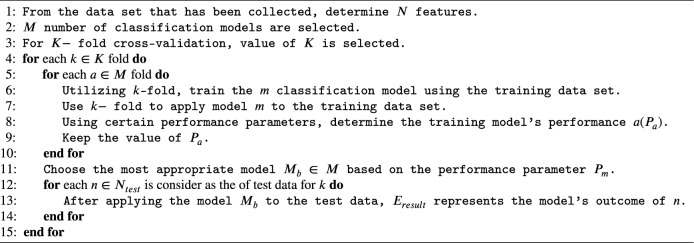
Best Training Ensemble (BTE) approach.

#### MVE (majority voting ensemble) approach

MVE approach, based on the principle to consider the output of the test data for each classifier, and the ensemble output $(E_{result})$ is concerned with the majority group differentiated by the base classifier^20^. Ensemble output $(E_{result})$ is calculated by implementing Algorithm 2.

**Algorithm 2:**
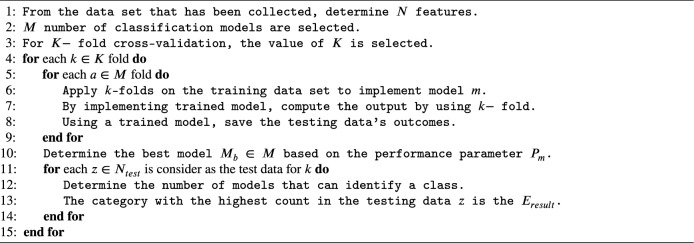
Majority Voting Ensemble (MVE) Approach.

#### NDTF (nonlinear ensemble decision tree forest) approach

In this study, to train the model with base leaner, is also considered. Further, the trained model is implemented the results on the corresponding testing data set to make the model for the final detection of malware apps. In this research paper, Decision tree forest (DTF) has been considered as a non-linear ensemble as a classifier which was suggested by Breiman in 2001. The developed model is based on the outcome of the collected results of the distinct decision trees. Algorithms 3 is used to calculate the result $(E_{result})$.

**Algorithm 3:**
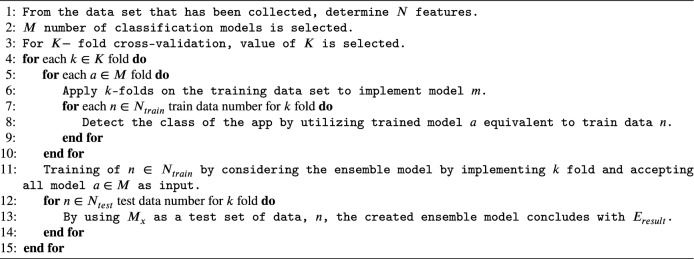
Nonlinear Ensemble Decision Tree Forest (NDTF) Approach.

### Method for normalizing the data

In order to comply with the required diversity of input properties and prevent the saturation of the neurons, it is important to normalize the data prior to deploying a neural network spanning the range of 0 to 1^89^. The Min-max normalizing approach is used in this research study. This technique is work on the principle of a linear transformation, which brings each data point $D_{q_i}$ of feature *Q* to a normalized value $D_{q_i},$ that lies in between $0-1.$

To obtain the normalized value of $D_{q_i}:$, use the following equation:

8 $$\begin{aligned} Normalized(D_{q_i})=\frac{D_{q_i}-min(Q)}{max(Q)-min(Q)},\end{aligned}$$

The relative values of the relevance of the characteristic *Q* are *min*(*Q*) and *max*(*Q*).

### Parameters considered for evaluation

This section provides definitions for the performance metrics needed to identify malicious apps. The confusion matrix is used to determine all of these characteristics. Actual and detected classification information is included in the confusion matrix, which was created using a detection approach. The constructed confusion matrix is shown in Table 7. F-measure and accuracy are two performance parameters that are used to evaluate the performance of malware detection algorithms in this research. Formulas for evaluating the accuracy and F-measure are given below:

- *False positive (FP)* A false positive occurs when the developed model identifies the positive class incorrectly.
- *False negative (FN)* When the developed model successfully identifies the negative class, a false negative occurs.
- *True negative (TN)* An accurate identification of the negative class by the developed model represents a true negative conclusion.
- *True positives (TP)* An accurate identification of the positive class by the developed model represents a real positive conclusion.
- *Recall* The data set’s positive classes that are made up of all other positive classes are identified.
  9 $$\begin{aligned} Recall =\frac{x}{x + z}, \end{aligned}$$
  where $$x= N_{Malware\rightarrow Malware},$$ $$z= N_{Malware\rightarrow Benign}$$
- *Precision* The accuracy measures the proportion of forecasts in the positive class that are indeed in the positive class.
  10 $$\begin{aligned} Precision =\frac{x}{x + y}. \end{aligned}$$
  where $$y= N_{Benign\rightarrow Malware}$$

*Accuracy* Accuracy is measured as^3^:

11 $$\begin{aligned} Accuracy=\frac{x+w}{N_{classes}}, \end{aligned}$$

where $${N_{classes} = x+y+z+w}$$,

$$w= N_{Benign\rightarrow Benign}$$

*F-measure* F-measure is measured as^3^:

12 $$\begin{aligned} F-measure=&\frac{2*Recall*Precision}{Recall+Precision}\nonumber \\&=\frac{2*x}{2*x+y+z} \end{aligned}$$

**Table 7:** An Android app’s maliciousness can be determined using a confusion matrix.

	Benign	Malware
Benign	Benign-> Benign (TP)	Benign-> Malware (FP)
Malware	Malware-> Benign (FN)	Malware-> Malware (TN)

## Proposed feature selection validation method

The selection of relevant feature sets is an important challenge for data processing in various machine learning and data mining applications^90–92^. In the field of Android malware detection, a number of authors^13–15,69,93,94^ applied only limited feature subset selection and feature ranking approaches i.e., Correlation, Goodman Kruskals, Information Gain, Chi-squared, Mutual Information, and t-test methods to detect malware. The first limitation of the previous studies is that they used a small data set (i.e., the number of malware or benign apps is less in number) to validate the proposed techniques. The additional significant disadvantage of the feature selection lies in the fact that after selecting the best features no comparison analyses were made among the classifiers model developed by reduced sets of features and by using all extracted feature sets. Mainly, the main reason for this is that the vast collection of features found in particular categories of the app (like books, entertainment, comics, game, etc.) makes it complex to produce a classifier by examining all the features as input. It is the best of our knowledge, that academicians and researchers were implemented these feature selection approaches individually; but no one selected features by combining all of these feature selection approaches. However, a framework for the feature selection approach has been given in this study, which helps in selecting the most appropriate features and enhance the effectiveness of the malware detection model. The suggested framework is applied to apps that have been gathered from the various repositories listed in section 2.4 and that fall under the thirty categories listed in Table 3. Finally, we verified the framework by comparing the effectiveness of the models developed after implementing feature selection method with the efficiency of ones constructed using the whole data set initially formed.

Figure 2 demonstrates the phases of the proposed feature selection validation framework. Without using machine learning algorithms, this framework aims to determine whether the selected features are useful in detecting malicious apps. The wrapper strategy is used to pick the sets of features that are useful in identifying malware apps after all crucial components have been examined. It keeps track of the progress of the learning algorithm that was used to identify each feature subset. In this work, the selected features are investigated using linear discriminant analysis (LDA).

i. *Data set* Table 3 summarized the data set used in this research work. The considered data set belongs to 141 different malware families.
ii. *Normalization of data* By using the Min-max normalizing approach, all features are normalized between the ranges of 0 and 1.
iii. *Partition of data* We examined at the data set that wasn’t used for training in order to evaluate the proposed feature selection approach. Further, the data set is divided into two different parts one part is used for training, and the remaining is used for testing. The group ratios in the training and testing of the data sets are nearly identical.
iv. *Filter approach* Pre-processing is the term that describes this technique because it eliminates extraneous features. In this step, the *t*-test and ULR analysis are implemented.
  - *t-test analysis* It examine the statistical significance of benign and malware apps using the *t*-test method. In a 2-class problem (malware apps and benign apps), analysis of the null hypothesis (H0) significant that the two populations are not equal, or it is seen that there is a noticeable variance among their mean values and features used by both of them are different^95^. Furthermore, it shows that the features affect the malware detection result. Hence, those features are considered, which have significant differences in their mean values, and others are excluded. Hence, it is essential to approve the null hypothesis (i.e., H0) and discard the alternative ones^95^. *t*-test is implemented on each of the attributes and then *P* value for each feature is calculated, which indicates how well it distinguishes the group of apps. According to research by^95^, features with an *P* value of < 0.05 show significant biases.
  - *Univariate logistic regression (ULR) analysis* After identifying features that make a significant difference between malware and benign apps, binary ULR analysis is implemented to test the correlation among features that helps in malware detection^95^. ULR analysis is implemented on each selected feature set, which helps in discovering whether the above-selected features were essential to detect the malware-infected apps or not. Only those features are considered, which are having *P* value < 0.05. From the results of the ULR analysis and *t*-test, the hypothesis are rejected and accepted mentioned in Table 5.
v. *Wrapper approach* To determine optimum sets of the feature, cross-correlation analysis and multivariate linear regression stepwise forward selection is implemented in this stage.
  - *Cross correlation analysis* After finding the important features, the correlation analysis is implemented and then examination for both negative and positive correlation coefficients (i.e., r-value) between features is performed. If a feature has a value of r > = 0.7 or r-value < =0.7 with other features, i.e., have a higher correlation then the performance of these features is studied separately. Further, those features are selected, which perform better.
  - *Multivariate linear regression stepwise forward selection* It is not imply that, features that are achieved are relevant to develop malware detection framework. In this stage, ten-fold cross-validation technique is applied to determine the significant features.
vi. *Performance evaluation* Further, to validate that proposed framework is able to identify malware-infected apps that were developed by implementing the steps mentioned above by using independent test data. Additionally, the efficiency of the essential feature sets used for malware detection is validated. On thirty different categories of Android apps, nine different machine learning classifiers were used to develop the investigation model. To evaluate the framework two separate performance parameters, are considered i.e., F-measure and Accuracy. The effectiveness of our detection model is then evaluated using the proposed malware detection methodology.

### Evaluation of proposed framework

Three different approaches are used to evaluate our proposed framework:

- *Comparison with previously used classifiers* Parameters like Accuracy and F-measure are compared with existing classifiers proposed by researchers in the literature to see if our suggested model is feasible or not.
- *Comparison with AV scanners* To compare the effectiveness of our suggested work, ten different anti-virus scanners are considered and their performance is evaluated on the collected data set.
- *Detection of unknown and known malware families* The proposed framework is also examined to see whether it can identify known and unknown malware families.

## Experimental setup and results

**Figure 6:**
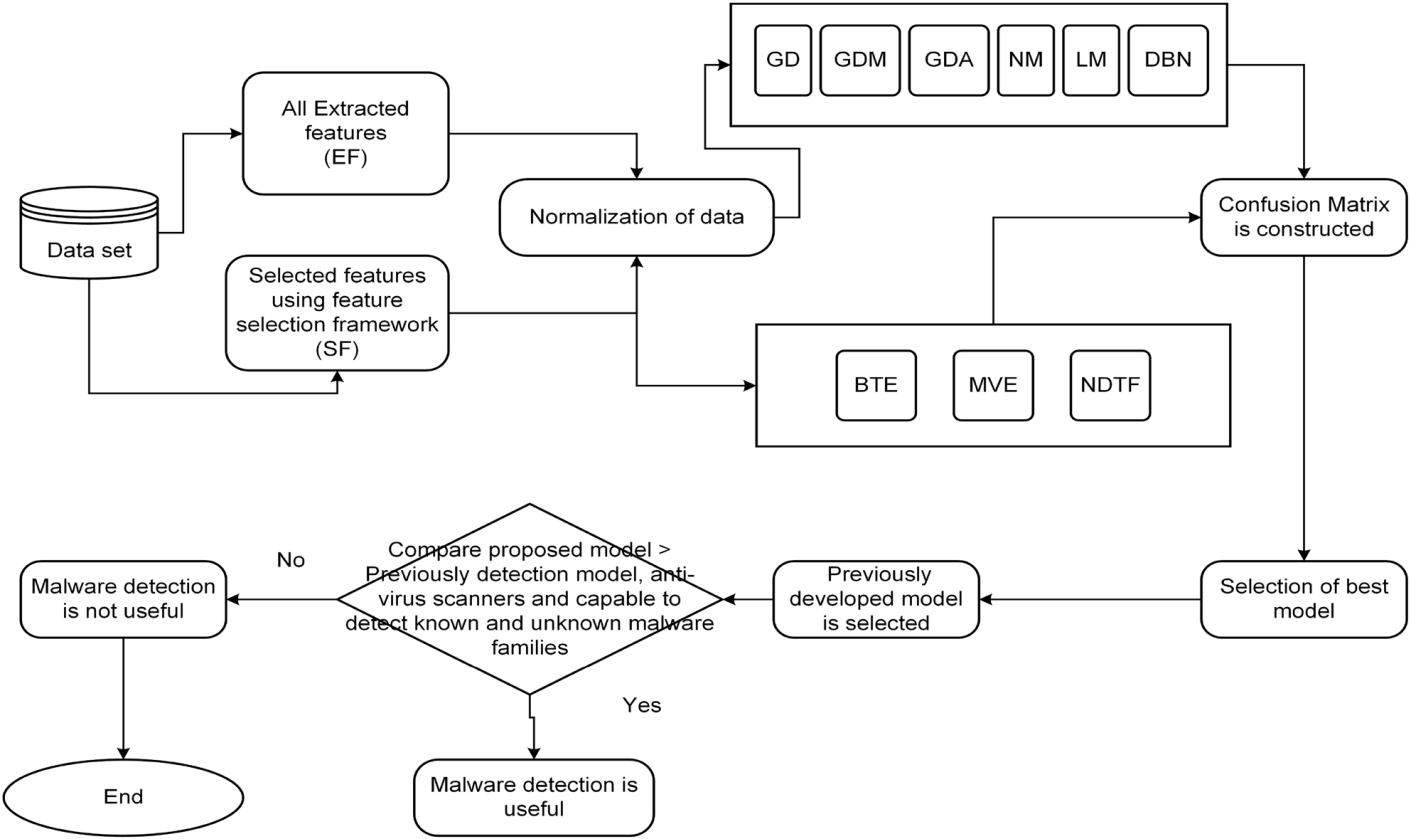
Proposed framework i.e., PermDroid.

The experimental setting used to develop the malware detection model is described in this portion of the paper. The model is developed using a Neural Network (NN) using six different types of machine learning algorithms, namely GD, NM, LM, GDA, GDM, DNN, and three ensemble techniques, including the best training, non-linear decision tree forest, and majority voting. These algorithms are applied on Android apps that were collected from different resources. Each category has a distinct number of benign and malicious apps (they are further separated into various families), which is sufficient for our analysis. Figure 6 presents PermDroid, our suggested framework.

Following are the phases that are pursued in this study, to develop an effective and efficient malware detection framework. The proposed feature selection framework is applied to all the extracted feature data sets, to select significant features. After that, six different machine learning algorithms based on the principle of neural network and three different ensemble algorithms are considered to develop a malware detection model. So, in this study, a total of 540 (30 different Android apps data sets * 9 different machine learning techniques * (one takes into account all extracted features, and another takes into account features identified using the suggested feature selection framework. )) different detection models are developed. The following are a detailed description of the model followed in this study:

1. Thirty different extracted feature data sets are used to implement the proposed feature selection framework.
2. The first stage, which involved identifying significant features, was employed as an input to train the model using various classification and ensemble machine learning approaches. In this research paper, ten-fold cross-validation technique is implemented to verify the develop model^16^. Further, outliers are eliminated, which effect the performance of the proposed framework. The performance of outliers is measured using the equation below:
  13 $$\begin{aligned} e_i=\left\{ \begin{array}{ll} \text{ if } |z_{ji}-\hat{z_j}|>3 * \sigma \text{ for } \text{ Effective } \text{ outliers, } \\ \text{ if } |z_{ji}-\hat{z_j}|\le 3 * \sigma \text{ for } \text{ Non } \text{ Effective } \text{ outliers } \end{array}\right. \end{aligned}$$
3. The developed model using the aforementioned two processes is evaluated using the collected data set in order to determine whether or not the proposed framework is successful in identifying malicious apps.

### Validation of the proposed feature selection framework

In this subsection, the selection of significant feature sets for malware detection is explained. Our analysis is started by using thirty different feature sets (mentioned in Table 4).

#### t-Test analysis

**Figure 7:**
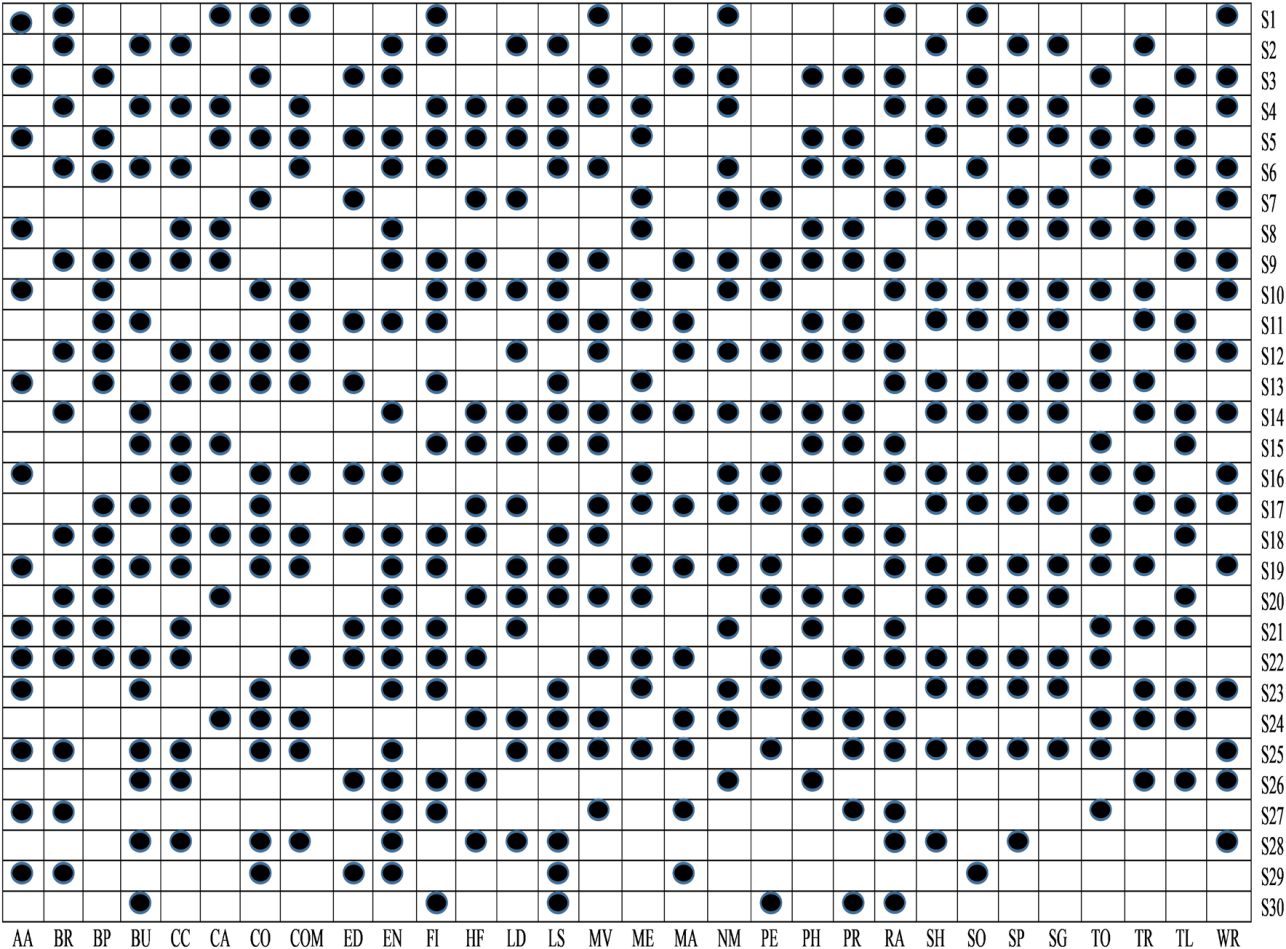
*t*-Test analysis.

*t*-test analysis is used to determine the statistical significance of detecting the malware from Android apps. In this work, *t*-test is applied on extracted feature sets and calculated its *P* value. Further, in this study, the cut-off *P* value considered is 0.05, i.e., it denotes that feature sets that have *P* value < 0.05 has a strong prediction capability. Figure 7 illustrates the findings of a *t*-test performed on the thirty various categories of Android apps that comprise up our obtained data set. The *P* value is provided using two forms for simplicity of use (box with black circle $(\cdot)$ means *P* value < 0.05 and blank box $_ \Box$ means *P* value > than 0.05). The sets of features with emphasis *P* values of < 0.05 have a significant impact on identifying malicious or benign apps. Figure 7 shows how the S29, S27, S25, S23, S22, S21, S19, S18, S13, S10, S8, S5, S3, and S1 feature sets might help to detect malicious and benign apps in the Arcade and Action categories. As a result, in this study, we rule out the hypotheses H1, H3, H5, H8, H10, H13, H18, H19, H21, H22, H23, H25, H27, and H29, coming to the conclusion that these sets of features are capable of identifying apps in the Arcade and Action category that are malicious or benign.

**Figure 8:**
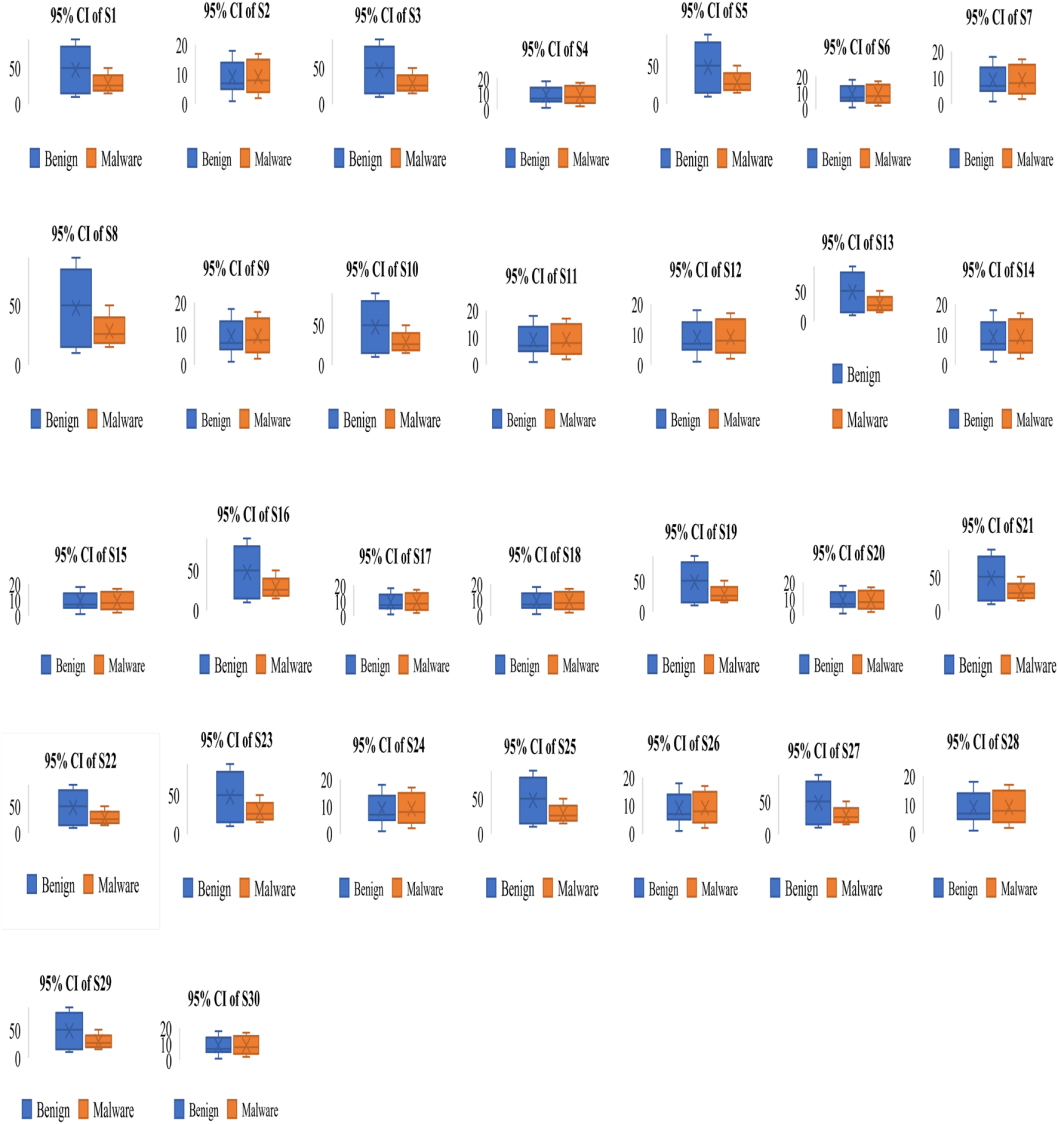
Error box-plots for all the set of permissions in Arcade and Action category apps.

To understand the relationship between malware and benign apps, we have drawn an error box-plot diagram. These box-plot diagrams verify the outcomes of the *t*-test analysis. If there is no overlapping in means and their confidence intervals (CI), then it means there will be a statistical difference between malware and benign apps else. There is no significant difference between them. An error box-plot of the 95% confidence intervals throughout the sets of features and the mean for Arcade and Action category apps is demonstrated in Fig. 8. The outcomes of other categories of Android apps are of similar types. Based on Fig. 8, we can observe that the boxes of S29, S27, S25, S23, S22, S21, S19, S18, S13, S10, S8, S5, S3, and S1 sets of feature do not overlap which means they are significantly different from each other. The mean value of the malware group is higher than the benign group apps. Based on error box-plots, we consider the hypotheses H1, H3, H5, H8, H10, H13, H18, H19, H21, H22, H23, H25, H27 and H29 concluding that these feature sets can able to identify the malware-infected apps for Arcade and Action category Android apps.

#### ULR analysis

**Figure 9:**
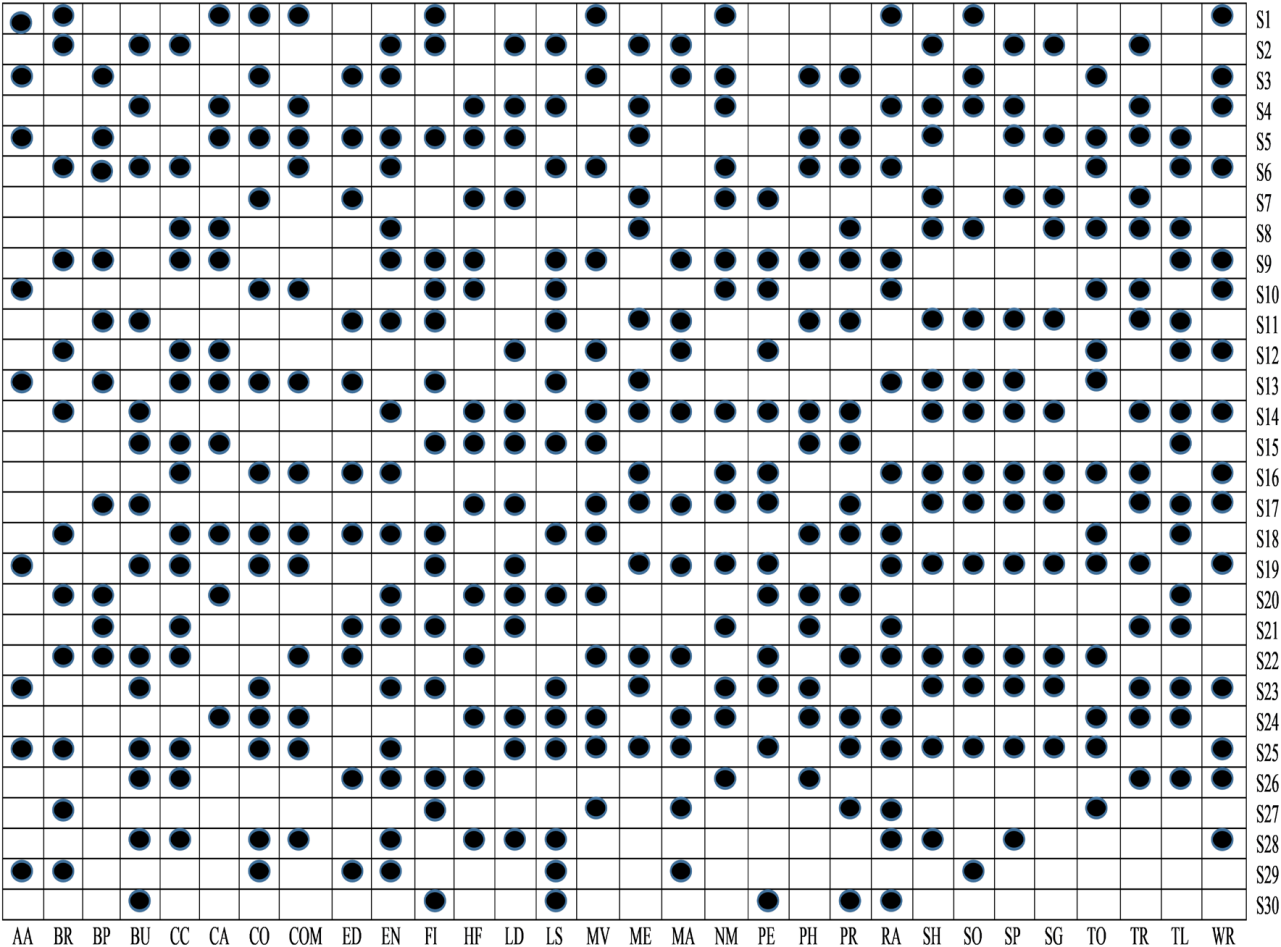
ULR analysis.

To examine whether the selected sets of feature after implementing *t*-test analysis are significant to identify malware apps or not, in this study, ULR analysis is performed on selected sets of features. A set of features is considerably associated with malware detection if its *P* value is < 0.05. In every task, some sets of features are essential for the evolution of the malware detection model, while different sets of features do not seem to be appropriate for malware detection. The outcomes of the ULR approach are demonstrated in Fig. 9. Equivalent to t-test analysis, the same representation is used as such in *P* values, i.e., blank box means *P* value > 0.05 and box having black square has *P* value $\le$ to 0.05.

**Figure 10:**
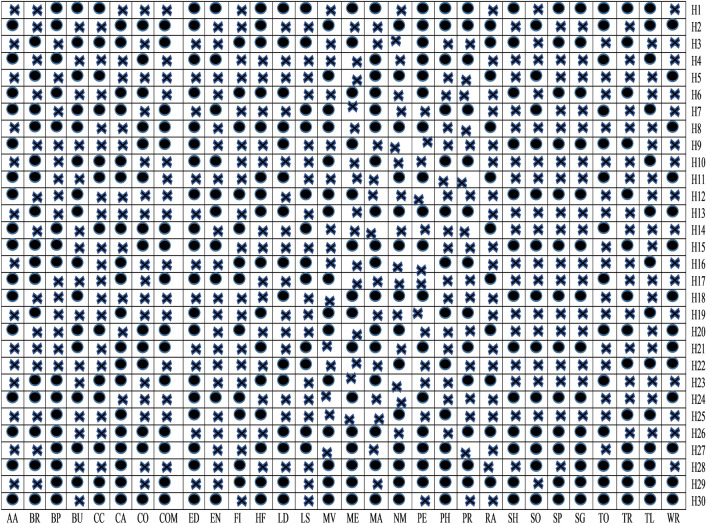
Hypothesis.

From Fig. 9, it is clear that among thirty different categories of features, only S5, S3, S1, S13, S10, S23, S19, S29, and S25 sets of features are significant detectors of malware apps. As a result, we reject null hypotheses H1, H3, H5, H10, H13, H19, H23, H25, and H29 and conclude that these sets of features are directly related to the functioning of the apps. After implementing *t*-test and ULR analysis on our collected sets of features, rejection and acceptance of the hypotheses is done that is presented in the Table 5. Figure 10 demonstrates the rejection and acceptance of the hypotheses for all of the thirty different categories of Android apps. The horizontal and vertical axes indicate the name of the hypothesis and the equivalent category of the Android app, accordingly. To represent the rejection and acceptance of the hypotheses, the cross symbol $(\times )$ and black circle $(\cdot )$, are used respectively. Based on Fig. 10, it is observed that only sixteen hypotheses out of thirty are accepted. Others are rejected for Arcade and Action category Android apps.

#### Cross correlation analysis

**Figure 11:**
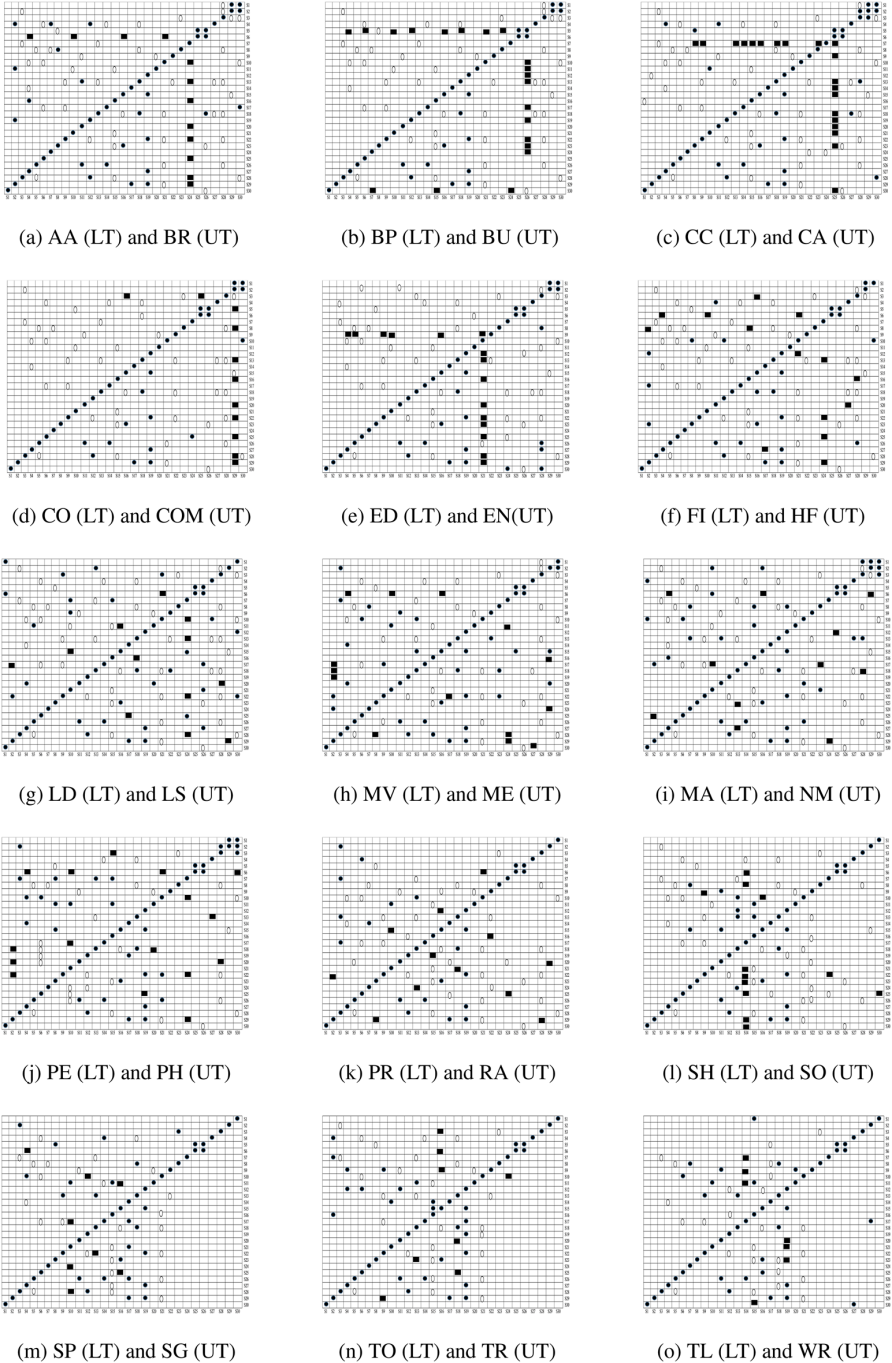
Correlation between set of features (here LT stands for lower triangle and UT stands for Upper triangle.

**Figure 12:**
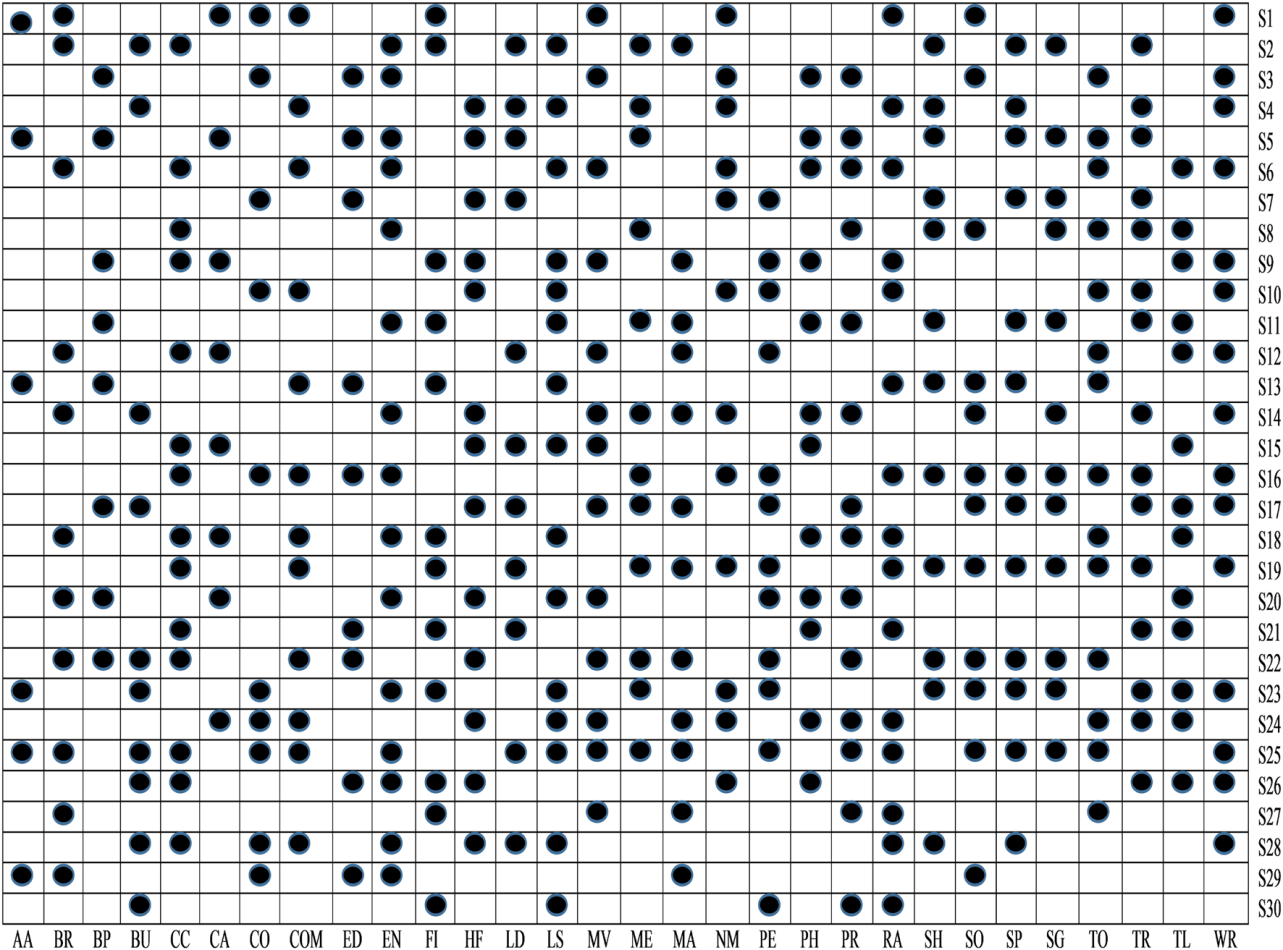
Features selected after implementing cross correlation analysis.

Figure 11 demonstrates the Pearson’s correlation between sets of features for all the categories of Android apps. The lower triangular (LT) and upper triangular (UT) matrices indicate the correlation in different sets of features for distinct Android app categories. The linear relation is evaluated by using the value of the correlation coefficient between distinct sets of extracted features from Android apps. In the present paper, Pearson’s correlation (r: Coefficient of correlation) is used to determine the linear relationship among distinct sets of features. The direction of the association is determined by whether the correlation coefficient, *r*, has a positive or negative sign. If the value of *r* is positive, it indicates that dependent and independent variables grow linearly or if the value of *r* is negative. Both the dependent and independent variables are inversely proportional to each other. Cross-correlation analysis is conducted only on the sets of features that were identified by implemented ULR and *t*-test analysis. If the relevant sets of features show a higher value of correlation (i.e.,*r*-value $\ge$ 0.7 or *r*-value $\le -0.7$) with pertinent other sets of features, then the performance of these sets of feature separately and on the joint basis for malware detection is validated and consider those sets of feature which perform well. Figure 12 demonstrates the selected sets of the feature after implementing cross-correlation analysis. The selected sets of features are represented by utilizing a black circle $(\cdot)$, demonstrating that equivalent sets of features are considered for this research paper.

#### Stepwise forward selection for multivariate linear regression

**Figure 13:**
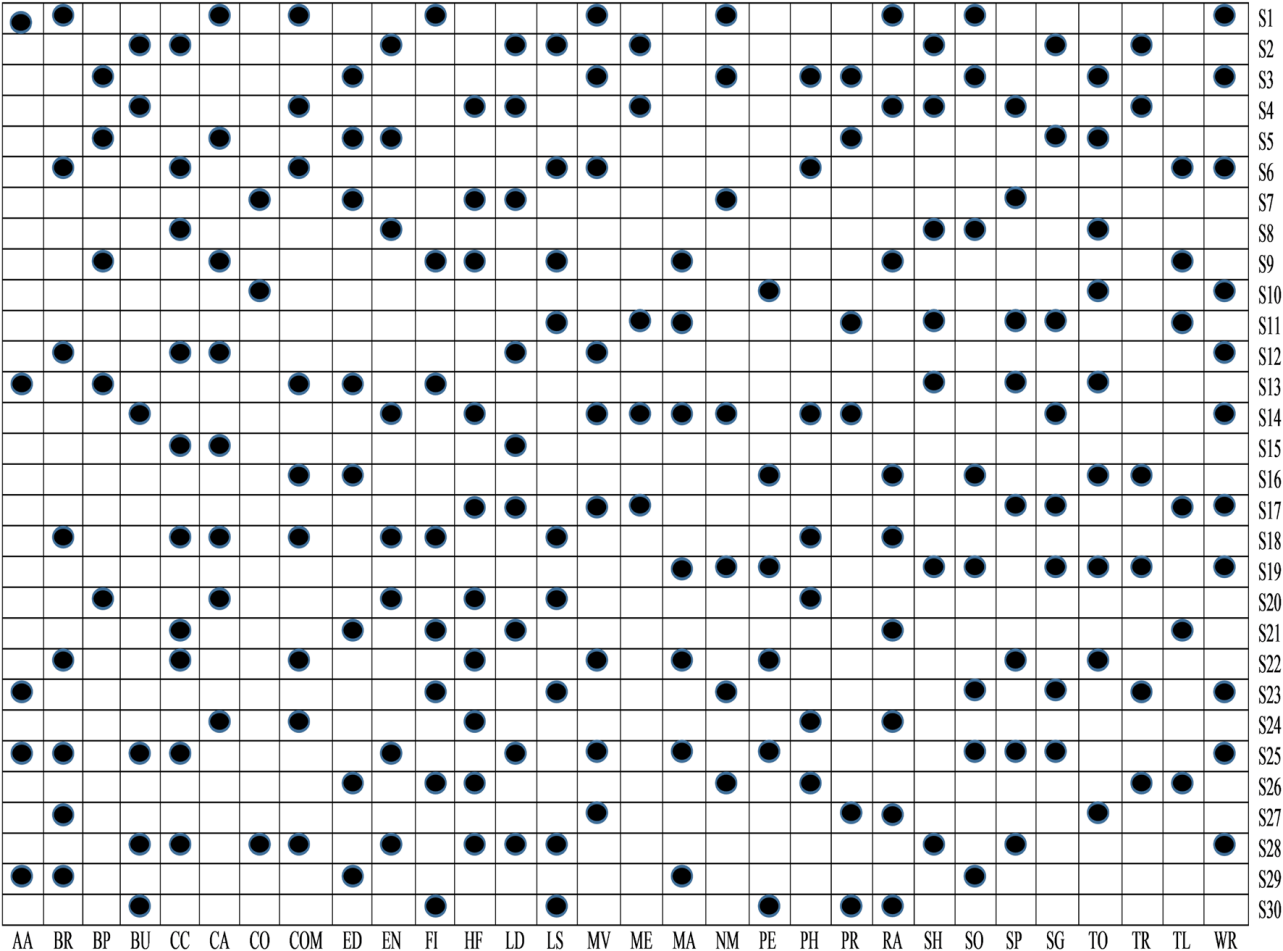
Features selected after implementing multivariate linear regression stepwise forward selection.

After using cross-correlation analysis, the selected subset of features may or may not be important for creating the malware detection model. Further, a multivariate linear regression stepwise forward selection method is implemented in this study to discover the most important features for creating Android malware detection models. After applying multivariate linear regression stepwise on the retrieved feature data set, Fig. 13 shows a significant set of features. A set of features that were taken into account in this paper while building a malware detection model is represented by a black circle with the symbol $(\cdot )$.

**Figure 14:**
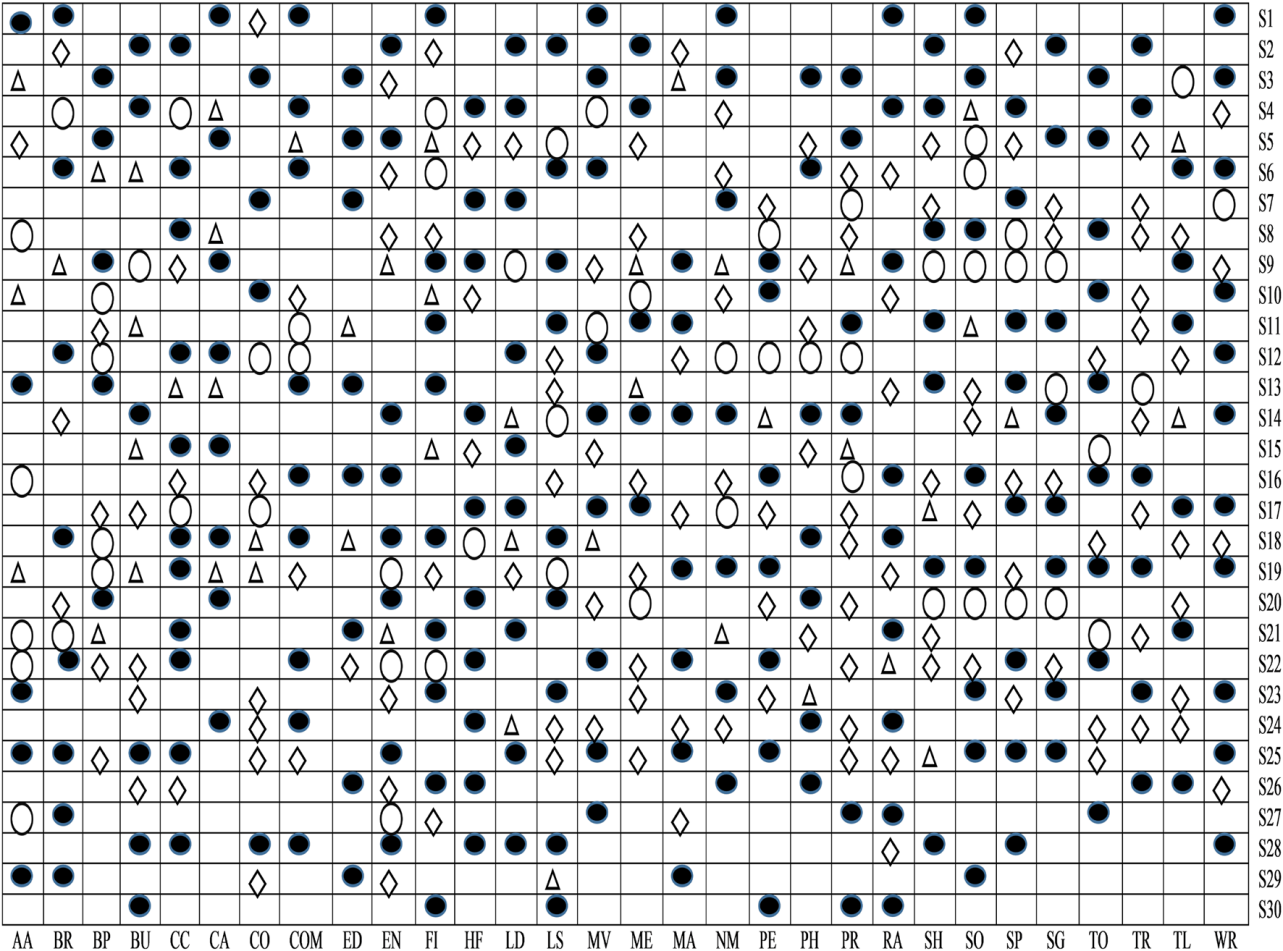
Selected sets of feature for malware detection.

**Figure 15:**
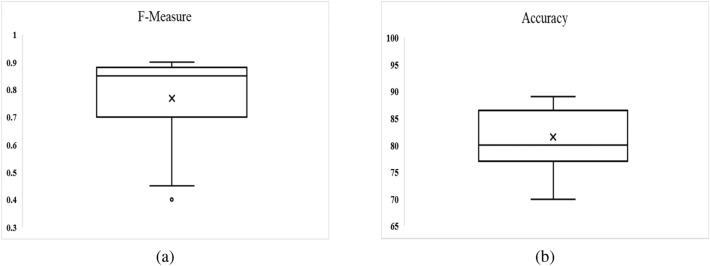
Results of testing data by considering performance parameters.

#### The overall outcome of the feature selection method

In this study, four distinct phases are used to identify relevant sets of features that will be taken into account while constructing the Android malware detection model. Some relevant sets of features are identified from the available sets of features in each stage based on the outcomes of the intermediate analysis. A selection of features from each of the thirty various categories of Android apps are shown in Fig. 14. To make things easier, the selected feature sets are represented by four separate characters, as shown below:

- Empty circle symbol: Features are relevant after implementing *t*-test analysis.
- Triangle symbol: Features are relevant after implementing ULR analysis and *t*-test.
- Diamond symbol: Features are relevant after applied cross-correlation analysis, ULR, and *t*-test.
- Filled circle symbol: Features are relevant after implementing multivariate linear regression stepwise forward selection method, cross-correlation analysis, ULR, and *t*-test.

#### Evaluation on the basis of performance parameters

To examine set of features, a new data set is used that was not previously considered in this study. The model is originally built using ten-fold cross-validation, multivariate linear regression, and selected feature sets as input. Figure 15 illustrates the box-plot diagram for performance measures for all Android apps categories used in this study, including F-measure and Accuracy. It reveals that the outcome is computed as Accuracy of 82 percent and an average F-measure of 0.80.

### Evaluation of the malware detection models developed using ANN

In this paper, we use a neural network to develop a model for malware detection using six different types of machine learning algorithms.

Two separate feature data sets are used as input to construct a model for identifying malware from Android apps (one comprises all extracted features (EF) and the other is used using the feature selection framework (SF). The following hardware was used to complete this task: a Core i7 processor with a 1 TB hard disc and 64 GB RAM. Each malware detection model’s performance is measured using two performance parameters: F-Measure and Accuracy. The outcomes of using a neural network with six different machine learning techniques to achieve performance metrics for various categories of Android apps are shown in Tables 8 and 9. From Tables 8 and 9, the following conclusions can be drawn:

- The model developed by features selected using proposed framework (Model also developed by using distinct feature selection approaches are shown in Tables S1 to S14 in “Online Appendix A”) as an input produces better results when compared to a model constructed by taking into account all sets of features, presenting a significant value of F-measure and Accuracy for identifying malware.
- In compared to the others, the neural network with Deep Neural Network (DNN) training method yields higher outcomes.

**Figure 16:**
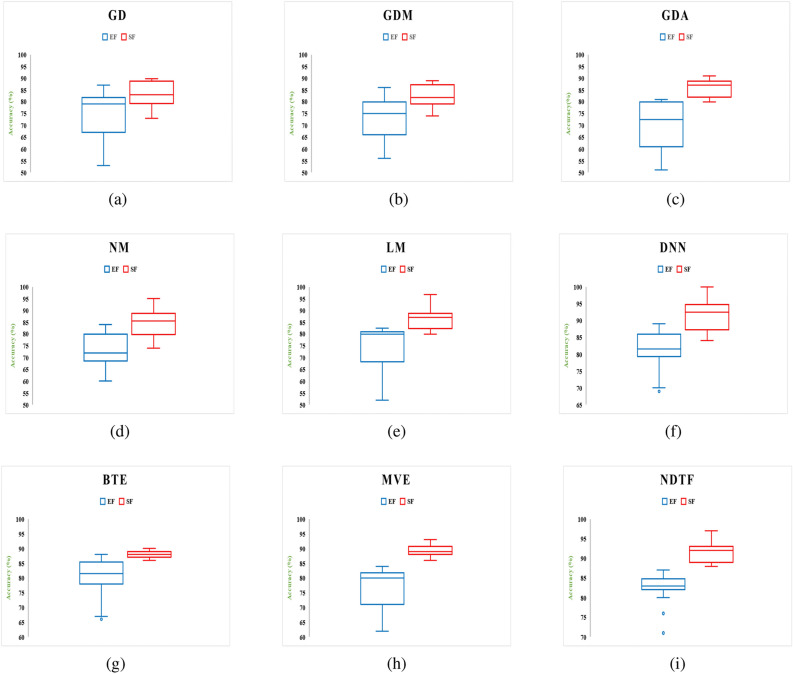
Box-plot diagram for measured performance parameter i.e., Accuracy.

**Figure 17:**
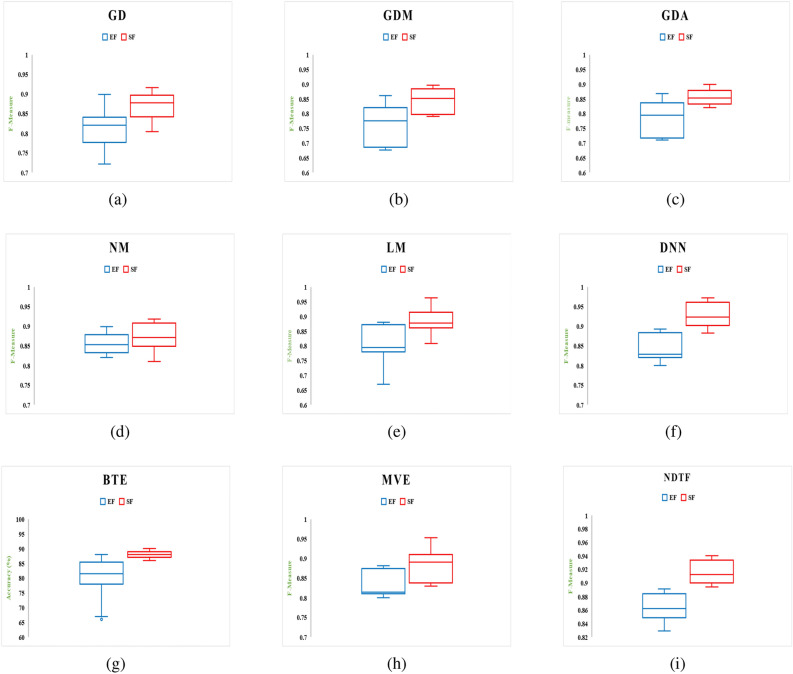
Box-plot diagram for measured performance parameter i.e., F-measure.

Figures 16 and 17 show the Accuracy and F-measure box-plot diagrams for each model built using classification methods. Each figure has two box plots, one containing all of the extracted features (EF) and the other containing only selected feature sets (SF).

The Box-plot diagram assists us in analyzing the performance of all the implemented approaches based on a single diagram. The line drawn in the middle of each box-plot diagram, i.e. the median, is used to determine its value. If a model’s median value is high, it’s regarded as the best model for detecting malware. It can be inferred from Figs. 16 and 17 that:

- The models developed utilizing a significant set of features have high median values. The box-plot diagrams in Figs. 16 and 17 show that SF outperformed all extracted features in terms of detecting Android malware.
- The DNN-based model yields the best results out of all the machine learning techniques for classification that have been used.

**Table 8:** Accuracy.

ID	GD		GDM		GDA		NM		LM		DNN		BTE		MVE		NDTF	
	EF	SF	EF	SF	EF	SF	EF	SF	EF	SF	EF	SF	EF	SF	EF	SF	EF	SF
D1	82.4	84.8	76	81.6	77.6	82.4	84	88	82.4	**93.671**	85	89.5	80.8	88	79.2	89.2	84.8	89.6
D2	83	89.7	79.81	84.730	78	83	73	79.61	82	89.6	89	**93.723**	81	86	87	93	83	89
D3	87	89	76	79.912	81	86	80	85	82	86	89	**94.119**	76	80	82	86	86	90
D4	83	88	76	79	82	88	81	86	80	83	86	89.1	76	80	81	83	84	**90**
D5	80	83	72	76.9	82	86	78	82	80.3	86.8	84	89.1	76	82	83	87	88	**95**
D6	80	83	81	89.9	76	82	68	74	80	84	82	**93.1**	76	79.9	81	84	83	88
D7	77	79	83	**89.9**	81	86	70	75	81	89	80	88	72	76	80	83	82	88
D8	67	73	76	79.9	82	89	84	89	83	89	88	**96.189**	72	78	82	88	86	90.321
D9	77	82	74	79.9	80	84	81	87	83	88	88	95	81	86	83	90	89	**96**
D10	77	82	72	79.9	84	89	81	89	72	86	84	** 94.114**	76	88	81	89	88	94
D11	56	79	56	79.9	51	82	70	85	62	86	79	84.1	71	82	67	86	86	**96**
D12	78	89	77	82.913	71	88	70	84	72	86	79	88.121	72	80	62	86	76	**90.910**
D13	53	79	56	69.912	71	86	76	85	72	86	79	**94.1**	78	83	80	86	81	92
D14	57	82	76	89.988	74	82	68	79	57	81	78	**84.1**	72	80	67	78	71	80
D15	81	86	86	89.961	61	86	70	85	82	86	81	94.131	66	80	84	86	88	**98**
D16	67	79	66	79.932	81	86	70	85.8	88.1	**96.7**	81	92.1	66	80	82	86	81	92
D17	57	78	76	82.912	80	91	80	85	72	86	79	**93**	76	84	72	91	76	92
D18	67	82	76	82.114	74	86	68	85	62	86	69	84	66	80	62	86	66	**89**
D19	71	89	66	89.914	61	81	76	85	80	86	82	96.111	71	82	80	88	82	**97**
D20	47	78	67	71.621	61	82	70	82	80	89	78	**92.133**	67	84	80	88	82	91
D21	67	81	72	89.9	80	88	78	82	80	89	81	**97.112**	86	89	71	86	76	88
D22	77	82	77	89	61	86	72	80	81	88	81	92	66	82	62	86	66	**92.96**
D23	57	78	56	79	51	76	78	89	52	76	79	90	70	88	72	86	76	**93**
D24	57	80	76	89.9	81	86	80	89	78	86	89	**100**	76	88	82	92	86	97
D25	78	89	76	88	68	86	70	82	80	89	82	**97**	81	93	82	91	86	95
D26	70	82	76	88	80	91	60	75	62	86	89	**94**	56	70	52	76	66	90
D27	77	89	66	79.432	80	88	80	88	80	88	82	95	70	88	72	89	80	**97**
D28	67	89	61	74	81	91	80	95	82	96	89	**99.1**	56	81	62	81.78	66	93.78
D29	77	82.67	71	79.912	71	86.67	80.77	85.98	82.778	89.897	81	96.1	76	80.8	82	89.99	81	**96.77**
D30	81	89	62	79.9	81	86	80	85	82	86	89	**94.1**	71	80	82	86	86	90

Significance of values are in bold.

**Table 9:** F-Measure.

ID	GD		GDM		GDA		NM		LM		DNN		BTE		MVE		NDTF	
	EF	SF	EF	SF	EF	SF	EF	SF	EF	SF	EF	SF	EF	SF	EF	SF	EF	SF
D1	0.899	0.916	0.861	0.896	0.868	0.899	0.908	0.932	0.879	**0.9632**	0.88	0.921	0.889	0.929	0.882	0.953	0.8914	0.934
D2	0.822	0.864	0.789	0.851	0.794	0.837	0.762	0.881	0.77	**0.901**	0.8289	0.902	0.8182	0.8953	0.814	0.904	0.8814	0.9234
D3	0.777	0.891	0.676	0.79912	0.71	0.82	0.78	0.80	0.78	0.8612	0.8932	**0.923**	0.762	0.881	0.81	0.891	0.8622	0.901
D4	0.82	0.8421	0.762	0.7911	0.72	0.872	0.71	0.8622	0.80	0.877	0.82	0.8822	0.77	0.880	0.81	0.838	0.8488	**0.90**
D5	0.70	0.822	0.712	0.706	0.72	0.862	0.778	0.821	0.803	0.828	0.81	0.881	0.66	0.8222	0.83	0.57	0.78	**0.933**
D6	0.577	0.7822	0.671	0.871	0.761	0.811	0.68	0.712	0.801	0.8224	0.802	**0.9133**	0.767	0.799	0.821	0.884	0.8223	0.8568
D7	0.57	0.79	0.63	**0.899**	0.66	0.76	0.70	0.77	0.8188	0.889	0.703	0.788	0.72	0.767	0.8033	0.8321	0.782	0.788
D8	0.578	0.7711	0.710	0.799	0.8222	0.873	0.82	0.87	0.82	0.87	0.86	**0.912**	0.67	0.78	0.821	0.871	0.851	0.89321
D9	0.67	0.72	0.788	0.899	0.801	0.821	0.811	0.872	0.63	0.78	0.58	0.85	0.61	0.76	0.723	0.8099	0.889	**0.946**
D10	0.772	0.812	0.712	0.799	0.74	0.829	0.811	0.869	0.712	0.836	0.814	** 0.924**	0.7621	0.8211	0.812	0.849	0.828	0.90
D11	0.562	0.739	0.676	0.799	0.551	0.782	0.701	0.825	0.612	0.826	0.749	0.841	0.717	0.8152	0.617	0.816	0.836	**0.929**
D12	0.78	0.819	0.672	0.813	0.711	0.86	0.703	0.814	0.722	0.816	0.69	0.8	0.701	0.82	0.6122	0.863	0.656	**0.8910**
D13	0.53	0.719	0.526	0.6912	0.7611	0.8026	0.56	0.825	0.702	0.816	0.729	**0.884**	0.7381	0.82	0.709	0.806	0.811	0.871
D14	0.59	0.72	0.761	0.880	0.67	0.82	0.578	0.789	0.507	0.781	0.88	**0.92**	0.67	0.822	0.67	0.728	0.721	0.7880
D15	0.811	0.856	0.761	0.8961	0.621	0.861	0.75	0.825	0.82	0.876	0.801	0.931	0.66	0.820	0.780	0.8686	0.818	**0.92**
D16	0.57	0.709	0.63	0.792	0.71	0.806	0.710	0.88	0.861	**0.907**	0.821	0.89	0.56	0.810	0.812	0.856	0.801	0.912
D17	0.557	0.768	0.736	0.891	0.810	0.901	0.802	0.845	0.722	0.846	0.769	**0.91**	0.716	0.814	0.762	0.891	0.726	0.892
D18	0.767	0.812	0.706	0.84	0.714	0.816	0.648	0.825	0.672	0.876	0.692	0.824	0.626	0.810	0.62	0.816	0.566	**0.86**
D19	0.671	0.809	0.656	0.824	0.601	0.781	0.726	0.825	0.780	0.826	0.682	0.86	0.701	0.872	0.780	0.818	0.872	**0.917**
D20	0.487	0.768	0.647	0.7621	0.618	0.782	0.67	0.782	0.780	0.869	0.718	**0.89**	0.617	0.814	0.780	0.82	0.802	0.8891
D21	0.617	0.802	0.712	0.829	0.780	0.81	0.678	0.782	0.780	0.819	0.681	**0.912**	0.76	0.89	0.711	0.806	0.716	0.838
D22	0.677	0.782	0.577	0.789	0.661	0.786	0.872	0.890	0.71	0.81	0.80	0.89	0.56	0.72	0.60	0.76	0.76	**0.91**
D23	0.657	0.718	0.656	0.719	0.651	0.876	0.718	0.809	0.52	0.716	0.719	0.890	0.670	0.788	0.802	0.861	0.716	**0.893**
D24	0.517	0.780	0.676	0.78	0.781	0.86	0.780	0.889	0.678	0.816	0.809	**0.99**	0.716	0.808	0.802	0.902	0.816	0.967
D25	0.812	0.879	0.716	0.818	0.628	0.856	0.710	0.812	0.780	0.859	0.812	**0.94**	0.801	0.903	0.812	0.891	0.76	0.895
D26	0.70	0.812	0.71	0.88	0.70	0.89	0.63	0.715	0.562	0.786	0.789	**0.9**	0.656	0.770	0.52	0.876	0.56	0.89
D27	0.67	0.88	0.66	0.802	0.80	0.878	0.810	0.889	0.780	0.868	0.812	0.895	0.70	0.88	0.712	0.869	0.780	**0.95**
D28	0.77	0.89	0.601	0.774	0.81	0.891	0.780	0.895	0.802	0.8926	0.819	**0.90**	0.61	0.81	0.612	0.88	0.62	0.899
D29	0.57	0.82	0.671	0.792	0.701	0.87	0.77	0.81	0.778	0.897	0.71	0.86	0.76	0.88	0.82	0.89	0.801	**0.96**
D30	0.71	0.82	0.46	0.69	0.71	0.76	0.780	0.845	0.812	0.856	0.829	**0.91**	0.701	0.880	0.812	0.88	0.66	0.890

Significance of values are in bold.

**Table 10:** To examine the rank test findings, the Wilcoxon signed technique was applied.

Accuracy									F-measure									
	GD	GDM	GDX	NM	LM	DNN	BTE	MVE	NDTF	GD	GDM	GDX	NM	LM	DNN	BTE	MVE	NDTF
(a) Training methods																		
*P* value																		
GD		0.000	0.000	0.000	0.000	0.000	0.000	0.000	0.000		0.001	0.000	0.000	0.000	0.000	0.000	0.000	0.000
GDM			0.425	0.000	0.000	0.000	0.000	0.000	0.000			0.300	0.000	0.000	0.000	0.000	0.000	0.000
GDX				0.000	0.000	0.000	0.000	0.000	0.000				0.000	0.000	0.000	0.000	0.000	0.000
NM					0.000	0.410	0.0220	0.0710	0.050					0.081	0.050	0.0701	0.110	0.107
LM						0.000	0.0120	0.0510	0.020						0.040	0.0601	0.100	0.170
DNN							0.0020	0.0010	0.030							0.0001	0.000	0.450
BTE								0.0040	0.010								0.000	0.020
MVE									0.010									0.010
NDTF																		
Mean																		
GD	0.000	1.508	2.456	− 2.040	− 3.010	− 3.500	− 2.200	− 2.090	− 2.908	0.000	0.001	0.018	− 0.050	− 0.020	− 0.060	− 0.027	− 0.0280	− 0.030
GDM	− 1.006	0.000	1.130	− 4.425	− 4.080	− 5.080	− 3.800	− 4.480	− 4.600	− 0.020	0.000	0.005	− 0.030	− 0.060	− 0.080	− 0.040	− 0.045	− 0.050
GDX	− 2.788	− 1.33	0.000	− 4.000	− 4.213	− 6.170	− 3.120	− 3.780	− 4.230	− 0.011	− 0.002	0.000	− 0.021	− 0.060	− 0.080	− 0.050	− 0.070	− 0.078
NM	2.890	3.899	4.898	0.000	0.890	− 2.410	− 0.620	− 0.0710	− 0.810	0.021	0.034	0.039	0.000	− 0.004	− 0.006	− 0.007	− 0.008	− 0.0015
LM	3.477	5.025	6.671	1.333	0.000	− 1.322	− 0.233	− 0.851	− 0.880	0.020	0.310	0.040	0.300	0.000	− 0.006	− 0.007	− 0.0078	− 0.012
DNN	4.311	4.220	3.780	2.0981	5.678	0000	1.200	2.180	1.910	0.060	0.058	0.052	0.038	0.041	0.000	− 0.001	− 0.003	− 0.005
BTE	2.997	2.633	2.431	2.100	1.890	− 1.988	0.000	− 0.0540	− 0.679	0.028	0.041	0.045	0.007	0.009	0.001	0.000	− 0.002	− 0.020
MVE	2.882	4.488	5.672	0.889	0.998	− 0.533	0.560	0.000	− 0.054	0.028	0.042	0.046	0.002	0.008	0.001	0.002	0.000	− 0.008
NDTF	2.944	4.552	5.661	0.991	0.789	− 0.450	0.646	0.054	0.000	0.036	0.050	0.050	0.015	0.009	0.007	0.008	0.007	0.000

	Accuracy									F-measure								
	Mean		*P* value							Mean		*P* value						
(b) All EF and SF																		
	EF		SF		EF	SF				EF		SF		EF	SF			
EF	0.00		− 5.76			0.00				0.00		− 0.048			0.00			
SF	5.76		0.00							0.048		0.000						

### Evaluation of the malware detection models developed using ensemble techniques

In this study, three different heterogeneous ensemble approaches are considered for creating the Android malware detection model, each with a different combination rule (1 nonlinear and two linear). From Tables 8 and 9 and Figs. 16 and 17, it can be revealed that the NDTF approach outperformed the BTE and MVE approaches. Further, it is also noticed that ensemble approaches detect more malware as compared to other implemented machine learning algorithms except DNN.

### Comparison of the findings

In this study, paired Wilcoxon signed-rank tests to assess the relative performance of several feature sets and machine learning methods is employed. The Wilcoxon test with Bonferroni correction is used in this work for comparative review.

#### On the basis of detection approaches

To create a model that can determine whether an Android app is benign or malicious, nine different classification algorithms were evaluated. Two sets of features have been identified as inputs for developing malware detection models for thirty different categories of Android apps using two different performance parameters, namely F-Measure and Accuracy. One set of features takes into account all extracted features, and the other sets of selected features that are gained by implementing the framework of the feature selection method. Two sets of data are used for each strategy, each having 60 data points ((1 feature selection approach + 1 considering all retrieved features) * 30 Android app categories). The comparisons of pair-wise different machine learning techniques are shown in Table 10.

There are two sections in Table 10. The value of the significant difference between different pairings is shown in the second half of the table, and the calculated *P* value is shown in the first half. Using Bonferroni correction sets, the significant cutoff value is calculated. In this work, nine different machine learning algorithms were examined for creating malware detection models, resulting in a total of 36 potential pairs $^{9 techniques} C_2=36$, with all results examined at a significance threshold of 0.05. We can rule out the null hypothesis if the *P* value is < 0.05/36 = 0.0013. According to the study, the null hypothesis for the test implies that no significant difference exists between the two procedures. Table 10a shows that the *P* value is < 0.0013, indicating that there is a significant difference between the applied processes; out of 36 pairs of training techniques, 22 are offered as a significant outcome. By examining the mean difference value in Table 10a, it can be seen that the DNN method outperformed the performance of other machine learning techniques. In addition, the value of the mean difference of ensemble techniques is better when compared to other models, with the exception of the model built using DNN.

#### On the basis of all selected sets of feature using proposed framework and extracted features

By taking into consideration each set of features, a total of 270 different data points ((3 ensemble techniques + neural network with six machine learning techniques) * 30 types of Android apps) are developed in this study (one for each performance measure). Wilcoxon signed-rank test performance was described in Table 10b. It is seen from Table 10b that there is a significant difference between the models developed because the *P* value is less than 0.05. Additionally, it is evident that the features taken into account employing the feature selection framework outperformed the model developed by using all extracted feature sets when comparing the mean difference values from Table 10b to it.

**Figure 18:**
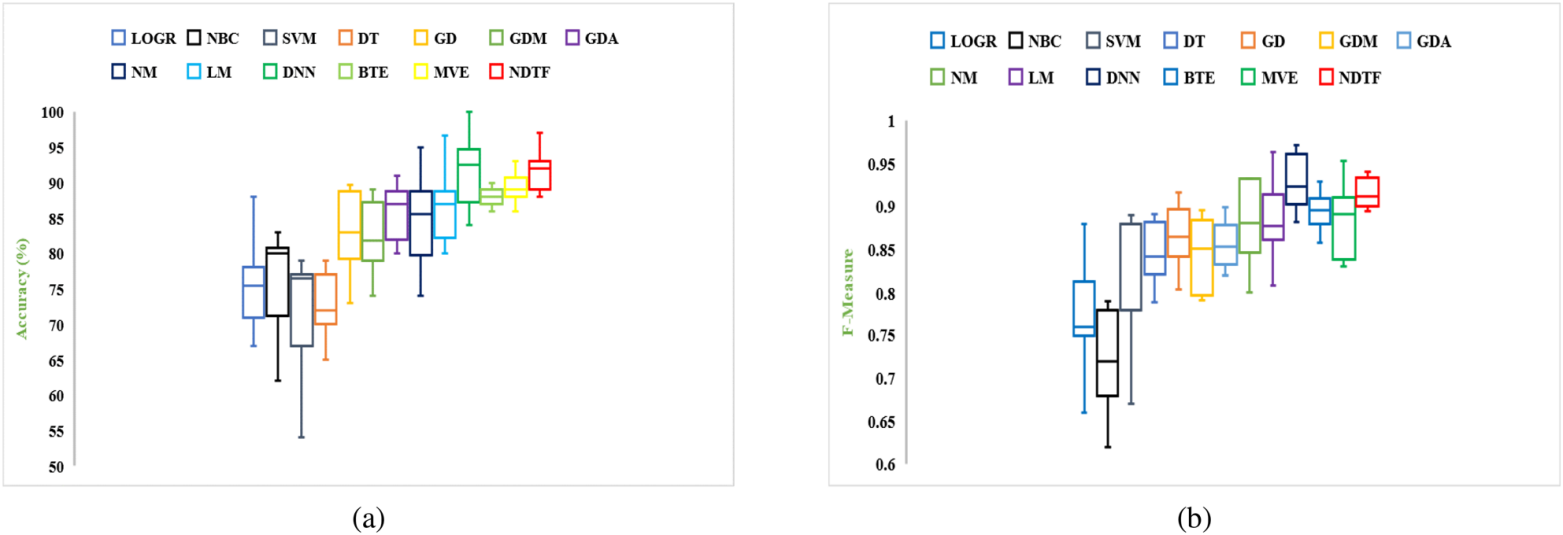
Measured performance parameters i.e., Accuracy and F-measure.

### Proposed framework evaluation

#### Results comparison with previously employed classifiers

In the present study, our newly developed malware detection model is also compared to the models developed using previously used classifiers such as decision tree analysis (DT), support vector machine (SVM), Naïve Bayes classifier (NBC), and logistic regression (LOGR). Two different sets of features (1 considering selected feature sets + 1 using all extracted features) are considered for 30 different categories of Android apps using two independent performance measures i.e., F-Measure and Accuracy. An aggregate of two sets i.e., 60 data points are employed for each classifier model are produced ((1 selected feature sets + 1 considering all extracted features)* 30 data sets). Figure 18 illustrates both the classifiers employed in this study and the most frequently used classifiers in the literature.

On the basis of Fig. 18, it can be seen that the model produced using neural networks has a higher median value and achieves better results than the model developed using the literature’s used classifiers. Further, to decide that, which model produces better results, a pairwise Wilcoxon signed rank test is implemented. Table 11 summarizes the results of the Wilcoxon test with Bonferroni correction examination for accuracy outcomes. Further, the Table 11 is divided into two sections, the first of which indicates the *P* value and the second of which demonstrates the mean difference between different pairs of classifiers. We implemented thirteen different machine learning approaches in this research paper (4 previously applied classifier in the literature + 9 implemented classifier in this study); thus, an aggregate of seventy eight (78) individual pairs are possible $^{13techniques} C_2=78$, and all classifier outcomes are examined at the 0.05 significance level. Only those null hypotheses with an *P* value is less than 0.05/78 = 0.000641 are rejected in this study. Table 11 shows that there is a significant difference between different implemented classifier approaches in a number of cases when the *P* value is less than 0.000641, i.e., 66 out of 78 pairs of classification approaches have significant outcomes. Table 11 demonstrates that the DNN approach outperforms other machine learning classifiers in terms of mean difference value.

#### Using cost-benefit analysis, comparison with previously employed classifiers

A cost-benefit analysis is used to evaluate the performance of developed model. Using the following equation, the cost-benefit analysis for each feature selection strategy is calculated:

**Table 11:** Wilcoxon signed rank test analysis is implemented to the previously used classifier.

Accuracy																										
	*P* value													Mean												
	LOGR	NBC	SVM	DT	GD	GDM	GDX	NM	LM	DNN	BTE	MVE	NDTF	LOGR	NBC	SVM	DT	GD	GDM	GDX	NM	LM	DNN	BTE	MVE	NDTF
LOGR		0.000	0.000	0.160	0.000	0.000	0.000	0.000	0.000	0.000	0.000	0.000	0.000	0.000	2.010	3.940	− 1.30	− 9.087	− 7.890	− 6.67	− 11.890	− 12.880	− 15.880	− 11.77	− 12.99	− 13.00
NBC			0.000	0.000	0.0100	0.670	0.642	0.000	0.000	0.000	0.000	0.000	0.000	8.77	0.000	12.22	7.77	− 1.08	− 1.66	0.08	1.77	− 3.89	− 6.00	− 3.88	− 4.88	− 5.89
SVM				0.000	0.000	0.000	0.000	0.000	0.000	0.000	0.000	0.000	0.000	− 3.89	− 12.89	0.000	− 5.88	− 13.88	− 11.55	− 10.88	− 15.77	− 13.88	− 17.88	− 15.77	− 16.99	− 17.00
DT					0.000	0.000	0.000	0.000	0.000	0.000	0.000	0.000	0.000	1.88	− 7.88	5.88	0.000	− 7.88	− 6.77	− 7.09	− 5.66	− 5.00	− 11.88	− 8.99	− 9.99	− 10.89
GD							0.000	0.000	0.000	0.000	0.000	0.000	0.000	9.88	8.99	12.88	11.20	0.000	1.678	2.77	− 2.06	− 3.88	− 6.05	− 4.55	− 5.08	− 5.89
GDM							0.660	0.000	0.000	0.000	0.000	0.000	0.000	7.88	5.00	6.77	1.99	2.89	0.000	1.110	− 0.89	− 2.88	− 7.88	− 6.77	− 5.77	− 6.990
GDX								0.000	0.000	0.000	0.000	0.000	0.000	6.77	7.08	6.88	7.88	1.99	2.001	0.000	− 1.88	− 2.88	− 8.99	− 6.88	− 7.88	− 8.09
NM									0.000	0.041	0.0020	0.000	0.000	11.99	10.99	15.22	16.77	2.33	1.550	1.7701	0.000	− 1.107	− 11.22	− 8.99	− 7.99	− 10.99
LM									0.000	0.000	0.0080	0.000	0.000	13.79	11.79	14.82	18.67	1.93	1.950	2.701	1.990	0.000	− 13.22	− 7.99	− 9.69	− 12.99
DNN											0.008	0.000	0.004	18.79	17.90	13.33	16.07	6.93	5.050	5.501	4.090	3.780	0.000	1.99	1.69	1.09
BTE												0.003	0.004	12.90	14.90	12.03	15.97	5.73	4.050	3.701	3.801	0.780	− 1.003	0.000	− 0.69	− 0.99
MVE													0.003	10.90	12.890	10.403	13.097	2.73	2.950	2.701	2.201	1.680	− 3.003	− 1.088	0.000	− 1.999
NDTF														17.90	16.890	12.403	15.097	3.631	3.850	2.491	2.881	1.980	− 1.003	0.078	0.070	0.000

14 $$\begin{aligned} Cost-Benefit=(Based_{cost}+Benefit_{cost})/2. \end{aligned}$$

In this case, $Based_{cost}$ is determined by the correlation between the specified features set and the class error. The following equation can be used to compute $Based_{cost}$:

15 $$\begin{aligned} Based_{cost}=Accuracy \ (SM)*\rho _{SM.fault}. \end{aligned}$$

The multiple correlation coefficient between the error and the selected feature set is $\rho _{SM.fault}$ , and the classification accuracy used to build a malware detection model using the selected feature set is $Accuracy \ (SM)$. The proposed model has a greater accuracy and a larger $Based_{cost}$ since it has a higher multiple correlation coefficient. After adopting feature selection procedures, NAM stands for feature sets, while NSM stands for the number of selected features. The following equation can be used to determine $Based_{cost}$:

16 $$\begin{aligned} Based_{cost}=NAM-NSM/NAM \end{aligned}$$

**Figure 19:**
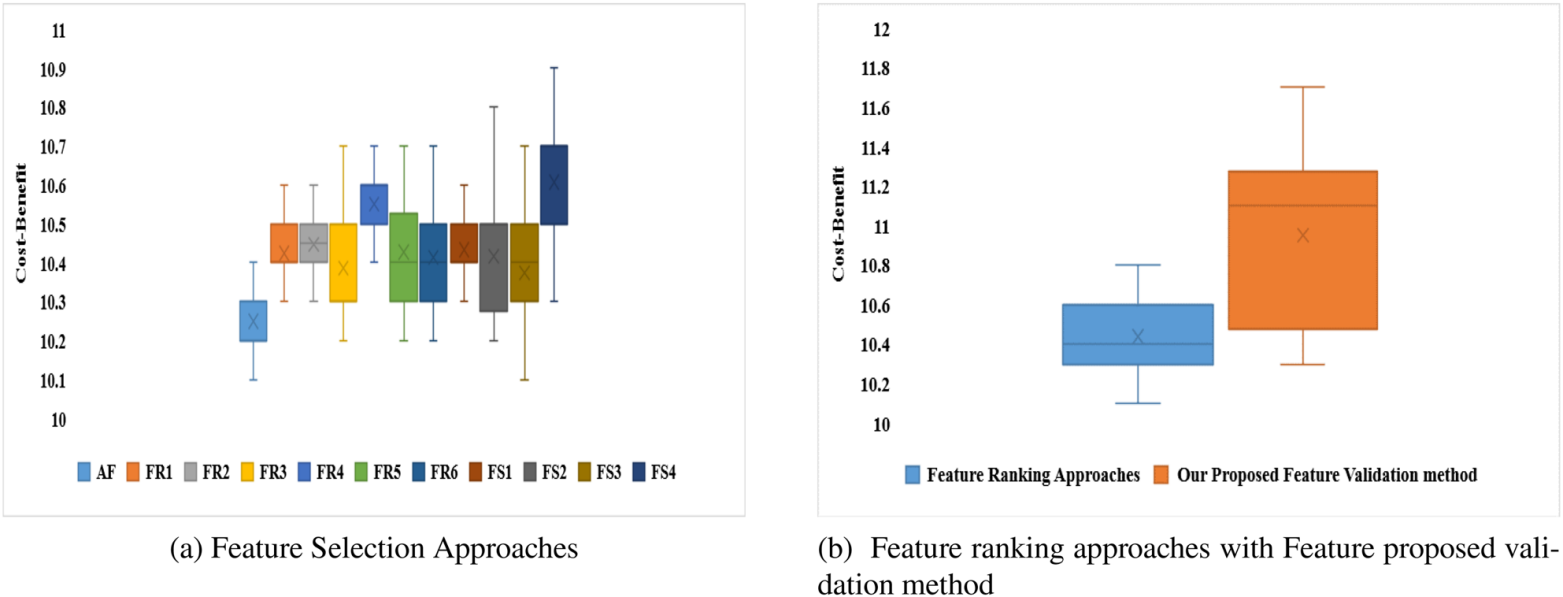
Calculated cost-benefit value.

Instead of using the feature selection validation method, we use six other feature ranking approaches to evaluate PermDroid’s performance in this study. The naming standards used for the experiment are listed in Table 12. The most important feature selection technique, as suggested in^96^, is the one that achieves a better value of cost-benefit. The cost-benefit analysis of different feature selection procedures is shown in Fig. 19a,b. It is discovered that sets of features were selected after applying multivariate linear regression stepwise forward selection technique, cross-correlation analysis, ULR, and *t*-test to achieve a higher median Cost-benefit measure when compared to other feature selection techniques used by researchers in the literature.

In the literature academicians and researchers implemented different feature ranking and feature subset selection approaches i.e., Chi-squared test, Gain-ratio, Information-gain, Principal Component Analysis and Filtered subset evaluation. To evaluate the performance of our proposed feature selection approach, an experiment was performed by using Drebin data set and accuracy is measured and represented in Table 13. Out of implemented six different feature selection techniques our proposed feature selection approach achieved an higher accuracy when compared to others.

**Table 12:** Naming standards used for the experiment.

Abbreviation	Corresponding name
FR1	Chi squared test
FS1	*t*-test
FR2	Gain ratio feature evaluation
FS2	ULR and *t*-test
FR3	Filtered subset evaluation
FS3	ULR, *t*-test and cross correlation analysis
FR4	Information gain feature evaluation
FS4	ULR, *t*-test, multivariate linear regression stepwise forward selection method and cross correlation analysis
AF	All extracted features
FR5	Logistic regression analysis
DS	Data set
FR6	Principal component analysis (PCA)

**Table 13:** Comparison of proposed framework with existing feature selection approaches.

Feature selection technique	Accuracy achieved in percentage
FR1	75
FS1	72
FR2	78.6
FR3	73.3
FR5	72.8
FR6	81.8
Proposed feature selection technique	98.7

#### Comparison of results based on the amount of time it takes to identify malware in real-world apps

In this section of the article, the performance of PermDroid is compared in terms of the time needed to identify malware in real-world apps. For this experiment, we download the data set from two different repositories Drebin (https://www.sec.cs.tu-bs.de/~danarp/drebin/download.html) and AMD (http://amd.arguslab.org/) and experimented by implementing the individual frameworks. Table 14 shows that, when compared to the individual frameworks available in the literature, our suggested technique can identify malware in less time.

#### Comparison of the results on the basis of detection rate with different approaches or frameworks available in the literature

Furthermore, proposed malware detection model (i.e., PermDroid) is compared to previously developed techniques or frameworks present in the literature. The names, methodology, deployment, purpose, data collection, and detection rate of proposed methodologies or frameworks are listed in Table 15. Empirical result revealed that our proposed framework produced a 3 percent greater detection rate. Experiment was performed by using Drebin data set (https://www.sec.cs.tu-bs.de/~danarp/drebin/download.html).

#### Comparison of results with different AV Scanners

Although PermDroid outperforms the classifiers used in the research, it should ultimately be similar to the results obtained using regular anti-virus software in the field for Android malware detection. For this study, ten different anti-virus softwares are selected from the market and used them on the data set that has been gathered in this study.

**Table 14:** Compare PermDroid’s performance to earlier frameworks that have been developed.

Frameworks	TPR	FPR	Average time in identifying app is malicious or not (Sec)
MADAM (2012)^38^	0.88	0.6	1200
DroidScope (2012)^39^	0.89	0.6	1280
AppGuard (2012)^40^	0.87	0.7	1100
TstructDroid (2013)^41^	0.88	0.7	1200
AppsPlayground (2013)^42^	0.88	0.7	1100
AppProfiler (2013)^43^	0.89	0.8	1000
Andrubis (2014)^26^	0.88	0.8	980
Androguard (2015)^44^	0.88	0.7	1100
CopperDroid (2015)^45^	0.78	0.7	1300
DroidDetector (2016)^6^	0.80	0.7	1000
MAMADROID (2016)^11^	0.82	0.6	800
DroidSieve (2017)^46^	0.88	0.7	920
PIndroid (2017)^47^	0.89	0.8	810
MOCDroid (2017)^48^	0.88	0.5	500
DroidDet (2018)^7^	0.88	0.32	430
MalDozer (2018)^49^	0.90	0.3	320
Enmobile (2018)^29^	0.88	0.7	380
SeqDroid (2019)^50^	0.92	0.2	290
MaMaDroid (2019)^97^	0.93	0.2	300
DaDiDroid (2019)^27^	0.91	0.6	330
DeepDroid (2019)^13^	0.91	0.6	330
DL-Droid (2020)^51^	0.93	0.19	200
PerbDroid (2020)^15^	0.91	0.6	330
Proposed approach (i.e., PermDroid)	0.982	0.1	100

Averaged time is calculated by taking training and testing time-period and using Drebin data set.

**Table 15:** Comparison with different approaches/frameworks proposed in the literature.

Framework/approach	Goal	Methodology	Deployment	Data set used while developing	Detection rate	Availability
Paranoid Android^31^ (2010)	Detection	Behavioural and Dynamic	Off-device	Limited	–	–
Crowdroid^34^ (2011)	Detection	Dynamic,	Distributed	Very-Limited	High	–
		System call/API and Behavioural				
Aurasium^25^(2012)	Detection	Dynamic and Behavioural	Off-device	Limited	High	Free
Andromaly^23^ (2012)	Detection	Dynamic and Profile-based	Distributed	Very-Limited	High	Free
AndroSimilar^21^(2013)	Detection	Static	Off-device	Limited	Lesser	–
TaintDroid^30^ (2014)	Detection	Dynamic	Off-Device	Very-Limited	Lesser	Free
		System call/API and Behavioural				
Andrubis^26^ (2014)	Analysis and Detection	Static, Dynamic,	Off-device	Higher	Lesser	Free
		Profile-based and Behavioural				
CopperDroid^45^(2015)	Analysis and Detection	Dynamic, System/API	Off-Device	Limited	Lesser	Free
		and VMI				
HinDroid^98^(2017)	Detection	Dynamic and API	Off-device	Limited	Lesser	–
HEMD^99^(2018)	Detection	Dynamic and Permissions	Off-device	Limited	Lesser	–
MalDozer^49^(2018)	Detection	Dynamic	Off-Device	Limited	Lesser	–
DroidDet^7^(2018)	Detection	Static	Off-device	Limited	Lesser	–
Wei Wang^100^(2019)	Detection	Dynamic	Off-device	Limited	Lesser	–
MalInsight^101^(2019)	Detection	Dynamic	Off-device	Limited	High	–
MLDroid^3^ (2020)	Detection	Dynamic	On-device	Unlimited	High	Free
GDroid^2^ (2021)	Detection	Static	Off-device	Limited	Lesser	Free
IntDroid^102^ (2021)	Detection	Static	Off-device	Limited	Lesser	–
DNNDroid^103^ (2022)	Detection	Dynamic	Off-device	Limited	Moderate	Free
PARUDroid^104^(2023)	Detection	Dynamic	On-device	Limited	Moderate	Free
YarowskyDroid^105^ (2023)	Detection	Dynamic	Off-device	Limited	Lesser	Free
PermDroid (our proposed framework)	Detection	Dynamic,Permissions,	Off-device	Unlimited	Higher	Free
		API calls, user-rating				
		and Number of user download app				

Experiment was performed by using Drebin data set (https://www.sec.cs.tu-bs.de/~danarp/drebin/download.html).

**Table 16:** PermDroid and antivirus scanner detection rates.

	Cyren	Ikarus	VIPRE	McAfee	AVG	AVware	ESET NOD32	CAT QuickHeal	AegisLab	NANO Antivirus	SF with DNN	SF with DNN
Full data set	82%	82.68%	89%	89%	90%	92.8%	92.9%	96.9%	97.1%	96.2%	98.8%	98.8%
Speed in detecting	60	62	40	30	32	30	20	32	30	20	12	12
malware in Sec												

For this experiment, we use .apk file that’s less than 27 MB in size. The experiment was carried out using 1000 different Android apps from the real world.

When compared to the various anti-viruses employed in the experiment, PermDroid performs significantly better. The results of the anti-virus scanner study are shown in Table 16. The anti-virus scanners’ rates of virus detection vary widely. While the most effective scanners catch 97.1 percent of malware, some scanners only catch 82 percent of hazardous samples, which is probably a result of their inexperience with Android malware. PermDroid with DNN and NDTF outperform 1 out of 10 anti-virus scanners on the complete data set, with detection rates of 98.8% and 98.8%, respectively. Out of implemented different anti-virus scanners, it is discovered that at least two of them are capable of identifying every malware sample used in this study. As a result, it may conclude that PermDroid is more effective than many anti-virus scanners’ manually built signatures.

#### Identification of both well-known and new malware families

**Figure 20:**
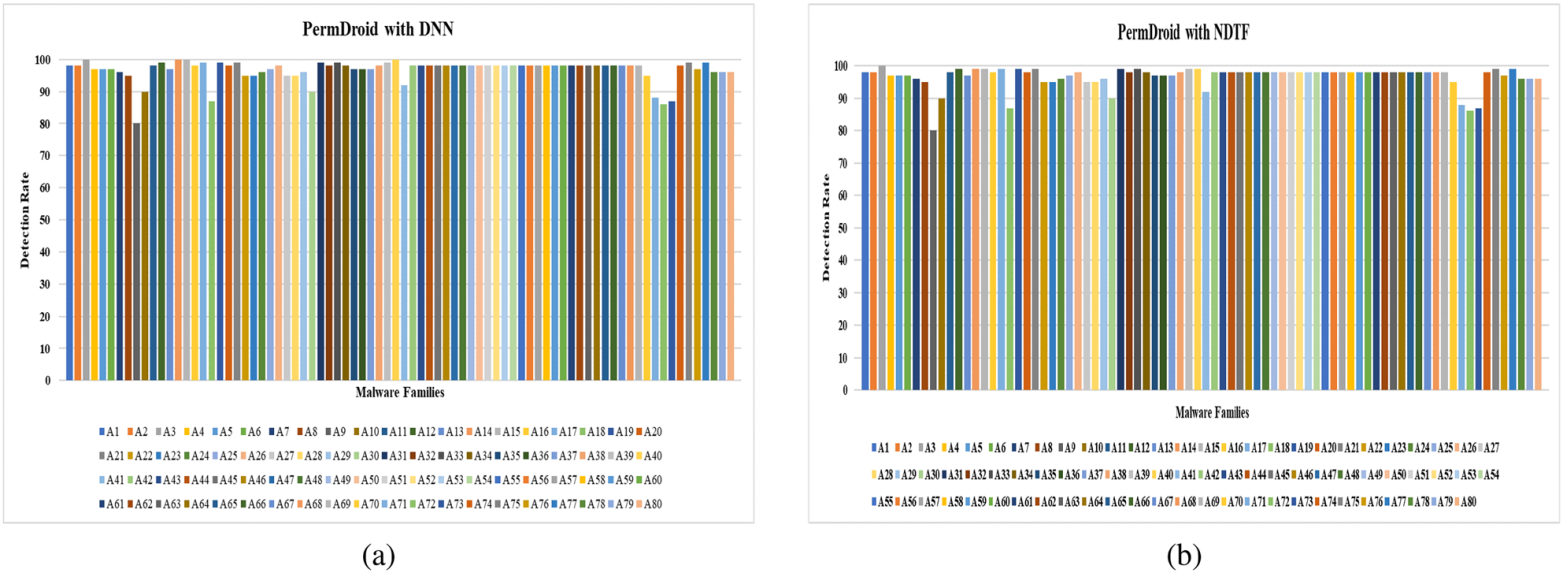
Detection rate of PermDroid with DNN and NDTF.

*Detection of well-known malware families* An experiment is also performed to identify whether or not our suggested framework, i.e., PermDroid, is capable of detecting malware from well-known families. The experiment is carried out on a sample of 20 families from each family (in our research paper, we collect 141 different malware families). According to empirical results, the suggested framework with DNN is capable of detecting an average of 98.8% of malware-infected apps, and the proposed framework with NDTF is likewise capable of doing the same. Table 17 lists the family names and the number of samples for each family, and Fig. 20a,b show PermDroid’s detection performance for each family (Detection rates for some families are lower because of fewer samples in the data set).

**Table 17:** Top malware families are taken into account in our data set.

ID	Family	# of samples	ID	Family	# of samples	ID	Family	# of samples
A1	Airpush	500	A2	AndroRAT	140	A3	Andup	300
A4	Aples	120	A5	BankBot	100	A6	Bankun	133
A7	Boqx	130	A8	Boxer	122	A9	Cova	100
A10	Dowgin	100	A11	DroidKungFu	100	A12	Erop	120
A13	FakeAngry	110	A14	FakeAV	120	A15	FakeDoc	120
A16	FakeInst	110	A17	FakePlayer	120	A18	FakeTimer	120
A19	FakeUpdates	120	A20	Finspy	1110	A21	Fjcon	1230
A22	Fobus	1020	A23	Fusob	1810	A24	GingerMaster	1920
A25	GoldDream	200	A26	Gorpo	120	A27	Gumen	200
A28	Jisut	620	A29	Kemoge	720	A30	Koler	200
A31	Ksapp	290	A32	Kuguo	100	A33	Kyview	500
A34	Leech	30	A35	Lnk	100	A36	Lotoor	20
A37	Mecor	29	A38	Minimob	33	A39	Mmarketpay	200
A40	MobileTX	50	A41	Mseg	23	A42	Mtk	20
A43	Nandrobox	10	A44	Obad	100	A45	Opfake	120
A46	Penetho	120	A47	Ramnit	120	A48	Roop	120
A49	RuMMS	100	A50	SimpleLocker	110	A51	SlemBunk	120
A52	SmsKey	120	A53	SMsZombie	110	A54	Spambot	115
A55	SpyBubble	120	A56	Stealer	300	A57	Steek	230
A58	Svpeng	20	A59	Tesbo	21	A60	Triada	200
A61	Univert	210	A62	UpdtKiller	100	A63	Utchi	300
A64	Vidro	92	A65	VikingHorde	230	A66	Vmvol	533
A67	Winge	190	A68	Youmi	689	A69	Zitmo	230
A70	Ztorg	1000	A71	Imlog	50	A72	SMSreg	50
A73	Gappusin	50	A74	Adrd	50	A75	Geinimi	100
A76	Kmin	157	A77	Plankton	125	A78	GingerMaster	100
A79	Iconosys	100	A80	SendPay	18	A81	GoldDream	200

*Detection of new malware families* To examine if the suggested framework, is capable of identifying unknown malware families, PermDroid is trained with a random sample of 10 distinct families based on counting and then test is performed on the remaining families. Table 18 shows the outcomes in which PermDroid is trained with limited malware samples, which is required to generalize the characteristics of most malware families, and achieved a higher detection rate.

**Table 18:** Detecting unknown malware families with the help of the PermDroid framework proposed in this study.

Combination of Android malware families to trained the model	Detection rate when trained PermDroid with DNN	Detection rat when trained PermDroi with NDTF
{**A1**, up to..... , A10}	66%	71%
{**A1**, A3, up to..... , A11}	70%	69%
:	:	:
:	:	:
:	:	:
{**A2**, up to ..... , A11}	59%	55%
:	:	:
:	:	:
:	:	:
:	:	:
:	:	:
:	:	:
{**A7**, up to ..... , A51}	98.4%	98%
:	:	:
:	:	:
:	:	:
:	:	:
:	:	:
:	:	:

### Experimental outcomes

The conclusions reached after conducting experimental work are presented in this section of the paper. The empirical work was done using a neural network and six different machine learning techniques, including GDA, NM, GD, GDM, LM, and DNN, as well as three ensemble approaches. The developed models outperform previously used classifiers in the literature (Table 11) and can detect malware from both known and unknown families (Table 18, Fig. 20). Additionally, they increase the rate of detection by different Antivirus scanners (stated in Table 15). It is clear from Fig. 20 and Tables 14, 15, 16, and 18 that:

- PermDroid can detect 98.8% of Android malware, which is impossible for most AV scanners on the market.
- With a detection rate of 98.8% for both known and unknown malware types, PermDroid is capable of finding malware.

The proposed framework is able to answer the research questions mentioned in “Research questions” section:

1. To verify the importance of the correlation between the feature sets and the malware detection model, the *t*-test and ULR analysis are used. It is discovered that there are several separate sets of features that are highly connected with the creation of malware detection models as a result of this research.
2. From Fig. 11, it can be noticed that certain sets of features pass a high correlation with other sets of features (i.e., the case with a black square is having high negative correlation, and the case with a black circle is having a high positive correlation). It is essential to remove the collinearity among the features, for calculating the ability of each feature. In this manner, the models developed by selecting sets of the feature are capable to detect malware and do not suffer from the aspect of collinearity.
3. Forward stepwise selection process, ULR, correlation analysis, and *t*-test analysis are implemented to select features that are able to identify whether the app is malicious or not. The model built by applying the specified sets of features produces better outcomes when compared to the rest, according to *t*-test analysis.
4. Six various types of machine learning techniques based on neural network principles, such as NM, GD, LM, GDM, GDA, and DNN, as well as three ensemble approaches, are implemented in detecting whether an app is benign or malicious. From the Tables 8 and 9, it is apparent that the model developed using an ANN and the Deep Neural Network (DNN) approach produces the best results when compared to other techniques.
5. Tables 8 and 9 and Figs. 18, 19 and 20 show that our suggested model is effective in identifying malware from real-world apps when API calls, permissions, app rating, and the number of people that have downloaded the app are all considered features.

## Threats to validity

In this section, threats to validity are discussed that are experienced while performing the experiment. Three different threats are mentioned below:

i. *Construct validity* The Android malware detection methodology in this research study is capable of detecting whether an app is benign or malicious, however it does not specify how many features are needed to find vulnerabilities in Android apps.
ii. *Internal validity* The homogeneity of the data set employed in this research work is the second threat. Apps are collected from a variety of promised repositories. Any errors made while gathering data from these sources are not taken into account in this study. Although, it cannot promise that the data collected and retrieved for our analysis is 100 percent accurate, it can be believed that it assembled consistently.
iii. * External validity* To train the Android malware detection algorithm, 141 different malware families are considered. Furthermore, the research can be extended to include other malware families in order to train the technique to identify malicious apps.

## Conclusion and future work

This study suggests a framework for selecting small set of features that helps in detecting malware from Android apps. The following are our observations based on the basis of our proposed framework in this research paper:

- Based on the feature selection method, it is discovered that there is a limited group of attributes that can detect malware or benign apps with greater accuracy and lower values of incorrectly classified errors.
- Using our feature selection method sets S25, S28, S19, S14, S9, and S4 of features were discovered to be important malware detectors.
- Based on the Wilcoxon signed-rank test, it is found that there is a significant difference between all extracted features and the selected feature sets. It is found that, after calculating the mean difference that the model developed with the input of the selected feature sets outperformed the model with the input of all extracted feature sets.
- Different classification algorithms differ significantly, according to the Wilcoxon signed-rank test. By calculating the mean difference value, it is discovered that the model created by combining a neural network with the Deep-Learning machine-learning algorithm produced superior results than the other machine learning methods used in this study.
- It may be inferred from the results of the experiments that the NDTF approach performed better than other ensemble methods.
- Our used classifier outperformed the performance of the classifiers used in the literature, as shown in Fig. 20 and Tables 11 and 14.
- According to the results of the experiments (Tables 8, 9), the malware detection model built was not significantly harmed after deleting 60% of the possible number of sets of features; in fact, in almost all cases, the results were better.
- As shown in Table 18 and Fig. 20, our proposed malware detection system can detect malware from both known and undiscovered malware families.

This study established that a malware detection method merely identifies whether an app is malicious or benign. Several avenues can be explored for future research. Firstly, a large amount of Android apps are required to develop the model, memorize and disclose information related to the data set. Second, it is also difficult to make a centralized system at the time of training and testing the model. Third, decentralized, privacy-preserving classifier model will be proposed for detecting Android malwares. Further, it is also be discovered how many permissions are necessary to evaluate whether an app is dangerous or not, more investigation may be done.

## Supplementary Information

Supplementary Information.

## Data Availability

For materials should be addressed to corresponding authors.

## References

[1] Faruki, P. *et al.* Android security: A survey of issues, malware penetration, and defenses. *IEEE Commun. Surv. Tutor.* **17**(2), 998–1022 (2014).

[2] Gao, H., Cheng, S. & Zhang, W. Gdroid: Android malware detection and classification with graph convolutional network. *Comput. Secur.* **106**, 102264 (2021).

[3] Mahindru, A. & Sangal, A. MLDroid—framework for android malware detection using machine learning techniques. *Neural Comput. Appl.* **33**, 1–58 (2020).

[4] Fereidooni, H., Conti, M., Yao, D. & Sperduti, A. Anastasia: Android malware detection using static analysis of applications. In *2016 8th IFIP International Conference on New Technologies, Mobility and Security (NTMS)*, 1–5 (IEEE, 2016).

[5] Arp, D. *et al.* Drebin: Effective and explainable detection of android malware in your pocket. *Ndss* **14**, 23–26 (2014).

[6] Yuan, Z., Lu, Y. & Xue, Y. Droiddetector: Android malware characterization and detection using deep learning. *Tsinghua Sci. Technol.* **21**(1), 114–123 (2016).

[7] Zhu, H. J. *et al.* Droiddet: Effective and robust detection of android malware using static analysis along with rotation forest model. *Neurocomputing* **272**, 638–646 (2018).

[8] Wong, M. Y. & Lie, D. Intellidroid: A targeted input generator for the dynamic analysis of android malware. *NDSS* **16**, 21–24 (2016).

[9] Dash, S. K., Suarez-Tangil, G., Khan, S., Tam, K., Ahmadi, M., Kinder, J. & Cavallaro, L. Droidscribe: Classifying android malware based on runtime behavior. In: *2016 IEEE Security and Privacy Workshops (SPW)*, 252–261 (IEEE, 2016).

[10] Chen, S., Xue, M., Tang, Z., Xu, L. & Zhu, H. Stormdroid: A streaminglized machine learning-based system for detecting android malware. In *Proceedings of the 11th ACM on Asia Conference on Computer and Communications Security*, 377–388 (2016).

[11] Mariconti, E., Onwuzurike, L., Andriotis, P., Cristofaro, E. D., Ross, G. & Stringhini, G. *Mamadroid: Detecting Android Malware by Building Markov Chains of Behavioral Models*. arXiv:1612.04433 (2016)

[12] Kabakus, A. T. DroidMalwareDetector: A novel android malware detection framework based on convolutional neural network. *Expert Syst. Appl.* **206**, 117833 (2022).

[13] Mahindru, A. & Sangal, A. Deepdroid: Feature selection approach to detect android malware using deep learning. In: *2019 IEEE 10th International Conference on Software Engineering and Service Science (ICSESS)*, 16–19 (IEEE, 2019).

[14] Mahindru, A. & Sangal, A. Feature-based semi-supervised learning to detect malware from android. In *Automated Software Engineering: A Deep Learning-Based Approach*, 93–118 (Springer, 2020).

[15] Mahindru, A. & Sangal, A. Perbdroid: Effective malware detection model developed using machine learning classification techniques. In *A Journey Towards Bio-inspired Techniques in Software Engineering* 103–139 (Springer, 2020).

[16] Mahindru, A. & Sangal, A. Hybridroid: An empirical analysis on effective malware detection model developed using ensemble methods. *J. Supercomput.* **77**(8), 8209–8251 (2021).

[17] Mahindru, A. & Sangal, A. Semidroid: A behavioral malware detector based on unsupervised machine learning techniques using feature selection approaches. *Int. J. Mach. Learn. Cybern.* **12**(5), 1369–1411 (2021).

[18] Zhao, Y. *et al.* On the impact of sample duplication in machine-learning-based android malware detection. *ACM Trans. Softw. Eng. Methodol. (TOSEM)* **30**(3), 1–38 (2021).

[19] Yumlembam, R., Issac, B., Jacob, S. M. & Yang L. IoT-based android malware detection using graph neural network with adversarial defense. *IEEE Internet Things J.* (2022).

[20] Kumar, L., Misra, S. & Rath, S. K. An empirical analysis of the effectiveness of software metrics and fault prediction model for identifying faulty classes. *Comput. Stand. Interfaces* **53**, 1–32 (2017).

[21] Faruki, P., Ganmoor, V., Laxmi, V., Gaur, M. S. & Bharmal, A. Androsimilar: Robust statistical feature signature for android malware detection. In *Proceedings of the 6th International Conference on Security of Information and Networks*, 152–159 (2013).

[22] Milosevic, J., Malek, M. & Ferrante, A. Time, accuracy and power consumption tradeoff in mobile malware detection systems. *Comput. Secur.* **82**, 314–328 (2019).

[23] Shabtai, A., Kanonov, U., Elovici, Y., Glezer, C. & Weiss, Y. Andromaly: A behavioral malware detection framework for android devices. *J. Intell. Inf. Syst.* **38**(1), 161–190 (2012).

[24] Badhani, S. & Muttoo, S. K. Android malware detection using code graphs. In *System Performance and Management Analytics*, 203–215 (Springer, 2019).

[25] Xu, R., Saïdi, H. & Anderson, R. Aurasium: Practical policy enforcement for android applications. In *Presented as part of the 21st*USENIX*Security Symposium* (USENIX*Security 12*), 539–552 (2012).

[26] Lindorfer, M., Neugschwandtner, M., Weichselbaum, L., Fratantonio, Y., Veen, V. V. D. & Platzer, C. (2014) Andrubis–1,000,000 apps later: A view on current android malware behaviors. In *2014 Third International Workshop on Building Analysis Datasets and Gathering Experience Returns for Security (BADGERS)*, 3–17 (IEEE).

[27] Ikram, M., Beaume, P. & Kâafar, M. A. *Dadidroid: An Obfuscation Resilient Tool for Detecting Android Malware via Weighted Directed Call Graph Modelling*. arXiv:1905.09136 (2019).

[28] Shen, F., Vecchio, J. D., Mohaisen, A., Ko, S. Y. & Ziarek, L. Android malware detection using complex-flows. *IEEE Trans. Mob. Comput.* **18**(6), 1231–1245 (2018).

[29] Yang, W., Prasad, M. R. & Xie, T. Enmobile: Entity-based characterization and analysis of mobile malware. In *Proceedings of the 40th International Conference on Software Engineering*, 384–394 (2018).

[30] Enck, W. *et al.* Taintdroid: an information-flow tracking system for realtime privacy monitoring on smartphones. *ACM Trans. Comput. Syst. (TOCS)* **32**(2), 1–29 (2014).

[31] Portokalidis, G., Homburg, P., Anagnostakis, K. & Bos, H. (2010) Paranoid android: Versatile protection for smartphones. In *Proceedings of the 26th Annual Computer Security Applications Conference*, 347–356.

[32] Bläsing, T., Batyuk, L., Schmidt, A. D., Camtepe, S. A. & Albayrak, S. An android application sandbox system for suspicious software detection. In *2010 5th International Conference on Malicious and Unwanted Software*, 55–62 (IEEE, 2010).

[33] Aubery-Derrick, S. *Detection of Smart Phone Malware*. Unpublished Ph.D. Thesis, 1–211 (Electronic and Information Technology University, Berlin, 2011).

[34] Burguera, I., Zurutuza, U. & Nadjm-Tehrani, S. Crowdroid: Behavior-based malware detection system for android. In *Proceedings of the 1st ACM Workshop on Security and Privacy in Smartphones and Mobile Devices*, 15–26 (2011).

[35] Grace, M. C., Zhou, Y., Wang, Z. & Jiang, X. Systematic detection of capability leaks in stock android smartphones. In *NDSS*, vol 14, 19 (2012).

[36] Grace, M., Zhou, Y., Zhang, Q., Zou, S. & Jiang, X. Riskranker: Scalable and accurate zero-day android malware detection. In *Proceedings of the 10th International Conference on Mobile Systems, Applications, and Services*, 281–294 (2012).

[37] Zheng, C., Zhu, S., Dai, S., Gu, G., Gong, X., Han, X. & Zou, W. Smartdroid: An automatic system for revealing UI-based trigger conditions in android applications. In *Proceedings of the Second ACM Workshop on Security and Privacy in Smartphones and Mobile Devices*, 93–104 (2012).

[38] Dini, G., Martinelli, F., Saracino, A. & Sgandurra, D. Madam: A multi-level anomaly detector for android malware. In *International Conference on Mathematical Methods, Models, and Architectures for Computer Network Security*, 240–253 (Springer, 2012).

[39] Yan, L. K. & Yin, H. Droidscope: Seamlessly reconstructing the OS and Dalvik semantic views for dynamic android malware analysis. In *Presented as part of the 21st*USENIX*Security Symposium* (USENIX*Security 12*), 569–584 (2012).

[40] Backes, M., Gerling, S., Hammer, C., Maffei, M. & von Styp-Rekowsky, P. Appguard–enforcing user requirements on android apps. In *International Conference on TOOLS and Algorithms for the Construction and Analysis of Systems*, 543–548 (Springer, 2013).

[41] Shahzad, F., Akbar, M., Khan, S. & Farooq, M. *Tstructdroid: Realtime malware detection using in-execution dynamic analysis of kernel process control blocks on android*. Tech Rep (National University of Computer and Emerging Sciences, Islamabad, 2013).

[42] Rastogi, V., Chen, Y. & Enck, W. Appsplayground: Automatic security analysis of smartphone applications. In *Proceedings of the third ACM Conference on Data and Application Security and Privacy*, 209–220 (2013).

[43] Rosen, S., Qian, Z. & Mao, Z. M. Appprofiler: A flexible method of exposing privacy-related behavior in android applications to end users. In *Proceedings of the Third ACM Conference on Data and Application Security and Privacy*, 221–232 (2013).

[44] Desnos, A. *et al*. Androguard-reverse engineering, malware and goodware analysis of android applications. URL code google com/p/androguard 153 (2013).

[45] Tam, K., Khan, S. J., Fattori, A. & Cavallaro, L. Copperdroid: Automatic reconstruction of android malware behaviors. In *Ndss* (2015).

[46] Suarez-Tangil, G., Dash, S. K., Ahmadi, M., Kinder, J., Giacinto, G. & Cavallaro, L. Droidsieve: Fast and accurate classification of obfuscated android malware. In *Proceedings of the Seventh ACM on Conference on Data and Application Security and Privacy*, 309–320 (2017).

[47] Idrees, F., Rajarajan, M., Conti, M., Chen, T. M. & Rahulamathavan, Y. Pindroid: A novel android malware detection system using ensemble learning methods. *Comput. Secur.* **68**, 36–46 (2017).

[48] Martín, A., Menéndez, H. D. & Camacho, D. Mocdroid: Multi-objective evolutionary classifier for android malware detection. *Soft. Comput.* **21**(24), 7405–7415 (2017).

[49] Karbab, E. B., Debbabi, M., Derhab, A. & Mouheb, D. Maldozer: Automatic framework for android malware detection using deep learning. *Digit. Investig.* **24**, S48–S59 (2018).

[50] Lee, W. Y., Saxe, J. & Harang, R. Seqdroid: Obfuscated android malware detection using stacked convolutional and recurrent neural networks. In *Deep Learning Applications for Cyber Security*, 197–210 (Springer, 2019).

[51] Alzaylaee, M. K., Yerima, S. Y. & Sezer, S. DL-Droid: Deep learning based android malware detection using real devices. *Comput. Secur.* **89**, 101663 (2020).

[52] Yuan, Z., Lu, Y., Wang, Z. & Xue, Y. Droid-sec: Deep learning in android malware detection. In *Proceedings of the 2014 ACM Conference on SIGCOMM*, 371–372 (2014).

[53] Zhang, M., Duan, Y., Yin, H. & Zhao, Z. Semantics-aware android malware classification using weighted contextual API dependency graphs. In *Proceedings of the 2014 ACM SIGSAC Conference on Computer and Communications Security*, 1105–1116 (2014).

[54] Shankar, V. G., Somani, G., Gaur, M. S., Laxmi, V. & Conti, M. Androtaint: An efficient android malware detection framework using dynamic taint analysis. In *2017 ISEA Asia Security and Privacy (ISEASP)*, 1–13 (IEEE, 2017).

[55] Mahindru, A. & Singh, P. Dynamic permissions based android malware detection using machine learning techniques. In *Proceedings of the 10th Innovations in Software Engineering Conference*, 202–210 (2017).

[56] Shi, B. *et al.* Prediction of recurrent spontaneous abortion using evolutionary machine learning with joint self-adaptive sime mould algorithm. *Comput. Biol. Med.* **148**, 105885 (2022). 35930957 10.1016/j.compbiomed.2022.105885

[57] Zhang, Q., Wang, D. & Wang, Y. Convergence of decomposition methods for support vector machines. *Neurocomputing* **317**, 179–187 (2018).

[58] Hou, S., Saas, A., Chen, L. & Ye, Y. Deep4maldroid: A deep learning framework for android malware detection based on linux kernel system call graphs. In *2016 IEEE/WIC/ACM International Conference on Web Intelligence Workshops (WIW)*, 104–111 (IEEE, 2016).

[59] Nix, R. & Zhang, J. Classification of android apps and malware using deep neural networks. In *2017 International Joint Conference on Neural Networks (IJCNN)*, 1871–1878 (IEEE, 2017).

[60] Zhang, X. A deep learning based framework for detecting and visualizing online malicious advertisement. Ph.D. Thesis, University of New Brunswick (2018)

[61] Nauman, M., Tanveer, T. A., Khan, S. & Syed, T. A. Deep neural architectures for large scale android malware analysis. *Clust. Comput.* **21**(1), 569–588 (2018).

[62] Xiao, X., Wang, Z., Li, Q., Xia, S. & Jiang, Y. Back-propagation neural network on Markov chains from system call sequences: a new approach for detecting android malware with system call sequences. *IET Inf. Secur.* **11**(1), 8–15 (2016).

[63] Martinelli, F., Marulli, F. & Mercaldo, F. Evaluating convolutional neural network for effective mobile malware detection. *Procedia Comput. Sci.* **112**, 2372–2381 (2017).

[64] Xiao, X., Zhang, S., Mercaldo, F., Hu, G. & Sangaiah, A. K. Android malware detection based on system call sequences and LSTM. *Multim. Tools Appl.* **78**(4), 3979–3999 (2019).

[65] Dimjašević, M., Atzeni, S., Ugrina, I. & Rakamaric, Z. Evaluation of android malware detection based on system calls. In *Proceedings of the 2016 ACM on International Workshop on Security and Privacy Analytics*, 1–8 (2016).

[66] Mas’ud, M. Z., Sahib, S., Abdollah, M. F., Selamat, S. R. & Yusof, R. Analysis of features selection and machine learning classifier in android malware detection. In *2014 International Conference on Information Science and Applications (ICISA)*, 1–5 (IEEE, 2014).

[67] Yerima, S. Y., Sezer, S., McWilliams, G. & Muttik, I. A new android malware detection approach using Bayesian classification. In *2013 IEEE 27th International Conference on Advanced Information Networking and Applications (AINA)*, 121–128 (IEEE, 2013).

[68] Narudin, F. A., Feizollah, A., Anuar, N. B. & Gani, A. Evaluation of machine learning classifiers for mobile malware detection. *Soft. Comput.* **20**(1), 343–357 (2016).

[69] Wang, W. *et al.* Exploring permission-induced risk in android applications for malicious application detection. *IEEE Trans. Inf. Forensics Secur.* **9**(11), 1869–1882 (2014).

[70] Ayar, M., Isazadeh, A., Gharehchopogh, F. S. & Seyedi, M. NSICA: Multi-objective imperialist competitive algorithm for feature selection in arrhythmia diagnosis. *Comput. Biol. Med.* **161**, 107025 (2023). 37245373 10.1016/j.compbiomed.2023.107025

[71] Hu, H. *et al.* Dynamic individual selection and crossover boosted forensic-based investigation algorithm for global optimization and feature selection. *J. Bionic Eng.* **20**, 1–27 (2023).

[72] Zhong, C., Li, G., Meng, Z., Li, H. & He, W. A self-adaptive quantum equilibrium optimizer with artificial bee colony for feature selection. *Comput. Biol. Med.* **153**, 106520 (2023). 36608463 10.1016/j.compbiomed.2022.106520

[73] Zhou, P. *et al.* Unsupervised feature selection for balanced clustering. *Knowl.-Based Syst.* **193**, 105417 (2020).

[74] Allix, K. *et al.* Empirical assessment of machine learning-based malware detectors for android. *Empir. Softw. Eng.* **21**(1), 183–211 (2016).

[75] Narayanan, A., Chandramohan, M., Chen, L. & Liu, Y. A multi-view context-aware approach to android malware detection and malicious code localization. *Empir. Softw. Eng.* **23**(3), 1222–1274 (2018).

[76] Azmoodeh, A., Dehghantanha, A. & Choo, K. K. R. Robust malware detection for internet of (battlefield) things devices using deep eigenspace learning. *IEEE Trans. Sustain. Comput.* **4**(1), 88–95 (2018).

[77] Chen, K. Z., Johnson, N. M., D’Silva, V., Dai, S., MacNamara, K., Magrino, T. R., Wu, E. X., Rinard, M. & Song, D. X. Contextual policy enforcement in android applications with permission event graphs. In: *NDSS*, 234 (2013).

[78] Yerima, S. Y., Sezer, S. & McWilliams, G. Analysis of Bayesian classification-based approaches for android malware detection. *IET Inf. Secur.* **8**(1), 25–36 (2014).

[79] Gonzalez, H., Stakhanova, N. & Ghorbani, A. A. Droidkin: Lightweight detection of android apps similarity. In *International Conference on Security and Privacy in Communication Networks*, 436–453 (Springer, 2014) .

[80] Kadir, A. F. A., Stakhanova, N. & Ghorbani, A. A. Android botnets: What urls are telling us. In *International Conference on Network and System Security*, 78–91 (Springer, 2015).

[81] Zhou, Y. & Jiang, X. Android malware genome project. Disponibile a http://www.malgenomeproject.org (2012).

[82] Garcia, J., Hammad, M. & Malek, S. Lightweight, obfuscation-resilient detection and family identification of android malware. *ACM Trans. Softw. Eng. Methodol. (TOSEM)* **26**(3), 1–29 (2018).

[83] Mahindru, A. & Sangal, A. Parudroid: Validation of android malware detection dataset. *J. Cybersecur. Inform. Manag.* **3**(2), 42–52 (2020).

[84] McCulloch, W. S. & Pitts, W. A logical calculus of the ideas immanent in nervous activity. *Bull. Math. Biophys.* **5**(4), 115–133 (1943). 2185863

[85] Faruk, M. J. H., Shahriar, H., Valero, M., Barsha, F. L., Sobhan, S., Khan, M. A., Whitman, M., Cuzzocrea, A., Lo, D., Rahman, A., *et al*. Malware detection and prevention using artificial intelligence techniques. In *2021 IEEE International Conference on Big Data (Big Data)*, 5369–5377 (IEEE, 2021).

[86] Battiti, R. First-and second-order methods for learning: Between steepest descent and newton’s method. *Neural Comput.* **4**(2), 141–166 (1992).

[87] Levenberg, K. A method for the solution of certain non-linear problems in least squares. *Q. Appl. Math.* **2**(2), 164–168 (1944).

[88] Bengio, Y. Learning deep architectures for AI. *Found. Trends*® *Mach. Learn.* **2**(1), 1–127 (2009).

[89] Kaur, J., Singh, S., Kahlon, K. S. & Bassi, P. Neural network-a novel technique for software effort estimation. *Int. J. Comput. Theory Eng.* **2**(1), 17 (2010).

[90] Doraisamy, S., Golzari, S., Mohd, N., Sulaiman, M. N. & Udzir, N. I. A study on feature selection and classification techniques for automatic genre classification of traditional Malay music. In *ISMIR*, 331–336 (2008).

[91] Forman, G. An extensive empirical study of feature selection metrics for text classification. *J. Mach. Learn. Res.* **3**(Mar), 1289–1305 (2003).

[92] Furlanello, C., Serafini, M., Merler, S. & Jurman, G. Entropy-based gene ranking without selection bias for the predictive classification of microarray data. *BMC Bioinform.* **4**(1), 54 (2003). 10.1186/1471-2105-4-54 PMC293475 14604446

[93] Coronado-De-Alba, L. D., Rodríguez-Mota, A. & Escamilla-Ambrosio, P. J. Feature selection and ensemble of classifiers for android malware detection. In *2016 8th IEEE Latin-American Conference on Communications (LATINCOM)*, 1–6 (IEEE, 2016).

[94] Deepa, K., Radhamani, G. & Vinod, P. Investigation of feature selection methods for android malware analysis. *Procedia Comput. Sci.* **46**, 841–848 (2015).

[95] Kothari, C. R. Research methodology: Methods and techniques. New Age International (2004).

[96] Chaikla, N. & Qi, Y. Genetic algorithms in feature selection. In *IEEE SMC’99 Conference Proceedings. 1999 IEEE International Conference on Systems, Man, and Cybernetics (Cat. No. 99CH37028)*, vol 5, 538–540 (IEEE, 1999).

[97] Onwuzurike, L. *et al.* Mamadroid: Detecting android malware by building Markov chains of behavioral models (extended version). *ACM Trans. Privacy Secur. (TOPS)* **22**(2), 1–34 (2019).

[98] Hou, S., Ye, Y., Song, Y. & Abdulhayoglu, M. Hindroid: An intelligent android malware detection system based on structured heterogeneous information network. In *Proceedings of the 23rd ACM SIGKDD International Conference on Knowledge Discovery and Data Mining*, 1507–1515 (2017) .

[99] Zhu, H. J. *et al.* HEMD: A highly efficient random forest-based malware detection framework for android. *Neural Comput. Appl.* **30**(11), 3353–3361 (2018).

[100] Wang, W., Zhao, M. & Wang, J. Effective android malware detection with a hybrid model based on deep autoencoder and convolutional neural network. *J. Ambient. Intell. Humaniz. Comput.* **10**(8), 3035–3043 (2019).

[101] Han, W., Xue, J., Wang, Y., Liu, Z. & Kong, Z. Malinsight: A systematic profiling based malware detection framework. *J. Netw. Comput. Appl.* **125**, 236–250 (2019).

[102] Zou, D. *et al.* Intdroid: Android malware detection based on API intimacy analysis. *ACM Trans. Softw. Eng. Methodol. (TOSEM)* **30**(3), 1–32 (2021).

[103] Mahindru, A. & Arora, H. Dnndroid: Android malware detection framework based on federated learning and edge computing. In *International Conference on Advancements in Smart Computing and Information Security*, 96–107 (Springer, 2022).

[104] Mahindru, A. & Arora, H. Parudroid: Framework that enhances smartphone security using an ensemble learning approach. *SN Comput. Sci.* **4**(5), 630 (2023).

[105] Mahindru, A., Sharma, S. K. & Mittal, M. Yarowskydroid: Semi-supervised based android malware detection using federation learning. In *2023 International Conference on Advancement in Computation & Computer Technologies (InCACCT)*, 380–385 (IEEE, 2023).

