# Supplementary material for: PermDroid a framework developed using proposed feature selection approach and machine learning techniques for Android malware detection
Source: Sci Rep. 2024 May 10;14:10724. doi: 10.1038/s41598-024-60982-y (PMC11636933; doi:10.1038/s41598-024-60982-y)
Supplement: Supplementary file 1 — Supplementary Information. [file 41598_2024_60982_MOESM1_ESM.pdf]

Table S1: Accuracy measured by using t-test.

**ID**	GD		GDM		GDA		NM		LM		DNN		BTE		MVE		NDTF	
	**EF**	**SF**	**EF**	**SF**	**EF**	**SF**	**EF**	**SF**	**EF**	**SF**	**EF**	**SF**	**EF**	**SF**	**EF**	**SF**	**EF**	**SF**
D1	52.4	54.5	46	51.6	44.6	52.4	54	55	52.4	**63.1**	55	59.5	50.5	55	49.2	59.2	54.5	59.6
D2	53	59.4	49.51	54.430	45	53	43	49.61	52	59.6	59	**63.3**	51	56	54	61	53	59
D3	54	59	46	49.92	51	56	50	55	52	56	59	**64.9**	46	50	52	56	56	60
D4	53	55	46	49	52	55	51	56	50	53	56	59.1	46	50	51	53	54	**60**
D5	50	53	42	46.9	52	56	45	52	50.3	56.5	54	59.1	46	52	53	54	55	**61**
D6	50	53	51	59.9	46	52	45	54	50	54	52	**61.1**	46	49.9	51	54	53	55
D7	44	49	53	**59.9**	51	56	40	45	51	59	50	55	42	46	50	53	52	55
D8	44	53	46	49.9	52	59	54	59	53	59	55	**61.1**	42	45	52	55	56	60.1
D9	44	52	44	49.9	50	54	51	54	53	55	55	65	51	56	53	60	59	**66**
D10	44	52	42	49.9	54	59	51	59	42	56	54	**64.4**	46	55	51	59	55	64
D11	46	59	46	49.9	51	52	40	55	52	62	49	54.1	41	52	54	62	56	**66**
D12	45	59	44	52.3	41	55	40	54	42	56	49	55.1	42	50	52	56	46	**60.0**
D13	43	59	56	69.2	41	56	46	55	42	56	49	**64.1**	45	53	50	56	51	62
D14	54	52	46	59.95	44	52	55	60	54	61	45	**64.1**	42	50	54	60	51	62.0
D15	51	56	56	59.91	51	61	40	55	52	56	51	64.31	56	60	54	56	55	**65**
D16	44	59	46	49.9	51	56	40	57.5	55.1	**66.4**	51	62.1	46	50	52	56	51	62
D17	44	58	46	52.9	50	61	50	55	42	56	49	**63**	46	54	42	61	46	62
D18	44	52	46	52.4	44	56	55	61	52	60	59	64	56	60	52	61	56	**65**
D19	41	59	56	59.94	51	59	46	55	50	56	52	62.1	41	52	50	55	52	**66**
D20	44	55	54	61.6	51	62	40	52	50	59	45	**62.3**	54	60	50	59	52	61
D21	54	61	42	59.9	50	55	45	52	50	59	51	**61.2**	56	59	41	56	46	55
D22	44	52	44	59	51	56	42	50	51	55	51	62	56	62	52	61	56	**63.9**
D23	44	55	46	59	51	58	45	59	42	56	49	60	40	55	42	56	46	**63**
D24	54	58	46	59.9	51	56	50	59	45	56	59	**62.3**	46	55	52	62	56	62
D25	45	59	46	55	55	61	49	52	50	59	52	**62**	51	62.8	52	61	56	61.5
D26	40	52	46	55	50	61	50	59	52	56	59	**64**	56	60	52	61	58	62.5
D27	44	59	46	51.2	50	55	50	55	50	55	52	61.5	50	59	53	61	57.8	**62.6**
D28	54	59	51	59.8	51	60.1	50	61.5	52	60.2	59	**63.7**	56	59	53	61.5	59	63.5
D29	44	52.6	41	52.2	41	56.4	50.4	55.5	52.5	59.6	51	66.1	46	50.5	55.9	60.9	51	**66.4**
D30	51	59	52	59.9	51	58	50	55	52	56	59	**64.1**	53	58	52	56	56	62

Table S2: F-Measure measured by using *t*-test.

**ID**	GD		GDM		GDA		NM		LM		DNN		BTE		MVE		NDTF
	**EF**	**SF**	**EF**	**SF**	**EF**	**SF**	**EF**	**SF**	**EF**	**SF**	**EF**	**SF**	**EF**	**SF**	**EF**	**SF**	**EF**	**SF**
D1	0.49	0.56	0.41	0.59	0.46	0.59	0.50	0.53	0.49	**0.55**	0.45	0.52	0.49	0.52	0.42	0.55	0.49	0.54
D2	0.42	0.56	0.49	0.51	0.49	0.53	0.46	0.55	0.44	**0.60**	0.49	0.552	0.45	0.59	0.45	0.54	0.45	0.594
D3	0.44	0.59	0.44	0.59	0.41	0.52	0.45	0.50	0.45	0.56	0.49	**0.55**	0.46	0.55	0.41	0.59	0.46	0.54
D4	0.42	0.54	0.462	0.59	0.42	0.54	0.41	0.56	0.40	0.54	0.42	0.53	0.44	0.55	0.45	0.53	0.45	**0.59**
D5	0.40	0.52	0.42	0.50	0.42	0.56	0.44	0.52	0.43	0.52	0.41	0.551	0.46	0.52	0.43	0.54	0.45	**0.59**
D6	0.44	0.55	0.44	0.54	0.46	0.52	0.45	0.52	0.41	0.52	0.42	**0.53**	0.46	0.521	0.42	0.524	0.44	0.520
D7	0.44	0.569	0.43	**0.59**	0.46	0.56	0.46	0.54	0.51	0.559	0.50	0.55	0.42	0.56	0.50	0.54	0.52	0.55
D8	0.50	0.54	0.51	0.59	0.52	0.54	0.52	0.54	0.52	0.54	0.56	**0.61**	0.50	0.55	0.521	0.541	0.551	0.59
D9	0.44	0.52	0.45	0.59	0.45	0.52	0.44	0.54	0.43	0.55	0.45	0.55	0.51	0.56	0.45	0.59	0.45	**0.60**
D10	0.44	0.52	0.42	0.53	0.44	0.52	0.45	0.56	0.45	0.53	0.44	**0.52**	0.46	0.51	0.45	0.50	0.45	0.51
D11	0.46	0.53	0.44	0.52	0.45	0.52	0.45	0.52	0.45	0.52	0.49	0.54	0.44	0.52	0.51	0.56	0.53	**0.59**
D12	0.45	0.51	0.44	0.53	0.49	0.52	0.50	0.54	0.52	0.56	0.49	0.53	0.51	0.59	0.522	0.56	0.56	**0.60**
D13	0.53	0.61	0.52	0.59	0.46	0.50	0.46	0.52	0.50	0.56	0.52	**0.64**	0.53	0.62	0.50	0.61	0.51	0.62
D14	0.49	0.52	0.46	0.55	0.54	0.59	0.54	0.59	0.50	0.55	0.55	**0.62**	0.54	0.62	0.54	0.61	0.49	0.55
D15	0.51	0.56	0.46	0.59	0.52	0.56	0.45	0.52	0.52	0.56	0.51	0.61	0.56	0.59	0.45	0.56	0.55	**0.64**
D16	0.44	0.50	0.53	0.59	0.51	0.56	0.49	0.55	0.56	**0.60**	0.51	0.59	0.46	0.51	0.51	0.55	0.50	0.59
D17	0.49	0.53	0.46	0.51	0.51	0.57	0.52	0.58	0.49	0.54	0.59	**0.62**	0.56	0.58	0.56	0.59	0.57	0.60
D18	0.46	0.51	0.46	0.54	0.44	0.51	0.55	0.59	0.54	0.59	0.59	0.62	0.57	0.61	0.52	0.61	0.56	**0.63**
D19	0.49	0.52	0.52	0.61	0.57	0.60	0.52	0.62	0.45	0.52	0.52	0.6	0.49	0.54	0.45	0.51	0.54	**0.61**
D20	0.45	0.54	0.44	0.56	0.57	0.59	0.58	0.62	0.50	0.56	0.51	**0.62**	0.514	0.61	0.45	0.52	0.52	0.59
D21	0.51	0.58	0.41	0.52	0.45	0.51	0.45	0.52	0.50	0.59	0.51	**0.61**	0.46	0.59	0.41	0.50	0.41	0.55
D22	0.50	0.56	0.51	0.59	0.49	0.56	0.54	0.59	0.41	0.51	0.50	0.59	0.46	0.52	0.50	0.56	0.51	**0.61**
D23	0.54	0.59	0.56	0.61	0.51	0.58	0.51	0.59	0.52	0.56	0.49	0.56	0.54	0.59	0.52	0.56	0.56	**0.60**
D24	0.49	0.53	0.46	0.54	0.51	0.56	0.45	0.55	0.45	0.51	0.50	**0.61**	0.41	0.50	0.52	0.59	0.51	0.59
D25	0.51	0.54	0.46	0.51	0.52	0.56	0.50	0.57	0.52	0.59	0.51	**0.61**	0.51	0.56	0.52	0.59	0.46	0.55
D26	0.40	0.52	0.41	0.55	0.40	0.59	0.53	0.58	0.52	0.56	0.59	**0.63**	0.56	0.60	0.52	0.56	0.56	0.62
D27	0.48	0.55	0.56	0.60	0.50	0.55	0.51	0.59	0.45	0.56	0.51	0.59	0.40	0.55	0.41	0.56	0.55	**0.63**
D28	0.44	0.59	0.51	0.61	0.51	0.59	0.45	0.59	0.50	0.59	0.51	**0.60**	0.51	0.61	0.52	0.58	0.52	0.59
D29	0.52	0.58	0.54	0.62	0.41	0.54	0.44	0.51	0.44	0.54	0.41	0.56	0.46	0.55	0.52	0.59	0.51	**0.60**
D30	0.41	0.52	0.46	0.59	0.51	0.56	0.45	0.54	0.51	0.55	0.52	**0.61**	0.49	0.55	0.51	0.55	0.56	0.60

Table S3: Accuracy measured by using ULR analysis.

ID	GD		GDM		GDA		NM		LM		DNN		BTE		MVE		NDTF
	**EF**	**SF**	**EF**	**SF**	**EF**	**SF**	**EF**	**SF**	**EF**	**SF**	**EF**	**SF**	**EF**	**SF**	**EF**	**SF**	**EF**	**SF**
D1	51.4	54.9	48	54.6	47.6	55.4	53	57	55.4	**62.1**	53	58.5	51.5	55.9	50.2	59.9	58.5	60.6
D2	52	59.4	49.3	54.0	48	53.9	46	52.61	53	59.9	59.8	**63.9**	52	56.7	54.6	61.3	53.6	59.8
D3	54.2	59.5	48.2	51.2	51.2	56.3	53	55.5	52.2	56.5	59.3	**65.2**	46.8	50.6	52.2	56.7	56.4	60.3
D4	53.5	55.9	46.8	49.9	52.2	55.7	51.2	56.6	50.3	53.4	56.2	59.9	46.6	50.2	51.3	53.6	54.6	**60.5**
D5	50.2	53.4	42.5	47.2	52.3	56.5	45.5	52.6	51.3	57.5	54.3	59.9	46.6	52.2	53.4	54.6	55.5	**61.3**
D6	50	53	51	59.9	46	52	45	54	50	54	52	**61.1**	46	49.9	51	54	53	55
D7	44	49	53	**59.9**	51	56	40	45	51	59	50	55	42	46	50	53	52	55
D8	44	53	46	49.9	52	59	54	59	53	59	55	**61.1**	42	45	52	55	56	60.1
D9	44	52	44	49.9	50	54	51	54	53	55	55	65	51	56	53	60	59	**66**
D10	44	52	42	49.9	54	59	51	59	42	56	54	**64.4**	46	55	51	59	55	64
D11	46	59	46	49.9	51	52	40	55	52	62	49	54.1	41	52	54	62	56	**66**
D12	45	59	44	52.3	41	55	40	54	42	56	49	55.1	42	50	52	56	46	**60.0**
D13	43	59	56	60.2	41	56	46	55	42	56	49	**64.1**	45	53	50	56	51	62
D14	50.6	56.8	46.9	59.2	44.3	58.4	55.4	62.8	54.8	61.9	48.5	**62.1**	42.4	57.8	54.3	61.3	52.8	61.9
D15	51.2	57.3	57.2	59.2	51.8	60.9	43.5	55.9	52.9	56.9	52.8	61.8	56.9	61.3	54.9	58.9	58.9	**65.9**
D16	44.2	59.3	46.1	49.3	51.2	56.8	42.1	59.5	58.1	**66.9**	51.2	63.1	46.8	50.8	52.2	56.9	51.2	62.4
D17	44.4	58.9	46.3	53.9	50.3	61.2	50.9	55.8	43	56.9	51.8	**63.9**	46.8	54.8	42.3	61.3	46.7	62.8
D18	44.2	52.3	46.1	52.9	44.8	56.8	54	62	53	62	59.8	64.3	56.8	61.2	53	61.8	58	**65.8**
D19	43	59.2	56.2	58.94	53.2	59.3	48	57.3	52	58.3	53	62.9	46.8	53.8	52	58.1	53	**66.1**
D20	49	54.1	54.2	62.6	51.9	62.1	48	52.2	53	59.3	48	**61.9**	53	61.2	53	59.8	53	61.9
D21	54.2	61.2	43	58.9	52	57.2	49	53.1	52	59.8	53	**63.2**	56.9	59.8	43.1	58.1	49	55.2
D22	46	52.2	49	59.2	53	59.2	43	50.2	53	55.9	51	62	56	62	52	61	56	**63.9**
D23	44	55	46	59	51	58	45	59	42	56	49	60	40	55	42	56	46	**63**
D24	54	58	46	59.9	51	56	50	59	45	56	59	**62.3**	46	55	52	62	56	62
D25	45	59	46	55	55	61	49	52	50	59	52	**62**	51	62.8	52	61	56	61.5
D26	40	52	46	55	50	61	50	59	52	56	59	**64**	56	60	52	61	58	62.5
D27	44	59	46	51.2	50	55	50	55	50	55	52	61.5	50	59	53	61	57.8	**62.6**
D28	54	59	51	59.8	51	60.1	50	61.5	52	60.2	59	**63.7**	56	59	53	61.5	59	63.5
D29	44	52.6	41	52.2	41	56.4	50.4	55.5	52.5	59.6	51	66.1	46	50.5	55.9	60.9	51	**66.4**
D30	53	59.8	52.2	59.2	51	58	50	55	52	56	59	**64.1**	53	58	52	56.3	56.9	62.2

Table S4: F-Measure measured by ULR analysis.

ID	GD		GDM		GDA		NM		LM		DNN		BTE		MVE		NDTF	
	**EF**	**SF**	**EF**	**SF**	**EF**	**SF**	**EF**	**SF**	**EF**	**SF**	**EF**	**SF**	**EF**	**SF**	**EF**	**SF**	**EF**	**SF**
D1	0.60	0.68	0.58	0.591	0.52	0.53	0.52	0.58	0.50	**0.59**	0.48	0.53	0.49	0.57	0.43	0.52	0.51	0.57
D2	0.44	0.57	0.52	0.57	0.50	0.56	0.48	0.56	0.47	**0.60**	0.51	0.57	0.47	0.59	0.47	0.58	0.47	0.59
D3	0.47	0.58	0.48	0.58	0.46	0.57	0.47	0.58	0.49	0.59	0.51	**0.58**	0.49	0.57	0.48	0.59	0.48	0.58
D4	0.44	0.57	0.49	0.58	0.47	0.57	0.47	0.59	0.49	0.58	0.48	0.57	0.49	0.58	0.48	0.59	0.49	**0.60**
D5	0.48	0.58	0.47	0.52	0.47	0.57	0.48	0.53	0.43	0.52	0.41	0.551	0.46	0.52	0.43	0.54	0.45	**0.59**
D6	0.48	0.54	0.48	0.57	0.48	0.53	0.46	0.53	0.41	0.52	0.42	**0.53**	0.46	0.521	0.42	0.524	0.44	0.52
D7	0.45	0.57	0.45	**0.59**	0.46	0.57	0.46	0.56	0.51	0.58	0.51	0.58	0.47	0.58	0.51	0.58	0.49	0.56
D8	0.52	0.57	0.52	0.58	0.53	0.58	0.52	0.59	0.52	0.54	0.56	**0.62**	0.52	0.58	0.53	0.57	0.55	0.60
D9	0.49	0.58	0.49	0.59	0.47	0.53	0.48	0.58	0.47	0.58	0.48	0.59	0.57	0.59	0.48	0.59	0.49	**0.60**
D10	0.49	0.58	0.47	0.57	0.48	0.58	0.47	0.57	0.47	0.58	0.47	**0.59**	0.46	0.57	0.45	0.54	0.45	0.57
D11	0.45	0.54	0.43	0.53	0.46	0.53	0.46	0.53	0.46	0.53	0.48	0.54	0.44	0.52	0.51	0.56	0.53	**0.59**
D12	0.46	0.52	0.45	0.52	0.48	0.51	0.51	0.53	0.53	0.57	0.48	0.53	0.51	0.59	0.522	0.56	0.56	**0.60**
D13	0.52	0.62	0.53	0.58	0.45	0.51	0.45	0.51	0.51	0.55	0.52	**0.64**	0.53	0.62	0.50	0.61	0.51	0.62
D14	0.51	0.55	0.47	0.56	0.52	0.58	0.53	0.58	0.51	0.54	0.55	**0.62**	0.54	0.62	0.54	0.60	0.49	0.59
D15	0.52	0.58	0.45	0.58	0.51	0.57	0.46	0.51	0.51	0.57	0.51	0.61	0.56	0.59	0.45	0.56	0.55	**0.64**
D16	0.46	0.52	0.52	0.58	0.52	0.55	0.48	0.54	0.55	**0.61**	0.51	0.59	0.46	0.51	0.51	0.55	0.50	0.59
D17	0.51	0.58	0.45	0.50	0.51	0.57	0.51	0.57	0.48	0.53	0.59	**0.62**	0.56	0.58	0.56	0.59	0.57	0.61
D18	0.45	0.50	0.45	0.53	0.43	0.50	0.54	0.58	0.53	0.58	0.59	0.62	0.57	0.61	0.52	0.61	0.56	**0.63**
D19	0.48	0.51	0.51	0.60	0.56	0.61	0.51	0.61	0.44	0.52	0.52	0.6	0.49	0.54	0.45	0.51	0.54	**0.61**
D20	0.48	0.53	0.45	0.55	0.56	0.58	0.57	0.61	0.51	0.55	0.51	**0.62**	0.514	0.61	0.45	0.52	0.52	0.59
D21	0.52	0.57	0.42	0.53	0.46	0.50	0.46	0.51	0.51	0.59	0.53	**0.62**	0.46	0.59	0.41	0.50	0.41	0.55
D22	0.51	0.55	0.52	0.58	0.48	0.55	0.53	0.58	0.40	0.50	0.51	0.59	0.46	0.52	0.50	0.56	0.51	**0.61**
D23	0.53	0.58	0.55	0.60	0.50	0.57	0.50	0.58	0.52	0.56	0.49	0.56	0.54	0.59	0.52	0.56	0.56	**0.60**
D24	0.48	0.52	0.47	0.53	0.52	0.55	0.46	0.56	0.46	0.52	0.51	**0.61**	0.41	0.50	0.52	0.59	0.51	0.59
D25	0.50	0.53	0.47	0.50	0.53	0.55	0.51	0.56	0.51	0.58	0.50	**0.61**	0.51	0.56	0.52	0.59	0.46	0.55
D26	0.41	0.51	0.40	0.56	0.41	0.58	0.52	0.57	0.53	0.57	0.59	**0.63**	0.56	0.60	0.52	0.56	0.56	0.62
D27	0.47	0.54	0.55	0.61	0.51	0.54	0.50	0.58	0.46	0.55	0.50	0.59	0.40	0.55	0.41	0.56	0.55	**0.63**
D28	0.47	0.58	0.52	0.60	0.50	0.58	0.44	0.58	0.51	0.59	0.51	**0.61**	0.51	0.61	0.52	0.58	0.52	0.59
D29	0.51	0.57	0.53	0.60	0.40	0.53	0.43	0.50	0.43	0.53	0.41	0.56	0.46	0.55	0.52	0.59	0.51	**0.61**
D30	0.47	0.51	0.47	0.58	0.50	0.55	0.46	0.55	0.52	0.54	0.51	**0.61**	0.49	0.55	0.51	0.55	0.56	0.60

Table S5: Accuracy measured by using Cross correlation analysis.

ID	GD		GDM		GDA		NM		LM		DNN		BTE		MVE		NDTF
	**EF**	**SF**	**EF**	**SF**	**EF**	**SF**	**EF**	**SF**	**EF**	**SF**	**EF**	**SF**	**EF**	**SF**	**EF**	**SF**	**EF**	**SF**
D1	53.4	57.9	48	54.6	47.6	55.4	53	57	57.4	**63.1**	53	58.5	51.5	55.9	50.2	59.9	58.5	60.6
D2	53.2	59.4	49.3	54.0	48	53.9	46	52.61	53	59.9	59.8	**62.3**	52	56.7	54.6	61.3	53.6	59.8
D3	54.2	59.5	48.2	51.2	51.2	56.3	53	55.5	52.2	56.5	59.3	**64.2**	46.8	50.6	52.2	56.7	56.4	60.3
D4	53.5	55.9	46.8	49.9	52.2	55.7	51.2	56.6	50.3	53.4	56.2	59.9	46.6	50.2	51.3	53.6	54.6	**62.5**
D5	50.2	53.4	42.5	47.2	52.3	56.5	45.5	52.6	51.3	57.5	54.3	59.9	46.6	52.2	53.4	54.6	55.5	**62.3**
D6	53	59	51	59.9	46	52	45	54	50	54	52	**62.1**	46	49.9	51	54	53	55
D7	49	59	53	**60.9**	51	56	40	45	51	59	50	55	42	46	50	53	52	55
D8	49	53	46	49.9	52	59	54	59	53	59	55	**61.9**	42	45	52	55	56	60.1
D9	54	59.8	44	49.9	50	54	51	54	53	55	55	65	51	56	53	60	59	**66**
D10	56	59	42	49.9	54	59	51	59	42	56	54	**64.4**	46	55	51	59	55	64
D11	56	59.9	46	49.9	51	52	40	55	52	62	49	54.1	41	52	54	62	56	**66**
D12	55.2	59	44	52.3	41	55	40	54	42	56	49	55.1	42	50	52	56	46	**60.0**
D13	53.8	59	56	60.2	41	56	46	55	42	56	49	**64.1**	45	53	50	56	51	62
D14	50.6	56.8	46.9	59.2	44.3	58.4	55.4	62.8	54.8	61.9	48.5	**62.1**	42.4	57.8	54.3	61.3	52.8	61.9
D15	51.2	57.3	57.2	59.2	51.8	60.9	43.5	55.9	52.9	56.9	52.8	61.8	56.9	61.3	54.9	58.9	58.9	**65.9**
D16	44.2	59.3	46.1	49.3	51.2	56.8	42.1	59.5	58.1	**66.9**	51.2	63.1	46.8	50.8	52.2	56.9	51.2	62.4
D17	44.4	58.9	46.3	53.9	50.3	61.2	50.9	55.8	43	56.9	51.8	**63.9**	46.8	54.8	42.3	61.3	46.7	62.8
D18	44.2	52.3	46.1	52.9	44.8	56.8	54	62	53	62	59.8	64.3	56.8	61.2	53	61.8	58	**65.8**
D19	43	59.2	56.2	58.94	53.2	59.3	48	57.3	52	58.3	53	62.9	46.8	53.8	52	58.1	53	**66.1**
D20	49	54.1	54.2	62.6	51.9	62.1	48	52.2	53	59.3	48	**61.9**	53	61.2	53	59.8	53	61.9
D21	54.2	61.2	43	58.9	52	57.2	49	53.1	52	59.8	53	**63.2**	56.9	59.8	43.1	58.1	49	55.2
D22	46	52.2	49	59.2	53	59.2	43	50.2	53	55.9	51	62	56	62	52	61	56	**63.9**
D23	44	55	46	59	51	58	45	59	42	56	49	60	40	55	42	56	46	**63**
D24	54	58	46	59.9	51	56	50	59	45	56	59	**62.3**	46	55	52	62	56	62
D25	45	59	46	55	55	61	49	52	50	59	52	**62**	51	62.8	52	61	56	61.5
D26	40	52	46	55	50	61	50	59	52	56	59	**64**	56	60	52	61	58	62.5
D27	44	59	46	51.2	50	55	50	55	50	55	52	61.5	50	59	53	61	57.8	**62.6**
D28	54	59	51	59.8	51	60.1	50	61.5	52	60.2	59	**63.7**	56	59	53	61.5	59	63.5
D29	44	52.6	41	52.2	41	56.4	50.4	55.5	52.5	59.6	51	66.1	46	50.5	55.9	60.9	51	**66.4**
D30	53	59.8	52.2	59.2	51	58	50	55	52	56	59	**64.1**	53	58	52	56.3	56.9	62.2

Table S6: F-Measure measured by using Cross correlation analysis.

**ID**	GD		GDM		GDA		NM		LM		DNN		BTE		MVE		NDTF	
	**EF**	**SF**	**EF**	**SF**	**EF**	**SF**	**EF**	**SF**	**EF**	**SF**	**EF**	**SF**	**EF**	**SF**	**EF**	**SF**	**EF**	**SF**
D1	0.52	0.57	0.51	0.58	0.50	0.58	0.52	0.58	0.50	**0.62**	0.50	0.55	0.51	0.57	0.50	0.58	0.51	0.57
D2	0.52	0.57	0.51	0.57	0.50	0.56	0.51	0.58	0.52	**0.61**	0.51	0.57	0.51	0.59	0.50	0.58	0.51	0.59
D3	0.52	0.58	0.53	0.58	0.51	0.57	0.52	0.57	0.51	0.58	0.50	**0.60**	0.52	0.59	0.51	0.59	0.51	0.58
D4	0.52	0.57	0.52	0.58	0.51	0.57	0.50	0.58	0.51	0.57	0.50	0.56	0.50	0.58	0.50	0.59	0.51	**0.60**
D5	0.51	0.58	0.51	0.56	0.50	0.57	0.51	0.58	0.51	0.56	0.50	0.59	0.51	0.59	0.53	0.55	0.55	**0.61**
D6	0.51	0.54	0.50	0.55	0.55	0.58	0.50	0.57	0.50	0.58	0.57	**0.63**	0.56	0.59	0.52	0.58	0.55	0.60
D7	0.51	0.57	0.55	**0.58**	0.52	0.57	0.50	0.55	0.50	0.57	0.51	0.58	0.50	0.58	0.51	0.58	0.50	0.59
D8	0.52	0.57	0.53	0.58	0.54	0.58	0.52	0.58	0.51	0.56	0.55	**0.61**	0.52	0.58	0.53	0.57	0.55	0.60
D9	0.50	0.57	0.51	0.58	0.50	0.57	0.50	0.58	0.51	0.58	0.53	0.59	0.54	0.59	0.52	0.59	0.59	**0.60**
D10	0.52	0.58	0.50	0.57	0.52	0.58	0.50	0.57	0.51	0.58	0.52	**0.60**	0.52	0.57	0.51	0.58	0.51	0.59
D11	0.50	0.55	0.50	0.56	0.50	0.51	0.51	0.53	0.50	0.57	0.51	0.57	0.51	0.59	0.51	0.56	0.53	**0.61**
D12	0.50	0.56	0.50	0.57	0.50	0.57	0.51	0.54	0.51	0.58	0.50	0.57	0.50	0.59	0.52	0.56	0.56	**0.60**
D13	0.52	0.60	0.51	0.58	0.50	0.58	0.50	0.57	0.50	0.55	0.52	**0.65**	0.53	0.62	0.50	0.61	0.51	0.62
D14	0.51	0.55	0.50	0.56	0.52	0.58	0.54	0.58	0.51	0.54	0.54	**0.62**	0.55	0.61	0.55	0.60	0.59	0.59
D15	0.52	0.58	0.55	0.58	0.51	0.55	0.54	0.58	0.51	0.55	0.50	0.60	0.55	0.59	0.55	0.56	0.55	**0.65**
D16	0.50	0.57	0.52	0.58	0.50	0.55	0.50	0.54	0.55	**0.62**	0.51	0.59	0.51	0.59	0.51	0.55	0.50	0.59
D17	0.51	0.58	0.55	0.50	0.51	0.57	0.51	0.57	0.51	0.54	0.50	**0.62**	0.52	0.58	0.51	0.59	0.57	0.61
D18	0.51	0.58	0.53	0.57	0.50	0.58	0.50	0.58	0.56	0.58	0.52	0.62	0.57	0.61	0.52	0.61	0.56	**0.63**
D19	0.50	0.58	0.51	0.60	0.56	0.61	0.51	0.61	0.54	0.51	0.51	0.6	0.51	0.58	0.55	0.59	0.55	**0.61**
D20	0.50	0.56	0.51	0.58	0.50	0.58	0.52	0.61	0.51	0.55	0.50	**0.62**	0.515	0.61	0.55	0.61	0.52	0.59
D21	0.51	0.56	0.51	0.52	0.51	0.55	0.51	0.56	0.52	0.57	0.51	**0.62**	0.561	0.59	0.51	0.58	0.51	0.55
D22	0.52	0.56	0.51	0.57	0.51	0.55	0.53	0.57	0.51	0.51	0.50	0.59	0.51	0.58	0.50	0.56	0.51	**0.61**
D23	0.54	0.58	0.57	0.60	0.52	0.59	0.52	0.58	0.53	0.55	0.58	0.56	0.55	0.59	0.52	0.56	0.56	**0.60**
D24	0.50	0.56	0.52	0.57	0.52	0.55	0.50	0.58	0.56	0.58	0.51	**0.61**	0.51	0.59	0.52	0.59	0.51	0.59
D25	0.50	0.56	0.50	0.59	0.53	0.55	0.51	0.58	0.53	0.58	0.53	**0.61**	0.51	0.56	0.52	0.59	0.56	0.60
D26	0.51	0.53	0.52	0.54	0.51	0.58	0.54	0.57	0.53	0.57	0.58	**0.63**	0.56	0.60	0.52	0.56	0.56	0.62
D27	0.57	0.56	0.57	0.61	0.51	0.54	0.52	0.58	0.54	0.55	0.52	0.58	0.50	0.55	0.51	0.56	0.55	**0.63**
D28	0.52	0.58	0.52	0.60	0.52	0.58	0.56	0.58	0.51	0.58	0.50	**0.61**	0.51	0.61	0.52	0.58	0.52	0.59
D29	0.53	0.57	0.56	0.61	0.50	0.54	0.54	0.50	0.54	0.54	0.50	0.56	0.56	0.55	0.52	0.59	0.51	**0.61**
D30	0.57	0.51	0.55	0.58	0.50	0.55	0.54	0.54	0.50	0.54	0.53	**0.63**	0.59	0.55	0.51	0.55	0.56	0.60

Table S7: Accuracy measured by using Multivariate linear regression stepwise forward selection.

**ID**	GD		GDM		GDA		NM		LM		DNN		BTE		MVE		NDTF	
	**EF**	**SF**	**EF**	**SF**	**EF**	**SF**	**EF**	**SF**	**EF**	**SF**	**EF**	**SF**	**EF**	**SF**	**EF**	**SF**	**EF**	**SF**
D1	53.4	57.9	48	54.6	47.6	55.4	53	57	57.4	**63.1**	53	58.5	51.5	55.9	50.2	59.9	58.5	60.6
D2	53.2	59.4	49.3	54.0	48	53.9	46	52.61	53	59.9	59.8	**62.3**	52	56.7	54.6	61.3	53.6	59.8
D3	54.2	59.5	48.2	51.2	51.2	56.3	53	55.5	52.2	56.5	59.3	**64.2**	46.8	50.6	52.2	56.7	56.4	60.3
D4	53.5	55.9	46.8	49.9	52.2	55.7	51.2	56.6	50.3	53.4	56.2	59.9	46.6	50.2	51.3	53.6	54.6	**62.5**
D5	50.2	53.4	42.5	47.2	52.3	56.5	45.5	52.6	51.3	57.5	54.3	59.9	46.6	52.2	53.4	54.6	55.5	**62.3**
D6	53	59	51	59.9	46	52	45	54	50	54	52	**62.1**	46	49.9	51	54	53	55
D7	49	59	53	**60.9**	51	56	40	45	51	59	50	55	42	46	50	53	52	55
D8	49	53	46	49.9	52	59	54	59	53	59	55	**61.9**	42	45	52	55	56	60.1
D9	54	59.8	44	49.9	50	54	51	54	53	55	55	65	51	56	53	60	59	**66**
D10	56	59	42	49.9	54	59	51	59	42	56	54	**64.4**	46	55	51	59	55	64
D11	56	59.9	46	49.9	51	52	40	55	52	62	49	54.1	41	52	54	62	56	**66**
D12	55.2	59	44	52.3	41	55	40	54	42	56	49	55.1	42	50	52	56	46	**60.0**
D13	53.8	59	56	60.2	41	56	46	55	42	56	49	**64.1**	45	53	50	56	51	62
D14	50.6	56.8	46.9	59.2	44.3	58.4	55.4	62.8	54.8	61.9	48.5	**62.1**	42.4	57.8	54.3	61.3	52.8	61.9
D15	51.2	57.3	57.2	59.2	51.8	60.9	43.5	55.9	52.9	56.9	52.8	61.8	56.9	61.3	54.9	58.9	58.9	**65.9**
D16	54.2	59.3	46.1	49.3	51.2	56.8	42.1	59.5	58.1	**67.9**	51.2	63.1	46.8	50.8	52.2	56.9	51.2	62.4
D17	54.4	58.9	46.3	53.9	50.3	61.2	50.9	55.8	43	56.9	51.8	**64.9**	46.8	54.8	42.3	61.3	46.7	62.8
D18	54.2	58.3	46.1	52.9	44.8	56.8	54	62	53	62	59.8	64.3	56.8	61.2	53	61.8	58	**65.8**
D19	43	59.2	56.2	58.94	53.2	59.3	48	57.3	52	58.3	53	62.9	46.8	53.8	52	58.1	53	**66.1**
D20	50.3	54.1	54.2	62.6	51.9	62.1	48	52.2	53	59.3	48	**61.9**	53	61.2	53	59.8	53	61.9
D21	54.2	61.2	43	58.9	52	57.2	49	53.1	52	59.8	53	**63.2**	56.9	59.8	43.1	58.1	49	55.2
D22	51.2	57.2	49	59.2	53	59.2	43	50.2	53	55.9	51	62	56	62	52	61	56	**63.9**
D23	54	59.8	46	59	51	58	45	59	42	56	49	60	40	55	42	56	46	**63**
D24	54	58	46	59.9	51	56	50	59	45	56	59	**62.3**	46	55	52	62	56	62
D25	45	59	46	55	55	61	49	52	50	59	52	**62**	51	62.8	52	61	56	61.5
D26	49.9	53.9	46	55	50	61	50	59	52	56	59	**64**	56	60	52	61	58	62.5
D27	52.3	59	46	51.2	50	55	50	55	50	55	52	61.5	50	59	53	61	57.8	**62.6**
D28	54	59	51	59.8	51	60.1	50	61.5	52	60.2	59	**63.7**	56	59	53	61.5	59	63.5
D29	51.8	59.6	41	52.2	41	56.4	50.4	55.5	52.5	59.6	51	66.1	46	50.5	55.9	60.9	51	**66.4**
D30	53	59.8	52.2	59.2	51	58	50	55	52	56	59	**64.1**	53	58	52	56.3	56.9	62.2

Table S8: F-Measure measured by using Multivariate linear regression stepwise forward selection.

**ID**	GD		GDM		GDA		NM		LM		DNN		BTE		MVE		NDTF
	**EF**	**SF**	**EF**	**SF**	**EF**	**SF**	**EF**	**SF**	**EF**	**SF**	**EF**	**SF**	**EF**	**SF**	**EF**	**SF**	**EF**	**SF**
D1	0.52	0.57	0.51	0.58	0.52	0.58	0.52	0.58	0.52	**0.62**	0.50	0.55	0.51	0.57	0.50	0.58	0.51	0.57
D2	0.52	0.57	0.51	0.57	0.52	0.56	0.51	0.58	0.50	**0.62**	0.51	0.57	0.51	0.59	0.50	0.58	0.51	0.59
D3	0.50	0.58	0.53	0.58	0.51	0.57	0.52	0.57	0.51	0.58	0.50	**0.60**	0.52	0.59	0.51	0.59	0.51	0.58
D4	0.52	0.57	0.52	0.58	0.53	0.57	0.52	0.58	0.51	0.57	0.50	0.57	0.50	0.58	0.50	0.59	0.51	**0.60**
D5	0.51	0.58	0.51	0.56	0.52	0.57	0.53	0.58	0.51	0.56	0.52	0.59	0.51	0.59	0.53	0.55	0.55	**0.61**
D6	0.51	0.56	0.50	0.55	0.55	0.58	0.52	0.59	0.52	0.58	0.57	**0.63**	0.56	0.59	0.52	0.58	0.55	0.60
D7	0.50	0.59	0.57	**0.59**	0.50	0.57	0.52	0.55	0.50	0.57	0.51	0.58	0.50	0.58	0.51	0.58	0.50	0.59
D8	0.52	0.57	0.52	0.58	0.56	0.58	0.52	0.58	0.51	0.56	0.55	**0.62**	0.52	0.58	0.53	0.57	0.55	0.60
D9	0.50	0.57	0.51	0.58	0.52	0.57	0.50	0.58	0.51	0.58	0.52	0.59	0.54	0.59	0.52	0.59	0.59	**0.60**
D10	0.52	0.58	0.52	0.57	0.52	0.58	0.52	0.57	0.53	0.58	0.52	**0.60**	0.52	0.57	0.51	0.58	0.51	0.59
D11	0.50	0.55	0.52	0.58	0.50	0.51	0.51	0.53	0.52	0.57	0.51	0.57	0.50	0.59	0.51	0.56	0.53	**0.61**
D12	0.52	0.56	0.52	0.57	0.52	0.57	0.52	0.54	0.53	0.58	0.50	0.57	0.50	0.59	0.52	0.56	0.56	**0.60**
D13	0.52	0.60	0.53	0.58	0.52	0.58	0.52	0.57	0.51	0.57	0.53	**0.65**	0.53	0.62	0.50	0.61	0.51	0.62
D14	0.53	0.55	0.52	0.56	0.52	0.58	0.56	0.58	0.51	0.54	0.54	**0.62**	0.55	0.61	0.55	0.60	0.59	0.59
D15	0.52	0.58	0.57	0.58	0.53	0.57	0.56	0.58	0.53	0.57	0.52	0.61	0.56	0.59	0.55	0.56	0.55	**0.65**
D16	0.48	0.52	0.52	0.58	0.52	0.55	0.48	0.54	0.55	**0.61**	0.51	0.59	0.46	0.51	0.51	0.55	0.50	0.59
D17	0.51	0.58	0.47	0.52	0.53	0.57	0.51	0.57	0.47	0.53	0.58	**0.62**	0.56	0.58	0.56	0.59	0.57	0.61
D18	0.47	0.5	0.47	0.53	0.45	0.52	0.54	0.58	0.53	0.58	0.58	0.62	0.57	0.61	0.52	0.61	0.56	**0.63**
D19	0.48	0.51	0.51	0.60	0.56	0.61	0.51	0.6	0.47	0.53	0.53	0.6	0.49	0.54	0.45	0.51	0.54	**0.61**
D20	0.48	0.53	0.45	0.55	0.58	0.58	0.57	0.61	0.51	0.55	0.52	**0.62**	0.514	0.61	0.45	0.52	0.52	0.59
D21	0.52	0.57	0.42	0.51	0.46	0.52	0.46	0.51	0.51	0.58	0.52	**0.62**	0.46	0.59	0.41	0.50	0.41	0.55
D22	0.50	0.56	0.51	0.59	0.49	0.56	0.54	0.59	0.41	0.51	0.51	0.58	0.46	0.52	0.50	0.56	0.51	**0.61**
D23	0.53	0.58	0.55	0.59	0.50	0.57	0.52	0.58	0.53	0.57	0.48	0.55	0.54	0.59	0.52	0.56	0.56	**0.60**
D24	0.48	0.52	0.47	0.53	0.50	0.55	0.46	0.54	0.46	0.50	0.51	**0.61**	0.41	0.50	0.52	0.59	0.51	0.59
D25	0.52	0.53	0.47	0.52	0.51	0.55	0.51	0.58	0.51	0.58	0.50	**0.61**	0.51	0.56	0.52	0.59	0.46	0.55
D26	0.41	0.51	0.42	0.54	0.41	0.58	0.54	0.57	0.53	0.55	0.58	**0.63**	0.56	0.60	0.52	0.56	0.56	0.62
D27	0.47	0.54	0.55	0.55	0.51	0.54	0.52	0.58	0.47	0.55	0.50	0.59	0.40	0.55	0.41	0.56	0.55	**0.63**
D28	0.47	0.58	0.52	0.60	0.50	0.58	0.46	0.58	0.51	0.59	0.51	**0.61**	0.51	0.61	0.52	0.58	0.52	0.59
D29	0.53	0.57	0.53	0.58	0.42	0.55	0.45	0.52	0.45	0.54	0.41	0.56	0.46	0.55	0.52	0.59	0.51	**0.61**
D30	0.47	0.51	0.47	0.58	0.52	0.55	0.47	0.53	0.52	0.54	0.51	**0.61**	0.49	0.55	0.51	0.55	0.56	0.60

Table S9: Accuracy measured by using t-test and ULR analysis.

**ID**	GD		GDM		GDA		NM		LM		DNN		BTE		MVE		NDTF	
	**EF**	**SF**	**EF**	**SF**	**EF**	**SF**	**EF**	**SF**	**EF**	**SF**	**EF**	**SF**	**EF**	**SF**	**EF**	**SF**	**EF**	**SF**
D1	63.4	67.9	58	64.6	57.6	66.4	63	67	67.4	**69.9**	63	68.6	61.6	66.9	60.2	69.3	64.6	69.6
D2	63.2	69.4	59.3	64.0	58	63.9	56	62.61	63	69.9	59.8	**62.3**	62	66.7	59.6	61.3	63.6	69.8
D3	64.2	69.6	58.9	61.2	61.2	66.3	63	66.6	62.2	66.6	59.9	**64.2**	56.8	60.6	62.2	66.7	60.4	66.3
D4	63.6	66.9	56.8	60.9	62.2	66.7	61.2	66.6	60.3	63.4	66.2	69.9	56.6	60.2	61.3	63.6	61.6	**67.6**
D6	60.2	63.4	52.6	60.2	62.3	66.6	56.6	62.6	61.3	67.6	61.3	69.9	56.6	62.2	60.4	64.6	61.6	**66.3**
D6	63	69	61	69.9	56	62	56	64	60	64	59.9	**65.1**	56	59.9	61	64	63	64.8
D7	59	69	63	**65.9**	61	64	55	60	61	69	60	66	58	61.9	60	63	62	65
D8	59	63	56	60.9	62	69	64	69	63	69	66	**69.9**	52	66	62	66	66	68.1
D9	64	69.8	58	61.9	60	64	61	64	63	66	62	66	61	66	63	67	66	**69.9**
D10	56	62	54	61.9	64	69	61	69	52	66	64	**69.4**	56	66	61	69	62	68.9
D11	66	69.9	56	60.9	61	62.8	56.9	66.2	59.2	62.2	57.9	64.1	58.9	62.2	61.1	68.1	62.1	**69.1**
D12	66.2	69	58.1	62.3	58.1	66.1	53.2	64.1	56.2	66.1	59.1	66.1	58.1	60	62	66	57.6	**67.9**
D13	63.8	69.1	61.1	68.2	56.9	66.1	56	66.1	56.2	66.1	59.1	**67.1**	56.9	63.1	60.2	66	61.1	62.3
D14	60.6	66.8	59.9	62.2	59.3	68.4	60.4	68.8	61.8	67.9	58.6	**69.2**	56.4	67.8	54.3	63.3	52.8	64.9
D15	61.2	67.3	67.2	69.2	61.8	63.9	59.6	66.9	62.9	66.9	62.8	67.8	66.9	69.3	64.9	68.9	66.9	**69.9**
D16	58.2	69.3	56.1	60.3	61.2	66.8	57.1	61.6	60.9	**66.9**	61.7	63.9	56.8	60.8	61.2	65.9	61.2	66.4
D17	59.4	68.9	56.3	63.9	60.3	64.2	60.9	66.8	59.9	66.9	61.8	**67.9**	56.8	64.8	58.3	63.3	59.7	67.8
D18	56.2	62.3	56.8	62.9	58.8	66.8	61	68.1	60.8	67.9	61.8	67.3	62.8	68.2	63	68.8	63.1	**69.8**
D19	58.9	68.9	61.2	68.9	63.2	69.3	58.9	67.3	62	68.3	63	68.9	59.8	65.8	62	68.1	63.1	**69.9**
D20	59.2	64.1	60.2	66.6	61.9	68.1	58.8	66.2	63.1	69.3	58.9	**69.9**	63	67.2	63	69.8	63.8	69.3
D21	61.2	67.2	58.9	68.9	62	67.2	58.9	63.1	62.1	69.8	63	**69.2**	66.9	69.1	58.8	68.1	59.2	68.2
D22	58.1	62.2	59.2	69.2	63.3	69.2	57.3	64.2	63.1	66.9	61.2	67.1	60.8	68.1	62.2	67.6	62	**69.9**
D23	59.1	66.2	58.6	69.1	61	68.2	58.6	69.1	59.8	66.8	59.2	67.1	58.9	66.2	58.9	66.8	59.8	**69.8**
D24	64.2	68.7	60.2	69.9	61	66.9	60.7	69.1	56.9	66.7	61.9	**69.3**	59.6	66.1	62	68.1	61	68.2
D25	59.0	69	58.9	66.1	59.6	63.9	58.9	68.2	60.8	69.2	62	**69.8**	61	68.8	62	68.7	62	69.6
D26	58.9	62.8	58.6	66.2	60	68.7	62	69.1	62	68.9	61	**69.8**	61	67.8	62	69.8	61	69.7
D27	58.0	69.2	57.8	68.2	60	66.9	60	66.8	60	66.9	62	67.6	60	69.1	63	68.9	61.8	**69.6**
D28	64	69	61	69.8	61	67.1	60	68.6	62	68.2	63	**69.7**	66	69	63	69.6	62	68.6
D29	58.9	62.6	58.9	62.2	58.9	66.4	60.4	66.6	62.6	69.6	61	66.1	59.6	62.6	61.9	68.9	61.8	**69.9**
D30	63	69.8	62.2	69.2	61	68	60	66	62	66	63	**69.1**	63	68	62	66.3	61.9	69.0

Table S10: F-Measure measured by using t-test and ULR analysis.

ID	GD		GDM		GDA		NM		LM		DNN		BTE		MVE		NDTF	
	**EF**	**SF**	**EF**	**SF**	**EF**	**SF**	**EF**	**SF**	**EF**	**SF**	**EF**	**SF**	**EF**	**SF**	**EF**	**SF**	**EF**	**SF**
D1	0.60	0.67	0.61	0.68	0.6	0.67	0.60	0.68	0.6	**0.71**	0.60	0.66	0.61	0.67	0.60	0.68	0.61	0.67
D2	0.62	0.67	0.61	0.67	0.62	0.66	0.61	0.66	0.6	**0.71**	0.61	0.67	0.61	0.69	0.60	0.68	0.61	0.69
D3	0.60	0.68	0.61	0.68	0.61	0.67	0.6	0.67	0.61	0.68	0.61	**0.70**	0.62	0.69	0.61	0.69	0.61	0.68
D4	0.62	0.67	0.64	0.68	0.63	0.67	0.6	0.68	0.61	0.67	0.6	0.66	0.60	0.68	0.60	0.69	0.61	**0.70**
D5	0.61	0.68	0.6	0.66	0.6	0.66	0.61	0.68	0.61	0.66	0.6	0.69	0.61	0.69	0.63	0.66	0.66	**0.71**
D6	0.61	0.65	0.6	0.66	0.63	0.68	0.6	0.67	0.6	0.68	0.67	**0.73**	0.67	0.69	0.62	0.68	0.66	0.70
D7	0.60	0.67	0.67	**0.69**	0.6	0.67	0.6	0.66	0.6	0.67	0.61	0.68	0.60	0.68	0.61	0.68	0.60	0.68
D8	0.62	0.67	0.62	0.68	0.65	0.68	0.62	0.68	0.61	0.67	0.66	**0.72**	0.62	0.68	0.63	0.67	0.66	0.70
D9	0.6	0.67	0.61	0.68	0.6	0.67	0.6	0.68	0.61	0.68	0.62	0.69	0.64	0.69	0.62	0.69	0.63	**0.70**
D10	0.62	0.68	0.6	0.67	0.62	0.68	0.6	0.67	0.61	0.68	0.62	**0.70**	0.62	0.67	0.61	0.68	0.61	0.69
D11	0.6	0.66	0.62	0.66	0.6	0.68	0.61	0.63	0.6	0.67	0.61	0.68	0.61	0.69	0.61	0.67	0.63	**0.71**
D12	0.6	0.66	0.62	0.67	0.6	0.67	0.61	0.65	0.61	0.68	0.60	0.67	0.61	0.69	0.62	0.67	0.67	**0.70**
D13	0.62	0.70	0.61	0.68	0.60	0.68	0.60	0.67	0.61	0.66	0.62	**0.76**	0.63	0.72	0.60	0.71	0.61	0.72
D14	0.61	0.66	0.6	0.65	0.62	0.68	0.65	0.68	0.61	0.65	0.65	**0.72**	0.66	0.71	0.66	0.70	0.69	0.69
D15	0.62	0.68	0.66	0.68	0.61	0.68	0.67	0.68	0.63	0.66	0.62	0.70	0.67	0.69	0.66	0.67	0.66	**0.76**
D16	0.60	0.67	0.62	0.68	0.60	0.66	0.60	0.65	0.66	**0.71**	0.61	0.69	0.61	0.69	0.61	0.66	0.60	0.69
D17	0.61	0.68	0.61	0.67	0.61	0.67	0.61	0.67	0.61	0.65	0.60	**0.72**	0.62	0.68	0.61	0.69	0.67	0.71
D18	0.61	0.68	0.61	0.67	0.62	0.68	0.62	0.68	0.67	0.68	0.62	0.70	0.67	0.71	0.62	0.71	0.67	**0.73**
D19	0.60	0.68	0.61	0.70	0.66	0.7	0.61	0.7	0.65	0.61	0.61	0.7	0.68	0.67	0.66	0.69	0.66	**0.71**
D20	0.61	0.66	0.63	0.68	0.65	0.68	0.64	0.7	0.62	0.66	0.65	**0.72**	0.616	0.71	0.66	0.71	0.62	0.69
D21	0.62	0.67	0.62	0.63	0.62	0.66	0.6	0.67	0.61	0.69	0.63	**0.72**	0.671	0.69	0.61	0.68	0.61	0.66
D22	0.61	0.65	0.6	0.66	0.6	0.66	0.65	0.68	0.6	0.62	0.60	0.69	0.61	0.68	0.60	0.67	0.61	**0.71**
D23	0.63	0.68	0.66	0.7	0.62	0.67	0.6	0.67	0.63	0.67	0.69	0.67	0.66	0.69	0.62	0.67	0.67	**0.70**
D24	0.62	0.65	0.62	0.66	0.62	0.65	0.6	0.66	0.65	0.69	0.60	**0.71**	0.61	0.69	0.62	0.69	0.61	0.69
D25	0.62	0.65	0.62	0.67	0.61	0.66	0.63	0.66	0.61	0.69	0.62	**0.71**	0.61	0.67	0.62	0.69	0.67	0.70
D26	0.61	0.67	0.6	0.65	0.61	0.67	0.62	0.66	0.61	0.66	0.68	**0.73**	0.67	0.70	0.62	0.67	0.67	0.72
D27	0.60	0.65	0.66	0.69	0.62	0.65	0.60	0.68	0.65	0.66	0.60	0.69	0.60	0.66	0.61	0.67	0.66	**0.73**
D28	0.60	0.68	0.62	0.7	0.62	0.68	0.65	0.67	0.6	0.68	0.60	**0.71**	0.61	0.70	0.62	0.68	0.62	0.69
D29	0.60	0.67	0.65	0.7	0.60	0.65	0.65	0.67	0.67	0.68	0.60	0.67	0.60	0.66	0.62	0.69	0.61	**0.71**
D30	0.62	0.65	0.61	0.68	0.6	0.66	0.62	0.65	0.6	0.65	0.63	**0.73**	0.62	0.68	0.61	0.68	0.62	0.70

Table S11: Accuracy measured by using Multivariate linear regression stepwise forward selection and Cross correlation analysis.

**ID**	GD		GDM		GDA		NM		LM		DNN		BTE		MVE		NDTF	
	**EF**	**SF**	**EF**	**SF**	**EF**	**SF**	**EF**	**SF**	**EF**	**SF**	**EF**	**SF**	**EF**	**SF**	**EF**	**SF**	**EF**	**SF**
D1	62.4	67.9	59.9	63.6	59.6	62.4	63.9	67.9	67.4	**69.2**	63	68.6	61.6	66.9	63.2	69.3	64.6	69.6
D2	63.2	69.4	59.3	64.0	58	63.9	56	62.61	63	69.9	59.8	**63.3**	62	66.7	59.6	61.3	63.6	69.8
D3	64.2	69.6	58.9	61.2	61.2	66.3	63	66.6	62.2	66.6	59.9	**65.2**	56.8	60.6	62.2	66.7	60.4	66.3
D4	63.6	66.9	56.8	60.9	62.2	66.7	61.2	66.6	60.3	63.4	66.2	69.9	56.6	60.2	61.3	63.6	61.6	**67.9**
D6	60.2	63.4	52.6	60.2	62.3	66.6	56.6	62.6	61.3	67.6	61.3	69.9	56.6	62.2	60.4	64.6	61.6	**67.3**
D6	63	69	61	69.9	56	62	56	64	60	64	59.9	**66.1**	56	59.9	61	64	63	64.8
D7	59	69	63	**66.9**	61	64	55	60	61	69	60	66	58	61.9	60	63	62	65
D8	59	63	56	60.9	62	69	64	69	63	69	66	**69.1**	52	66	62	66	66	68.1
D9	64	69.8	58	61.9	60	64	61	64	63	66	62	66	61	66	63	67	66	**69.2**
D10	56	62	54	61.9	64	69	61	69	52	66	64	**69.4**	56	66	61	69	62	68.9
D11	66	69.9	56	60.9	61	62.8	56.9	66.2	59.2	62.2	57.9	64.1	58.9	62.2	61.1	68.1	62.1	**69.8**
D12	66.2	69	58.1	62.3	58.1	66.1	53.2	64.1	56.2	66.1	59.1	66.1	58.1	60	62	66	57.6	**67.2**
D13	63.8	69.1	61.1	68.2	56.9	66.1	56	66.1	56.2	66.1	59.1	**67.9**	56.9	63.1	60.2	66	61.1	62.3
D14	60.6	66.8	59.9	62.2	59.3	68.4	60.4	68.8	61.8	67.9	58.6	**69.4**	56.4	67.8	54.3	63.3	52.8	64.9
D15	61.2	67.3	67.2	69.2	61.8	63.9	59.6	66.9	62.9	66.9	62.8	67.8	66.9	69.3	64.9	68.9	66.9	**69.3**
D16	58.2	69.3	56.1	60.3	61.2	66.8	57.1	61.6	60.9	**68.9**	61.7	63.9	56.8	60.8	61.2	65.9	61.2	66.4
D17	59.6	68.9	56.3	63.9	60.3	64.2	60.9	66.8	59.9	66.9	61.8	**67.9**	56.8	64.8	58.3	63.3	59.7	67.8
D18	56.9	62.3	56.8	62.9	58.8	66.8	61	68.1	60.8	67.9	61.8	67.3	62.8	68.2	63	68.8	63.1	**69.8**
D19	58.9	68.9	61.2	68.9	63.2	69.3	58.9	67.3	62	68.3	63	68.9	59.8	65.8	62	68.1	63.1	**69.2**
D20	59.9	64.1	60.2	66.6	61.9	68.1	58.8	66.2	63.1	69.3	58.9	**69.8**	63	67.2	63	69.8	63.8	69.3
D21	61.7	67.2	58.9	68.9	62	67.2	58.9	63.1	62.1	69.8	63	**69.8**	66.9	69.1	58.8	68.1	59.2	68.2
D22	58.9	62.2	59.2	69.2	63.3	69.2	57.3	64.2	63.1	66.9	61.2	67.1	60.8	68.1	62.2	67.6	62	**69.8**
D23	61.2	66.2	58.6	69.1	61	68.2	58.6	69.1	59.8	66.8	59.2	67.1	58.9	66.2	58.9	66.8	59.8	**69.2**
D24	63.8	68.7	60.2	69.9	61	66.9	60.7	69.1	56.9	66.7	61.9	**69.3**	59.6	66.1	62	68.1	61	68.2
D25	61.2	69	58.9	66.1	59.6	63.9	58.9	68.2	60.8	69.2	62	**69.9**	61	68.8	62	68.7	62	69.6
D26	59.9	62.8	58.6	66.2	60	68.7	62	69.1	62	68.9	61	**69.3**	61	67.8	62	69.1	61	69.1
D27	58.8	69.2	57.8	68.2	60	66.9	60	66.8	60	66.9	62	67.6	60	69.1	63	68.9	61.8	**69.9**
D28	64.8	69	61	69.2	61	67.1	60	68.6	62	68.2	63	**69.9**	66	69	63	69.6	62	68.6
D29	58.8	62.6	58.9	62.2	58.9	66.4	60.4	66.6	62.6	69.6	61	66.1	59.6	62.6	61.9	67.9	61.8	**69.8**
D30	63.2	69.8	62.2	69.2	61	68	60	66	62	66	63	**69.8**	63	68	62	66.3	61.9	69.0

Table S12: F-Measure measured by using Multivariate linear regression stepwise forward selection and Cross correlation analysis.

ID	GD		GDM		GDA		NM		LM		DNN		BTE		MVE		NDTF	
	**EF**	**SF**	**EF**	**SF**	**EF**	**SF**	**EF**	**SF**	**EF**	**SF**	**EF**	**SF**	**EF**	**SF**	**EF**	**SF**	**EF**	**SF**
D1	0.63	0.67	0.61	0.67	0.62	0.67	0.62	0.67	0.60	**0.73**	0.60	0.66	0.61	0.67	0.60	0.68	0.61	0.67
D2	0.65	0.67	0.61	0.67	0.60	0.65	0.62	0.66	0.60	**0.73**	0.61	0.67	0.61	0.69	0.60	0.68	0.61	0.69
D3	0.64	0.68	0.63	0.67	0.63	0.66	0.62	0.67	0.62	0.67	0.62	**0.71**	0.62	0.69	0.61	0.69	0.61	0.68
D4	0.61	0.66	0.62	0.66	0.6	0.66	0.62	0.67	0.62	0.67	0.6	0.65	0.60	0.68	0.60	0.69	0.61	**0.72**
D5	0.62	0.66	0.61	0.65	0.6	0.66	0.61	0.67	0.61	0.67	0.61	0.69	0.61	0.69	0.63	0.66	0.66	**0.70**
D6	0.63	0.65	0.62	0.66	0.65	0.67	0.62	0.66	0.62	0.68	0.67	**0.72**	0.67	0.69	0.62	0.68	0.66	0.70
D7	0.61	0.67	0.66	**0.71**	0.62	0.67	0.62	0.68	0.61	0.68	0.61	0.68	0.60	0.68	0.61	0.68	0.60	0.68
D8	0.61	0.67	0.62	0.68	0.65	0.68	0.62	0.68	0.63	0.67	0.68	**0.71**	0.62	0.68	0.63	0.67	0.66	0.70
D9	0.61	0.67	0.61	0.68	0.62	0.67	0.60	0.67	0.61	0.68	0.62	0.69	0.64	0.69	0.62	0.69	0.63	**0.71**
D10	0.6	0.68	0.6	0.67	0.62	0.68	0.62	0.67	0.61	0.68	0.62	**0.71**	0.62	0.67	0.61	0.68	0.61	0.69
D11	0.61	0.66	0.62	0.66	0.62	0.68	0.61	0.63	0.60	0.67	0.63	0.68	0.61	0.69	0.61	0.67	0.63	**0.70**
D12	0.62	0.66	0.60	0.67	0.60	0.67	0.61	0.65	0.61	0.68	0.61	0.68	0.61	0.69	0.62	0.67	0.67	**0.71**
D13	0.62	0.71	0.61	0.68	0.62	0.68	0.62	0.67	0.61	0.66	0.62	**0.75**	0.63	0.72	0.60	0.71	0.61	0.72
D14	0.63	0.66	0.62	0.68	0.62	0.68	0.67	0.68	0.61	0.66	0.66	**0.72**	0.66	0.71	0.66	0.70	0.69	0.69
D15	0.62	0.67	0.66	0.67	0.63	0.66	0.65	0.67	0.63	0.64	0.62	0.71	0.67	0.69	0.66	0.67	0.66	**0.76**
D16	0.63	0.67	0.62	0.68	0.62	0.66	0.60	0.65	0.66	**0.72**	0.61	0.69	0.61	0.69	0.61	0.66	0.60	0.69
D17	0.61	0.68	0.61	0.67	0.61	0.67	0.60	0.67	0.61	0.65	0.60	**0.71**	0.62	0.68	0.61	0.69	0.67	0.70
D18	0.61	0.68	0.61	0.67	0.6	0.68	0.6	0.68	0.67	0.68	0.62	0.72	0.67	0.71	0.62	0.71	0.67	**0.72**
D19	0.6	0.68	0.61	0.7	0.66	0.7	0.61	0.7	0.65	0.61	0.61	0.7	0.61	0.68	0.66	0.69	0.66	**0.71**
D20	0.62	0.67	0.60	0.68	0.60	0.68	0.62	0.7	0.61	0.66	0.6	**0.72**	0.616	0.71	0.66	0.71	0.62	0.69
D21	0.62	0.67	0.6	0.61	0.6	0.66	0.6	0.67	0.61	0.68	0.62	**0.72**	0.671	0.69	0.61	0.68	0.61	0.66
D22	0.61	0.66	0.62	0.68	0.62	0.66	0.67	0.68	0.60	0.62	0.60	0.69	0.61	0.68	0.60	0.67	0.61	**0.71**
D23	0.61	0.68	0.66	0.69	0.6	0.67	0.6	0.68	0.61	0.66	0.68	0.67	0.66	0.69	0.62	0.67	0.67	**0.70**
D24	0.61	0.66	0.6	0.67	0.6	0.66	0.6	0.66	0.65	0.68	0.61	**0.71**	0.61	0.69	0.62	0.69	0.61	0.69
D25	0.60	0.67	0.62	0.67	0.61	0.68	0.61	0.66	0.61	0.68	0.61	**0.71**	0.61	0.67	0.62	0.69	0.67	0.70
D26	0.61	0.68	0.62	0.67	0.61	0.68	0.64	0.67	0.61	0.68	0.68	**0.73**	0.67	0.70	0.62	0.67	0.67	0.72
D27	0.61	0.67	0.66	0.69	0.61	0.65	0.62	0.68	0.65	0.66	0.62	0.68	0.60	0.66	0.61	0.67	0.66	**0.73**
D28	0.61	0.68	0.62	0.7	0.62	0.68	0.65	0.68	0.61	0.69	0.61	**0.71**	0.61	0.70	0.62	0.68	0.62	0.69
D29	0.62	0.67	0.67	0.7	0.62	0.65	0.65	0.67	0.65	0.68	0.61	0.67	0.60	0.66	0.62	0.69	0.61	**0.71**
D30	0.61	0.65	0.61	0.68	0.6	0.66	0.61	0.65	0.6	0.65	0.64	**0.73**	0.62	0.68	0.61	0.68	0.62	0.70

Table S13: Accuracy measured by using Cross correlation analysis, t-test and ULR analysis.

**ID**		GD		GDM		GDA		NM		LM		DNN		BTE		MVE		NDTF
	**EF**	**SF**	**EF**	**SF**	**EF**	**SF**	**EF**	**SF**	**EF**	**SF**	**EF**	**SF**	**EF**	**SF**	**EF**	**SF**	**EF**	**SF**
D1	72.4	77.9	69.9	73.7	69.7	72.4	73.9	77.9	77.4	**79.2**	73	78.7	71.7	77.9	73.2	79.3	74.7	79.1
D2	73.2	79.4	69.3	74.0	68	73.9	67	72.71	73	79.9	69.8	**79.9**	72	77.7	69.7	71.3	73.7	79.8
D3	74.2	79.7	68.9	71.2	71.2	77.3	73	77.7	72.2	77.7	69.9	**78.2**	67.8	70.7	72.2	77.7	70.4	77.3
D4	73.7	77.9	67.8	70.9	72.2	77.7	71.2	77.7	70.3	73.4	77.2	79.9	67.7	70.2	71.3	73.7	71.7	**78.9**
D7	70.2	73.4	62.7	70.2	72.3	77.7	67.7	72.7	71.3	77.7	71.3	78.9	67.7	72.2	70.4	74.7	71.7	**79.3**
D7	73	79	71	79.2	67	72	67	74	70	74	69.9	**79.9**	67	69.9	71	74	73	74.8
D7	69	79	73	**77.9**	71	74	66	70	71	79	70	77	68	71.9	70	73	72	76
D8	69	73	67	70.9	72	79	74	79	73	79	77	**79.1**	62	77	72	77	77	78.1
D9	74	79.8	68	71.9	70	74	71	74	73	77	72	77	71	77	73	77	77	**79.2**
D10	67	72	64	71.9	74	79	71	79	62	77	74	**79.4**	67	77	71	79	72	78.9
D11	77	79.9	67	70.9	71	72.8	67.9	77.2	69.2	72.2	67.9	74.1	68.9	72.2	71.1	78.1	72.1	**79.8**
D12	77.2	79	68.1	72.3	68.1	77.1	63.2	74.1	67.2	77.1	69.1	77.1	68.1	70	72	77	67.7	**77.2**
D13	73.8	79.1	71.1	78.2	67.9	77.1	67	77.1	67.2	77.1	69.1	**77.9**	67.9	73.1	70.2	77	71.1	72.3
D14	70.7	77.8	69.9	72.2	69.3	78.4	70.4	78.8	71.8	77.9	68.7	**79.4**	67.4	77.8	64.3	73.3	62.8	74.9
D15	71.2	77.3	77.2	79.2	71.8	73.9	69.7	77.9	72.9	77.9	72.8	77.8	77.9	79.3	74.9	78.9	77.9	**79.3**
D16	68.2	79.3	67.1	70.3	71.2	77.8	67.1	71.7	70.9	**78.9**	71.7	73.9	67.8	70.8	71.2	76.9	71.2	77.4
D17	69.7	78.9	67.3	73.9	70.3	74.2	70.9	77.8	69.9	77.9	71.8	**77.9**	67.8	74.8	68.3	73.3	69.7	77.8
D18	67.9	72.3	67.8	72.9	68.8	77.8	71	78.1	70.8	77.9	71.8	77.3	72.8	78.2	73	78.8	73.1	**79.8**
D19	68.9	78.9	71.2	78.9	73.2	79.3	68.9	77.3	72	78.3	73	78.9	69.8	76.8	72	78.1	73.1	**79.2**
D20	69.9	74.1	70.2	77.7	71.9	78.1	68.8	77.2	73.1	79.3	68.9	**79.8**	73	77.2	73	79.8	73.8	79.3
D21	71.7	77.2	68.9	78.9	72	77.2	68.9	73.1	72.1	79.8	73	**79.8**	77.9	79.1	68.8	78.1	69.2	78.2
D22	68.9	72.2	69.2	79.2	73.3	79.2	67.3	74.2	73.1	77.9	71.2	77.1	70.8	78.1	72.2	77.7	72	**79.8**
D23	71.2	77.2	68.7	79.1	71	78.2	68.7	79.1	69.8	77.8	69.2	77.1	68.9	77.2	68.9	77.8	69.8	**79.2**
D24	73.8	78.7	70.2	79.9	71	77.9	70.7	79.1	67.9	77.7	71.9	**79.3**	69.7	77.1	72	78.1	71	78.2
D25	71.2	79	68.9	77.1	69.7	73.9	68.9	78.2	70.8	79.2	72	**79.9**	71	78.8	72	78.7	72	79.7
D26	69.9	72.8	68.7	77.2	70	78.7	72	79.1	72	78.9	71	**79.3**	71	77.8	72	79.1	71	79.1
D27	68.8	79.2	67.8	78.2	70	77.9	70	77.8	70	77.9	72	77.7	70	79.1	73	78.9	71.8	**79.9**
D28	74.8	79	71	79.2	71	77.1	70	78.7	72	78.2	73	**79.9**	77	79	73	79.7	72	78.7
D29	68.8	72.7	68.9	72.2	68.9	77.4	70.4	77.7	72.7	79.7	71	77.1	69.7	72.7	71.9	77.9	71.8	**79.8**
D30	73.2	79.8	72.2	79.2	71	78	70	77	72	77	73	**79.8**	73	78	72	77.3	71.9	79.0

Table S14: F-Measure measured by using Cross correlation analysis, t-test and ULR analysis.

ID	GD		GDM		GDA		NM		LM		DNN		BTE		MVE		NDTF
	**EF**	**SF**	**EF**	**SF**	**EF**	**SF**	**EF**	**SF**	**EF**	**SF**	**EF**	**SF**	**EF**	**SF**	**EF**	**SF**	**EF**	**SF**
D1	0.73	0.77	0.71	0.78	0.70	0.78	0.72	0.78	0.7	**0.83**	0.70	0.77	0.71	0.78	0.70	0.78	0.71	0.78
D2	0.73	0.77	0.71	0.77	0.7	0.77	0.71	0.77	0.7	**0.83**	0.71	0.78	0.71	0.79	0.70	0.78	0.71	0.79
D3	0.72	0.78	0.71	0.78	0.71	0.77	0.7	0.77	0.71	0.79	0.71	**0.81**	0.72	0.79	0.71	0.79	0.71	0.78
D4	0.71	0.77	0.72	0.78	0.71	0.77	0.7	0.78	0.71	0.77	0.7	0.78	0.70	0.78	0.70	0.79	0.71	**0.82**
D5	0.72	0.78	0.71	0.77	0.7	0.77	0.73	0.78	0.71	0.78	0.71	0.79	0.71	0.79	0.73	0.77	0.77	**0.80**
D6	0.71	0.76	0.7	0.77	0.73	0.78	0.70	0.77	0.70	0.79	0.78	**0.82**	0.78	0.79	0.72	0.78	0.77	0.80
D7	0.71	0.77	0.77	**0.81**	0.7	0.77	0.7	0.77	0.72	0.77	0.71	0.78	0.70	0.78	0.71	0.78	0.70	0.78
D8	0.71	0.77	0.72	0.78	0.76	0.78	0.72	0.78	0.71	0.76	0.77	**0.81**	0.72	0.78	0.73	0.78	0.77	0.80
D9	0.71	0.77	0.71	0.78	0.72	0.77	0.7	0.78	0.71	0.79	0.73	0.79	0.74	0.79	0.72	0.79	0.73	**0.81**
D10	0.72	0.78	0.7	0.77	0.72	0.78	0.7	0.77	0.71	0.78	0.72	**0.81**	0.72	0.78	0.71	0.78	0.71	0.79
D11	0.71	0.77	0.70	0.77	0.70	0.78	0.71	0.73	0.70	0.78	0.72	0.78	0.71	0.79	0.71	0.78	0.73	**0.80**
D12	0.72	0.77	0.70	0.77	0.70	0.77	0.71	0.76	0.71	0.78	0.71	0.78	0.71	0.79	0.72	0.78	0.78	**0.81**
D13	0.70	0.81	0.71	0.78	0.7	0.78	0.72	0.77	0.71	0.77	0.72	**0.85**	0.73	0.82	0.70	0.81	0.71	0.82
D14	0.71	0.77	0.72	0.77	0.74	0.78	0.76	0.78	0.71	0.76	0.76	**0.82**	0.77	0.81	0.77	0.80	0.79	0.79
D15	0.72	0.78	0.77	0.78	0.71	0.77	0.76	0.78	0.71	0.78	0.71	0.81	0.78	0.79	0.77	0.78	0.77	**0.87**
D16	0.73	0.77	0.72	0.78	0.70	0.77	0.72	0.76	0.77	**0.82**	0.71	0.79	0.71	0.79	0.71	0.77	0.70	0.79
D17	0.71	0.78	0.71	0.77	0.71	0.77	0.71	0.77	0.71	0.77	0.71	**0.81**	0.72	0.78	0.71	0.79	0.78	0.80
D18	0.71	0.78	0.73	0.77	0.72	0.78	0.72	0.78	0.76	0.78	0.73	0.82	0.78	0.81	0.72	0.81	0.78	**0.82**
D19	0.70	0.78	0.71	0.80	0.77	0.78	0.71	0.8	0.76	0.71	0.72	0.8	0.71	0.78	0.77	0.79	0.77	**0.81**
D20	0.72	0.77	0.70	0.78	0.70	0.78	0.74	0.8	0.72	0.77	0.7	**0.82**	0.717	0.81	0.77	0.81	0.72	0.79
D21	0.72	0.77	0.7	0.7	0.7	0.76	0.7	0.7	0.7	0.79	0.78	**0.82**	0.781	0.79	0.71	0.78	0.71	0.77
D22	0.71	0.77	0.7	0.78	0.7	0.77	0.76	0.78	0.72	0.72	0.70	0.79	0.71	0.78	0.70	0.78	0.71	**0.81**
D23	0.71	0.78	0.77	0.8	0.7	0.8	0.7	0.77	0.76	0.77	0.78	0.78	0.77	0.79	0.72	0.78	0.78	**0.80**
D24	0.72	0.76	0.7	0.7	0.7	0.76	0.73	0.77	0.76	0.78	0.70	**0.81**	0.71	0.79	0.72	0.79	0.71	0.79
D25	0.7	0.75	0.7	0.75	0.71	0.77	0.7	0.76	0.71	0.78	0.71	**0.81**	0.71	0.78	0.72	0.79	0.78	0.80
D26	0.71	0.73	0.7	0.7	0.7	0.75	0.72	0.77	0.7	0.76	0.77	**0.83**	0.78	0.80	0.72	0.78	0.78	0.82
D27	0.71	0.75	0.77	0.70	0.7	0.76	0.72	0.77	0.77	0.78	0.71	0.79	0.70	0.77	0.71	0.78	0.77	**0.83**
D28	0.71	0.7	0.7	0.8	0.7	0.77	0.76	0.78	0.70	0.79	0.71	**0.81**	0.71	0.80	0.72	0.78	0.72	0.79
D29	0.71	0.76	0.75	0.8	0.7	0.75	0.76	0.77	0.78	0.79	0.71	0.78	0.70	0.77	0.72	0.79	0.71	**0.81**
D30	0.71	0.76	0.71	0.75	0.70	0.75	0.7	0.7	0.7	0.77	0.74	**0.83**	0.72	0.78	0.71	0.78	0.72	0.80
